# Jiangtang Sanhao formula ameliorates skeletal muscle insulin resistance *via* regulating GLUT4 translocation in diabetic mice

**DOI:** 10.3389/fphar.2022.950535

**Published:** 2022-09-08

**Authors:** Zimengwei Ye, Jinkun Ma, Yage Liu, Bingrui Xu, Xuan Dai, Min Fu, Tian Tian, Xin Sui, Fangfang Mo, Sihua Gao, Dandan Zhao, Dongwei Zhang

**Affiliations:** ^1^ School of Traditional Chinese Medicine, Beijing University of Chinese Medicine, Beijing, China; ^2^ Research Institute of McGill University Health Center, McGill University, Montreal, QC, Canada; ^3^ Information and Educational Technology Center, Beijing University of Chinese Medicine, Beijing, China

**Keywords:** Jiangtang Sanhao formula (JTSHF), diabetes mellitus, insulin resistance, GLUT4, AMPKα/SIRT1/PGC-1α signaling pathway, Chinese medicine

## Abstract

Jiangtang Sanhao formula (JTSHF), one of the prescriptions for treating the patients with diabetes mellitus (DM) in traditional Chinese medicine clinic, has been demonstrated to effectively ameliorate the clinical symptoms of diabetic patients with overweight or hyperlipidemia. The preliminary studies demonstrated that JTSHF may enhance insulin sensitivity and improve glycolipid metabolism in obese mice. However, the action mechanism of JTSHF on skeletal muscles in diabetic mice remains unclear. To this end, high-fat diet (HFD) and streptozotocin (STZ)-induced diabetic mice were subjected to JTSHF intervention. The results revealed that JTSHF granules could reduce food and water intake, decrease body fat mass, and improve glucose tolerance, lipid metabolism, and insulin sensitivity in the skeletal muscles of diabetic mice. These effects may be linked to the stimulation of GLUT4 expression and translocation via regulating AMPKα/SIRT1/PGC-1α signaling pathway. The results may offer a novel explanation of JTSHF to prevent against diabetes and IR-related metabolic diseases.

## 1 Introduction

Diabetes mellitus (DM), a chronic metabolic disease, has emerged as one of the major concerns exceedingly threatening to physical and mental health for humans throughout the world (Pang et al., 2020). The International Diabetes Federation has estimated that 783 million people aged 20–79 years will suffer from DM by 2045 (Sun et al., 2022), and type 2 diabetes mellitus (T2DM) accounts for a sea of proportion (approximately 90%) of the suffers (Thomas and Philipson, 2015; Xu et al., 2018; Tanase et al., 2020). It is generally accepted that insulin resistance (IR) and islet β-cell dysfunction remain the core deficit of T2DM (Bai et al., 2015; Inaishi and Saisho, 2020), while IR has emerged as a decisive pathophysiological contributor in the development of T2DM (Sampath Kumar et al., 2019).

The integrative physiology of IR is mainly attributed to the defective insulin action at peripheral target tissues (skeletal muscle, liver, and adipose tissue). Among them, skeletal muscle, a vital organ for glucose clearance, taking charge of clearing approximately 75%–80% of postprandial glucose uptake and 95% of glucose uptake under hyperglycemia (Hagiwara, 2014), is deemed as the primary driver for the development of systemic IR (Carnagarin et al., 2015; Merz and Thurmond, 2020; Pyun et al., 2021). Consequently, remediating IR in skeletal muscle alone is sufficient to maintain systemic glucose homeostasis (Abdul-Ghani and DeFronzo, 2010; Merz and Thurmond, 2020). Several studies have confirmed the IR in skeletal muscle is the initial defect in T2DM that may appear decades before β-cell dysfunction and significant hyperglycemia development (DeFronzo and Tripathy, 2009; Merz and Thurmond, 2020). Accumulating evidence suggests that glucose transporter 4 (GLUT4) is the rate-limiting step of glucose uptake in skeletal muscle cells, and the impaired GLUT4 translocation is the principal defect of IR in skeletal muscle (Richter and Hargreaves, 2013; Sanvee et al., 2019; Herman et al., 2022). Several classic pathways have been found to be involved in the translocation mechanism of GLUT4, such as phosphoinositide-3-kinase (PI3K)/protein kinase B (AKT) signaling pathway, adenosine monophosphate-activated protein kinase (AMPK) signaling pathway, and so on. PI3K/AKT pathway has been vindicated to be a canonical insulin signaling pathway, and its activation occurs when insulin receptor substrate 1 (IRS1) tyrosine is directly phosphorylated after insulin binds to insulin receptor. The IRS1 combines with PI3K to activate downstream protein AKT, which ultimately promotes the expression and translocation of GLUT4 (Wang et al., 2020a; Fazakerley et al., 2022). AMPK, a crucial regulatory protein keeping the balance of systemic energy metabolism, is essential for maintaining glucose balance and has been proven to be a druggable and potentially therapeutic target for the treatment of IR and T2DM (Entezari et al., 2022), and its activation promotes the translocation of GLUT4 vesicles from intracellular membranes to the cell surface (Zhang et al., 2018b; Chen et al., 2021b; Shrestha et al., 2021). PI3K/AKT signaling pathway exerts a supreme effect in insulin-stimulated glucose transport (Guo et al., 2019). While AMPK has been validated to be a pivotal signal pathway that regulates glucose uptake in an insulin independent manner (Leclerc and Rutter, 2004; Elhassan et al., 2018; Miyamoto, 2018). It has been reported that drugs such as metformin (Rena et al., 2017) , exercise/contraction, and some other metabolic stress are able to activate AMPK and consequently promote GLUT4 expression and translocation (Moghetti et al., 2016; Distefano and Goodpaster, 2018; Richter, 2021; Entezari et al., 2022). Additionally, muscle mitochondrial dysfunction is closely associated with T2DM, which adversely influences the translocation of GLUT4, resulting in the IR of skeletal muscle (Yu et al., 2018; Lee et al., 2020; Meng et al., 2020). Among them, the role of AMPK in promoting GLUT4 translocation by regulating mitochondrial function has been brought into the limelight. For example, AMPK may improve insulin sensitivity and GLUT4 translocation via stimulating peroxisome proliferator activated receptor-γ coactivator (PGC-1α), a mitochondrial biosynthesis-related gene (Herzig and Shaw, 2018), and simultaneously affects a string of associated regulators accelerating mitochondrial synthesis (Bai et al., 2021), such as peroxisome proliferator activated receptor α (PPARα), and silencing regulatory protein sirtuin1 (SIRT1). Therefore, mitochondrial dysfunction may bring about a huge impact on GLUT4 translocation, thereby aggravating IR through affecting glucose uptake, transport, and utilization.

The intervention of traditional Chinese medicine (TCM) to DM is on the basis of syndrome differentiation, taking the holistic concept as a foothold, and aim at maximizing efficacy and minimizing toxicity (Wang et al., 2020b; Pang et al., 2020). In recent years, many investigators have strived to clarify the pathogenesis of DM from the aspects of viscera and qi-blood theory (Xu et al., 2019b; Zheng et al., 2020). The theory of “treating T2DM based on syndrome differentiation of liver, spleen, and kidney” was firstly proposed by professor Sihua Gao, who pointed out that the essential pathogenesis characteristic of T2DM in TCM was the dysfunctions of liver, spleen, and kidneys (Gao et al., 2009). Based on years of clinical experiences, professor Gao established a prescription for diabetes treatment—Jiangtang Sanhao formula (JTSHF), comprised of *Panax ginseng* C. A. Meyer. (Araliaceae; Ginseng Radix et Rhizoma), *Dioscorea oppositifolia* L. (Dioscoreaceae; Dioscoreae Rhizoma), *Coptis chinensis* Franch. (Ranunculaceae; Coptidis Rhizoma), etc. (Bai et al., 2020). The function of JTSHF is harmonizing the function of liver, spleen and kidneys by regulating the spleen principally. According to Zang Xiang theory in TCM, the spleen governs the muscles and the abnormal exertion of muscle function is subjected to the dysfunction of spleen. Therefore, skeletal muscle was selected as the target tissue in this study. Moreover, the main active ingredients of JTSHF, such as ginsenoside Rb1 and the berberine, have been reported to improve the insulin sensitivity in skeletal muscle (Tabandeh et al., 2017; Yu et al., 2018; Mi et al., 2019; Xu et al., 2020), with an acting mechanism of stimulating the expression or translocation of GLUT4 through activating the AMPK signaling pathway (Kong et al., 2009; Bai et al., 2021). For example, a molecular docking study on berberine showed that berberine stimulated the Thr172 phosphorylation of AMPK through hydrophobic interaction with LysA29, an amino acid residue of AMPK, thereby activating the AMPK signaling pathway (Li et al., 2021). The results from decades of TCM clinical trials indicated that JTSHF granules have exerted gratifying therapeutic effects on hypoglycemia among diabetic patients (Gao et al., 2009). And the preclinical studies have shown that JTSHF could correct the disorder of glycolipid metabolism in obese mice by activating PI3K/AKT signaling pathway (Bai et al., 2019). In addition, it could relieve the body weight, reduce food intake and alleviate IR in obese mice via affecting the protein expressions relating to hypothalamic feeding central neurons and the structural protection and functional restoration in pancreatic tissue (Bai et al., 2020). However, whether and how JTSHF improve IR in skeletal muscle during diabetes remain unclear. Therefore, the current study is aimed to investigate the effect of JTSHF on improving skeletal muscles IR in diabetic mice induced by HFD and streptozotocin (STZ), and to probe whether the compound could relieve IR via stimulating AMPKα/SIRT1/PGC-1α signaling pathway to modulate GLUT4 translocation.

## 2 Materials and methods

### 2.1 Animal

8-week-old C57BL/6N male mice were purchased from Beijing Vital River Laboratory Animal Technology Co., Ltd. with license No. SCXK (Beijing) 2016-0011. All mice were housed in the barrier environment Animal Laboratory of Beijing University of Chinese medicine under specific conditions (temperature: 21–25°C, humidity: 45%–65%, and a 12 h light/dark cycle), and with free access to food and water. The experiment protocol was approved by the Animal Care Committee of Beijing University of Chinese medicine (No. BUCM-4-2021032502-1076).

### 2.2 Drugs

JTSHF [composed of *Panax ginseng* C. A. Meyer. (Araliaceae; Ginseng Radix et Rhizoma), *Atractylodes macrocephala* Koidz. (Asteraceae; Atractylodis macrocephalae Rhizoma), *Dioscorea oppositifolia* L. (Dioscoreaceae; Dioscoreae Rhizoma), *Bupleurum falcatum* L. (Apiaceae; Bupleuri Radix), *Smilax glabra* Roxb. (Smilacaceae; Poria), *Citrus aurantium* L. (Rutaceae; Aurantii Immaturus Fructus), *Rehmannia glutinosa* (Gaertn.) DC. (Orobanchaceae; Rehmanniae Radix), *Coptis chinensis* Franch. (Ranunculaceae; Coptidis Rhizoma), *Salvia miltiorrhiza* Bunge (Lamiaceae; Salviae Miltiorrhizae Radix et Rhizoma), *Epimedium sagittatum* (Siebold & Zucc.) Maxim. (Berberidaceae; Epimedii Herba) in the ratio of 6:3:2:2:2:3:2:6:6:2] granules were purchased from Yifang Pharmaceutical Co., Ltd. (Beijing, China), and the suspension with corresponding concentration was made from distilled water before administration. Metformin hydrochloride tablets were from Sino-American Shanghai Squibb Pharmaceutical Co., Ltd. (Shanghai, China), and prepared into suspension with sterilized water before intragastric administration. STZ was purchased from Sigma-Aldrich (Saint Louis, MO, United States) and stored at −20°C before use. Insulin injection was bought from Novo Nordisk (Copenhagen, Denmark).

### 2.3 Reagents

The BCA protein content detection kit was bought from KeyGen Biotech (Nanjing, China). Hypersensitive ECL chemiluminescence kit, protease, and phosphatase inhibitor were bought from NCM Biotech (Suzhou, China). The protein extraction kit was obtained from Beyotime Institute of Biotechnology (Shanghai, China). Non-esterified fatty acids (NEFA) and blood lipid test kits were purchased from Nanjing Jiancheng Bioengineering Institute (Nanjing, China). Revert Aid First Strand cDNA Synthesis Kit was purchased from Thermo Fisher Scientific (Waltham, MA, United States). Power SYBR Green PCR master mix was from Invitrogen (Carlsbad, CA, United States). The primers of AMPKα, SIRT1, PGC-1α, PPARα, GLUT4, UCP3, and β-actin were designed and synthesized by Sangon Biotech (Shanghai, China). Both primary antibodies to AMPKα, PGC-1α, PPARα, GLUT4, UCP3, GAPDH, Lamin B1, and Na, K-ATPase (Cat no: 66536-1-Ig, 66369-1-Ig, 15540-1-AP, 66846-1-Ig, 10750-1-AP, 60004-1-Ig, 66095-1-Ig, and 14418-1-AP) and secondary antibodies (Cat no: SA00001-1 and SA00001-2) were purchased from the Proteintech Group (Chicago, IL, United States). The antibody to Phospho-AMPKα (Thr172, Cat #: 50081S) was purchased from the Cell Signaling Technology (Boston, MA, United States). The antibody to SIRT1 (Cat #: ab189494) was purchased from Abcam (Cambridge, United Kingdom).

### 2.4 Methods

#### 2.4.1 Type 2 diabetes mellitus mice modeling and drug intervention

**FIGURE 1:**
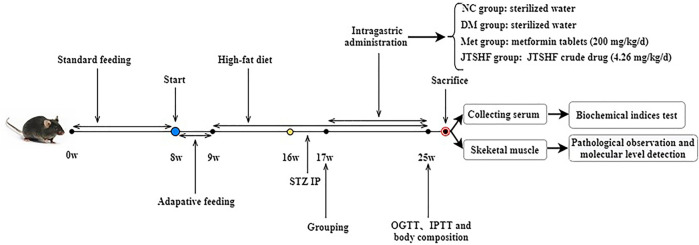
The flow chart of the animal study.

After 1 week of adaptive feeding, C57BL/6N male mice were randomly divided into the normal control group (NC; 3.1 kcal/g of heat quantity; SBF Biotechnology Co. Ltd., Beijing, China) and HFD-fed group (4.73 kcal/g of heat quantity; Medicience biomedical Co. Ltd., Jiangsu, China). The energy composition of HFD is protein (20% Kcal), fat (45% Kcal), and carbohydrate (35% Kcal). After exposure to the respective diets for 7 consecutive weeks, mice given HFD were injected intraperitoneally with STZ (100 mg/kg body weight) freshly dissolved in 0.1M citric acid-sodium citrate buffer (pH = 4.3–4.5) to induce T2DM (Han et al., 2021), whereas mice in the NC group received an intraperitoneal injection of the same volume of citrate buffer 3 days after STZ injection, the blood glucose was measured after 12-hour fasting, and mice treated with HFD-STZ whose fasting blood glucose (FBG) was higher than 11.1mM were considered as T2DM models (Liu et al., 2019). All T2DM mice were randomly subdivided into 3 groups (*n = 8* per group): diabetic model (DM) group, metformin (Met) group, and JTSHF group. Mice in the JTSHF group and Met group were orally given JTSHF granules (4.26 g/kg/d) and metformin hydrochloride tablets (200 mg/kg/d) (Han et al., 2021), respectively. Doses of JTSHF administrated to mice were determined on the basis of the clinical equivalent doses referred to the methods in Experimental methodology of pharmacology (Xu et al., 2003) and the results of the preliminary experiment. And mice in both the NC and DM groups were orally given the equal volume of sterilized water. The concrete experimental design flow chart is shown in Figure 1. During the intervention, the body weight, FBG, water and food intake of each mouse were recorded once a week. The body composition of each mouse was evaluated by a nuclear magnetic resonance animal body composition analyzer (NIUMAG Co. Ltd., Shanghai, China).

#### 2.4.2 Oral glucose tolerance test and intraperitoneal insulin tolerance test

Before Oral glucose tolerance test (OGTT) assay, mice were fasted overnight and then gavaged with 2 g/kg body weight of glucose dose (Jung et al., 2018). To perform intraperitoneal insulin tolerance test (IPITT), mice were fasted from 9:00 a.m. to 2:00 p.m. before intraperitoneal injection of insulin (Actrapid; Novo Nordisk, Copenhagen, Denmark) at the dose of 0.5 U/kg body weight (Bai et al., 2021). The glucose levels in mouse tail blood were measured at time points of 0, 30, 60, 90, and 120 min post gavage or injection. The area under the curve (AUC) for glucose contents was calculated according to the equation (Leng et al., 2014): AUC (mmol/L*h) = 0.5 h × (BG0min + BG30min)/2 + 0.5 h × (BG30min + BG60min)/2 + 1 h × (BG60min + BG120min)/2, and BG refers to blood glucose.

#### 2.4.3 Specimen collection

After 8 weeks intervention, mice fasted overnight were injected with 1% sodium pentobarbital for anesthesia (Li et al., 2019). Blood was taken from the heart, and the serum was collected after centrifugation (3000 r/min, 4°C for 15 min) and stored at −80°C for the following experiments. Subsequently, skeletal muscles on both sides of the hind limb were immediately removed from the body, which were either fixed in 4% paraformaldehyde or stored at −80°C for the subsequent experiments.

#### 2.4.4 Biochemical analysis

Serum triglyceride (TG), total cholesterol (TC), low-density lipoprotein cholesterol (LDL-C), high-density lipoprotein cholesterol (HDL-C), non-esterified fatty acid (NEFA), and serum insulin (INS) levels were achieved and analyzed through an appropriate kit in accordance with the manufacturer's instructions.

#### 2.4.5 Western blot analysis

Western blot electrophoresis system (Bio-Rad, Hercules, CA, United States) was used for analyzing relevant proteins expression levels. Subsequent procedures were implemented according to the protocols published before (Gadallah et al., 2021; Lee et al., 2021; Lu et al., 2021). Briefly, polyvinylidene fluoride (PVDF) membranes were incubated with primary antibodies of AMPKα, PPARα (1:500), p-AMPKα (1:1000), SIRT1, PGC-1α, GLUT4 (1:1000), and UCP3 (1:2000) at 4°C overnight. The next day, secondary antibodies were incubated (1:5000) at room temperature for 1.5 h. Finally, the hypersensitive ECL was applied to color rendering, then a gel imager (Azure Biosystems, Dublin, CA, United States) were used to expose imaging. The stripe gray value was analyzed by Image J.

#### 2.4.6 Real-time PCR analysis

The total RNA was extracted with Trizol extraction reagent, and subjected to determinate the concentration using the Fluostar Omega multimode reread (BMG LabTech, Offenburg, Germany). After reverse transcription, the amplification was performed with the Real-time PCR (RT-PCR) instrument (Applied Biosystems, Waltham, MA, United States). The reaction parameters were the followings: 1) pre-denaturation at 95°C for 10 min; 2) 40 amplification cycles of denaturing at 95°C for 15 s and annealing at 60°C for 1 min; 3) amplification of dissolution curve: 95°C for 15 s, 60°C for 1 min, 95°C for 15 s. Calculations for the relative qualification (RQ) of the target gene were performed by 2^−△△Ct^ (Guo et al., 2020). The forward/reversed primer sequences for each objective gene were demonstrated in Table 1.

**TABLE 1:** Sequences of the objective gene primers.

Objective gene	Forward/reversed primer sequences (5′-3′)
AMPKα	F: GTC​CTG​CTT​GAT​GCA​CAC​AT R: GAC​TTC​TGG​TGC​GGC​ATA​AT
SIRT1	F: AGT​TCC​AGC​CGT​CTC​TGT​GT R: GAA​CGG​CTT​CCT​CAG​GTT​CTT
PGC-1α	F: CCC​TGC​CAT​TGT​TAA​GAC​C R: TGC​TGC​TGT​TCC​TGT​TTT​C
PPARα	F: GCG​TAC​GGC​AAT​GGC​TTT​AT R: GAA​CGG​CTT​CCT​CAG​GTT​CTT
GLUT4	F: CTT​AGG​GCC​AGA​TGA​GAA​TGA​C R: ACA​GGG​AAG​AGA​GGG​CTA​AA
UCP3	F: GAG​TCT​CAC​CTG​TTT​ACT​GAC​A R: CGT​TCA​TGT​ATC​GGG​TCT​TTA​C

#### 2.4.7 Hematoxylin-eosin staining

One part of the gastrocnemius muscle fixed in 4% paraformaldehyde was embedded in paraffin, and sectioned at a thickness of 5 μm. Then the sections were performed with hematoxylin-eosin (H&E) staining according to the routine procedure (Wang et al., 2017). Finally, the histopathological changes in muscle tissue were observed under an inverted microscope (Olympus Corporation, Tokyo, Japan).

#### 2.4.8 Immunohistochemical staining analysis

The immunohistochemical (IHC) staining was conducted with the set routine (Guo et al., 2020). Paraffin sections of skeletal muscle were processed by dewaxing, and 3% H_2_O_2_ was added to block endogenous peroxidase. After the antigen retrieval and goat serum blocking, the sections were incubated with primary antibody of GLUT4 (1:200) and secondary antibody, then followed by DAB exposure, hematoxylin counterstaining, gradient ethanol dehydration, vitrification, sealing, and microscopic examination. Image Pro Plus 6.0 was applied to analyze the intensity of positive staining (brown granules) in skeletal muscle.

#### 2.4.9 Statistical analysis

GraphPad Prism 7 was used for data analysis and cartography, with data expressed as mean ± standard error of mean (SEM). The difference among multiple groups was compared with the analysis of variance (ANOVA) method, and the Dunnett test was set aside for posting multiple comparisons. *p* < 0.05 was indicated a statistical difference among groups.

## 3 Results

### 3.1 Jiangtang Sanhao formula reduced calorie and water intake and improved body composition in diabetic mice

**FIGURE 2:**
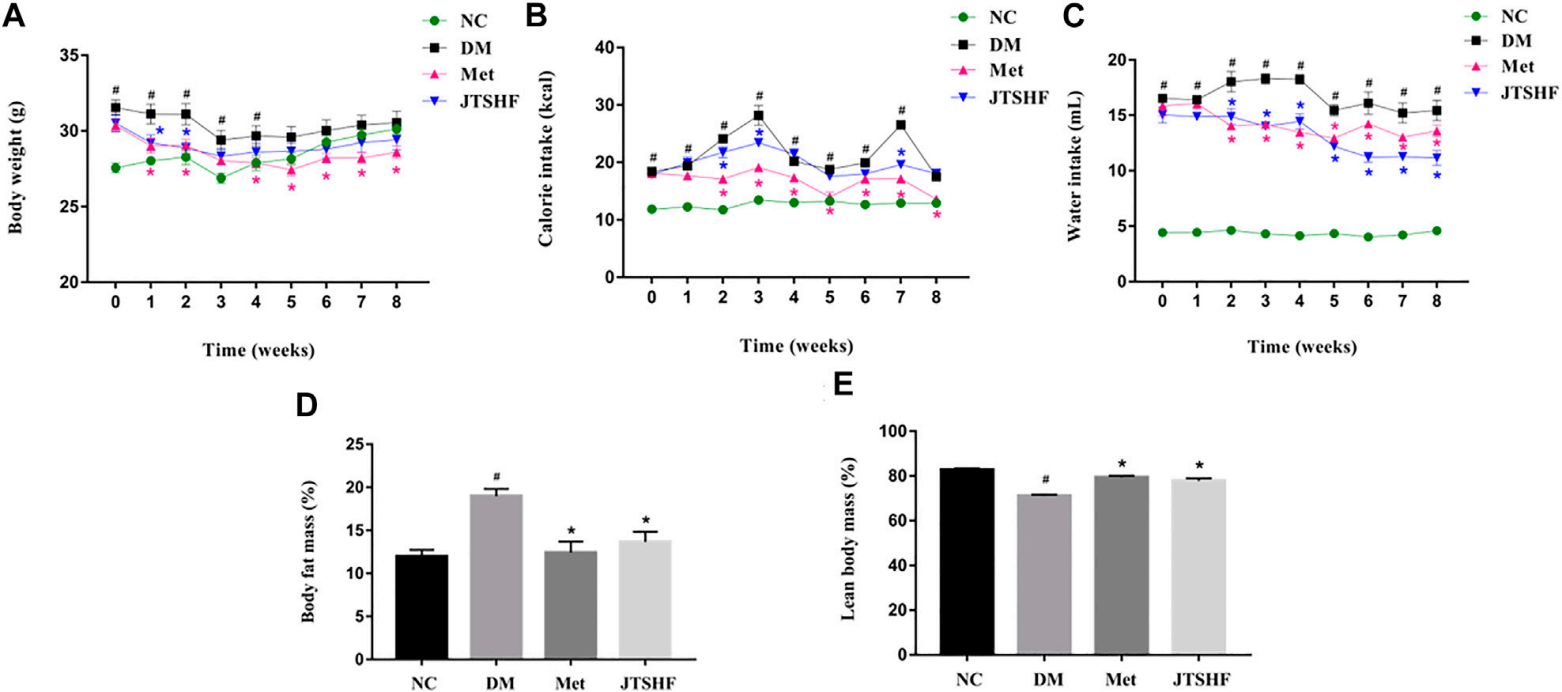
JTSHF reduced calorie and water intake and improved body composition in diabetic mice. **(A)** Body weight of mice. Alteration in **(B)** calorie intake and **(C)** water intake during intervention. **(D)** Body fat mass and **(E)** lean body mass of mice after treatment. All Values were expressed as mean ± SEM. ^#^ *p* < 0.05 versus NC group, ^*^ *p* < 0.05 versus DM group; *n* = 8 for each group in A–E. Time in A–C means time of drug administration. The calorie intake of each mouse was calculated by the following formula: calorie intake in normal mice = food intake/g × 3.1 kcal/g, calorie intake in HFD-STZ induced diabetic mice = food intake/g × 5.24 kcal/g.

As shown in Figures 2A,B, the body weight of mice in the DM group was obviously higher than that in the NC group in the early stage (0 to 4th week) of administration (*p* < 0.05), but no significant difference were found in body weight between these two groups in the late phase of administration (*p* > 0.05). Compared with the DM group, the body mass and calorie intake of the diabetic mice following metformin treatment were significantly decreased (*p* < 0.05). However, we failed to find an obvious body weight loss in the JTSHF treated mice compared with their peers in the DM group, but JTSHF granules reduced calorie intake in diabetic mice at the 2nd, 3rd, and 7th weeks. Figure 2C showed that metformin or JTSHF distinctively decreased the water intake in diabetic mice after 8 weeks of treatment. The body composition results revealed that mice in the Met and JTSHF groups presented less body fat mass and higher lean body mass than that in the DM group (Figures 2D,E, *p* < 0.05). Therefore, the results suggest that JTSHF could ameliorate the polydipsia, reduce body fat mass, and enhance lean body mass in diabetic mice.

### 3.2 Jiangtang Sanhao formula improved glucose tolerance and alleviated insulin resistance in diabetic mice

**FIGURE 3:**
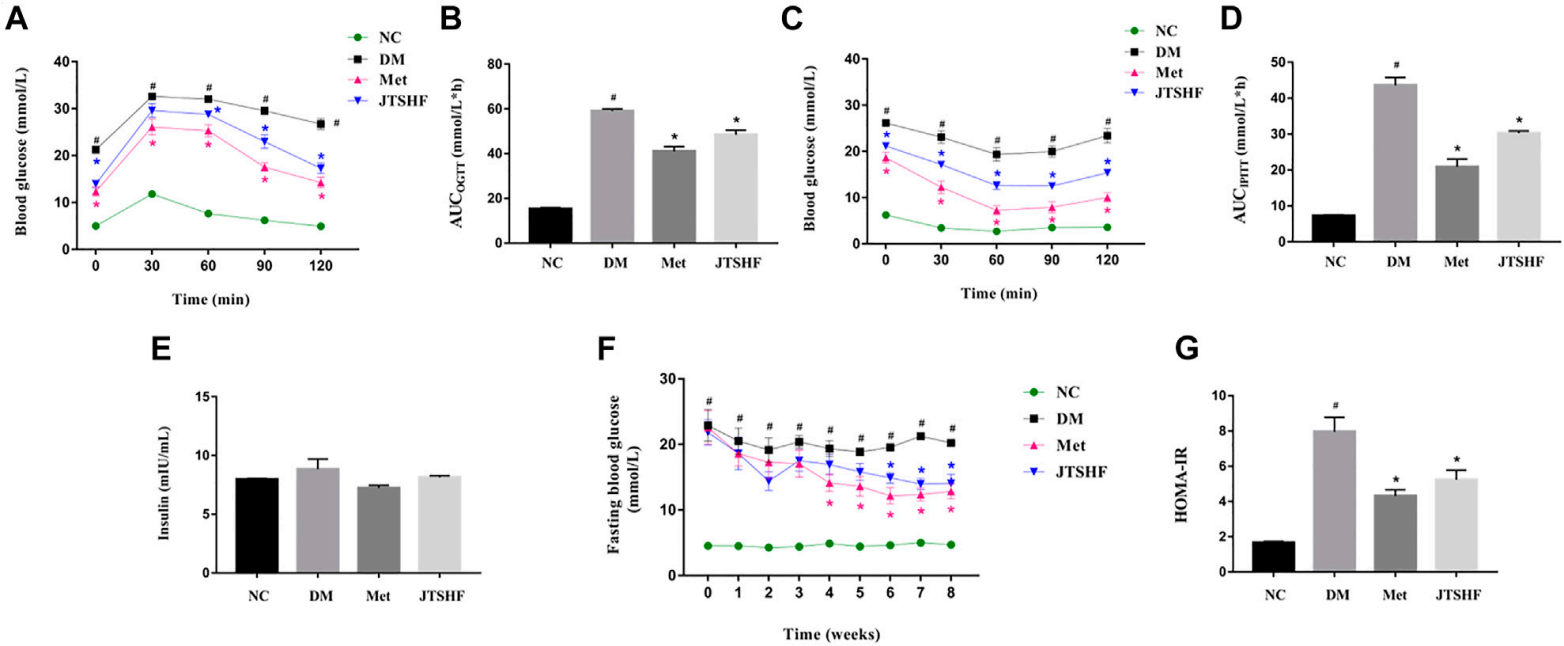
JTSHF improved glucose tolerance and enhanced insulin sensitivity in HFD-STZ induced diabetic mice. **(A)** OGTT and **(B)** area under the curve (AUC). **(C)** IPITT and **(D)** AUC. **(E)** fasting serum insulin levels and **(F)** fasting blood glucose levels, **(G)** Insulin resistance index of homeostasis model assessment (HOMA-IR). HOMA-IR = fasting insulin level (mU/L)×fasting glucose level (mmol/L)/22.5 (Zhang et al., 2018a). All values were expressed as mean ± SEM. ^#^ *p* < 0.05 versus NC group, ^*^ *p* < 0.05 versus DM group; *n* = 8 for each group in A–-F. Time in F means time of drug administration.

In order to further observe the effect of JTSHF on glucose tolerance and insulin sensitivity in diabetic mice, OGTT and IPITT assays were performed at the 7th and 8th weeks of administration, respectively. OGTT results showed that the blood glucose levels in each group of the mice peaked at 30 min after glucose loading, then had a slow downward trend. However, the mice in the DM group displayed a more evident decrease in glucose tolerance than that of normal mice, while the impaired glucose intolerance was improved in the diabetic mice treated with metformin or JTSHF after 7 weeks of drug administration (Figure 3A, *p* < 0.05). Moreover, from the OGTT-AUC results (Figure 3B), the AUC of the DM group (59.11 ± 0.85) was significantly higher than that of the normal mice (15.32 ± 0.55), and the AUC of all treated groups was decreased compared with the DM group (the AUC of OGTT in Met and JTSHF groups was 41.11 ± 2.00 and 48.5 ± 1.94, respectively). In IPITT assay, the effect of insulin on glucose clearance in diabetic mice was significantly lower than that in control mice, while metformin or JTSHF responded to the hypoglycemic efficiency of insulin after intraperitoneal injection of insulin, and had a remarkable clearance of their endogenous glucose compared with the mice in the DM group (Figures 3C,D, *p* < 0.05). In addition, as shown in Figure 3E, after 8 weeks of intervention, the DM group showed a slight increase of fasting serum insulin levels compared with the other groups, but the difference among groups was of no statistical significance (*p* > 0.05). Compared with the DM group, the FBG of the diabetic mice in the last 3 weeks of administration following JTSHF treatment were significantly decreased (Figure 3F, *p* < 0.05). Furthermore, it should be noted that the HOMA-IR index of diabetic mice increased significantly, while metformin or JTSHF could reverse these alterations (Figure 3G, *p* < 0.05). Taken together, these results point out that treatment with JTSHF granules could notably lower IR and restore disordered glucose metabolism.

### 3.3 Jiangtang Sanhao formula improved serum lipid profiles and pathological morphology of skeletal muscle in diabetic mice

**FIGURE 4:**
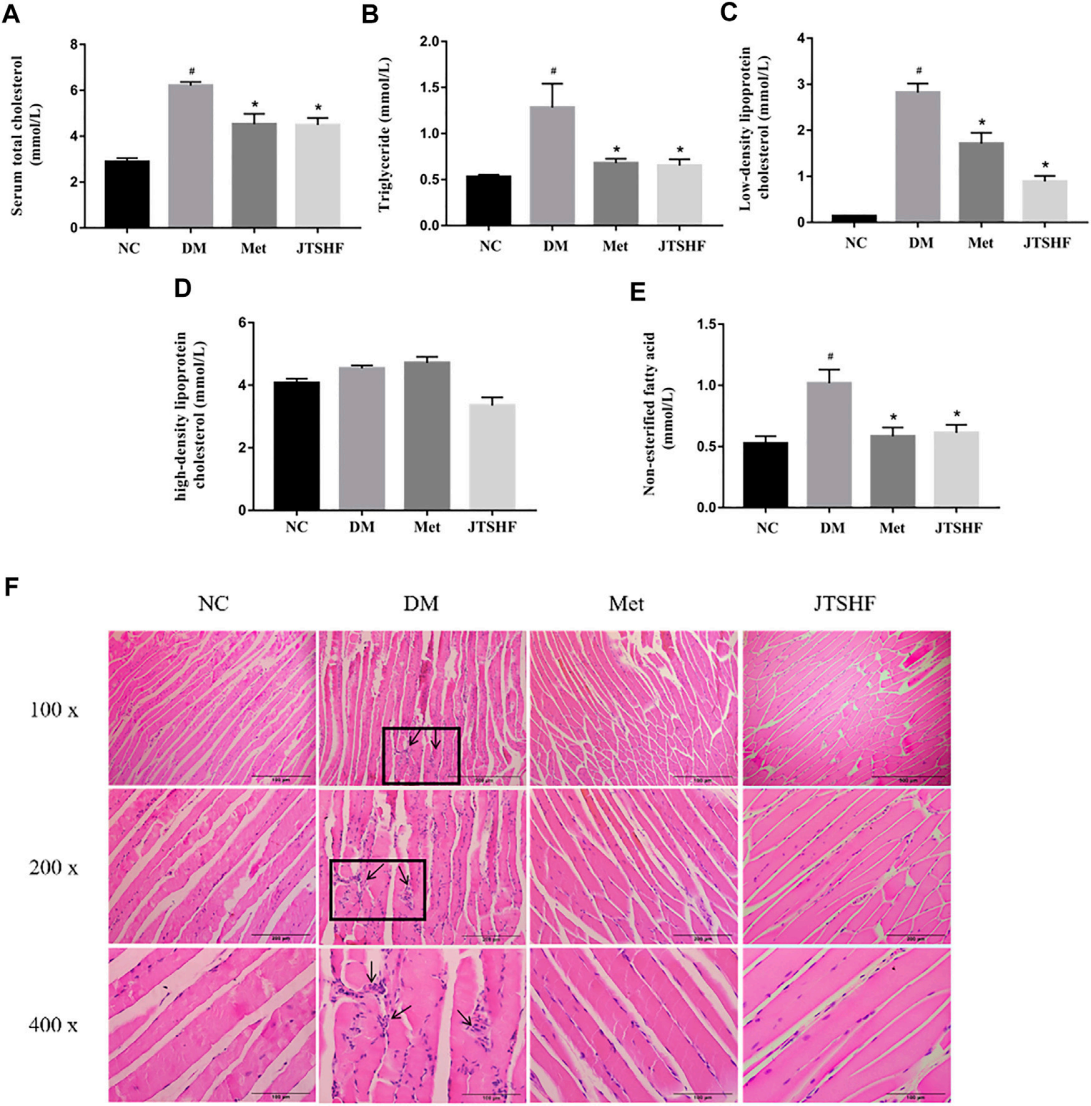
JTSHF could improve abnormal lipid profile in HFD-STZ induced diabetic mice. **(A)** Serum total cholesterol, **(B)** triglyceride, **(C)** low-density lipoprotein cholesterol, **(D)** high-density lipoprotein cholesterol, **(E)** non-esterified fatty acid of mice in each group. **(F)** HE staining of skeletal muscle tissue (100 x, 200 x, 400 x). All Values were expressed as mean ± SEM. ^#^ *p* < 0.05 versus NC group, ^*^ *p* < 0.05 versus DM group; *n* = 8 for each group in A–E.

Serum levels of TG, TC, LDL-C, HDL-C, and NEFA were measured to further evaluate the effect of JTSHF granules on dyslipidemia in diabetic mice in the end of the experiment. Serum chemical analysis turned out that the levels of TC, TG, LDL-C, and NEFA in the DM group were considerably higher than those in the NC group (Figures 4A–C,E, *p* < 0.05), while dyslipidemia in diabetic mice was effectively ameliorated following JTSHF or metformin treatment (*p* < 0.05). However, there was no significant difference in the serum levels of HDL-C among each group (Figure 4D, *p* > 0.05). H&E staining of skeletal muscle tissue showed that myofibrils of the DM group were disorganized, accompanied by multiple edema and intense inflammatory infiltration. On the contrary, less edematous tissue and indistinctively inflammatory infiltration were observed in the JTSHF and Met groups (Figure 4F). The above results indicate that JTSHF could partly correct dyslipidemia and reduce pathological injury in skeletal muscles of diabetic mice.

### 3.4 Jiangtang Sanhao formula facilitated GLUT4 translocation by activating AMPKα/SIRT1/PGC-1α signaling pathway

**FIGURE 5:**
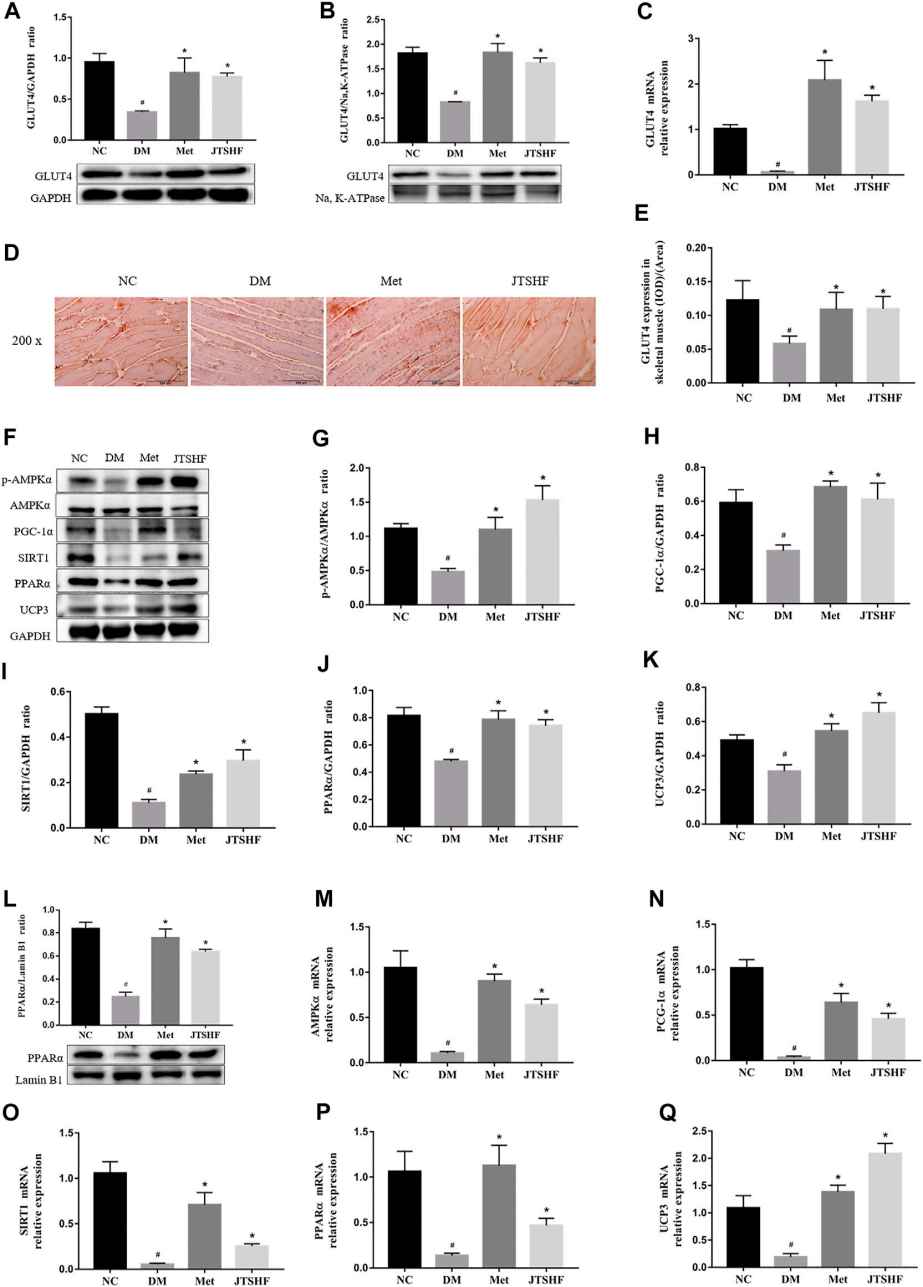
JTSHF promoted GLUT4 translocation by regulating AMPKα/SIRT1/PGC1α signaling pathway in skeletal muscles of HFD-STZ induced diabetic mice. **(A)** and **(B)** Relative expression of protein GLUT4 (distributed in cytoplasm and membrane) and **(C)** gene expression of GLUT4. **(D)** and **(E)** Immunohistochemical results and mean density of GLUT4 (IOD/Area). **(F–L)** Proteins expressions of AMPKα, p-AMPKα, PGC-1α, SIRT1, PPARα (distributed in cytoplasm and nucleus), and UCP3. **(M–Q)** Genes expressions of AMPKα, PGC-1α, SIRT1, PPARα, and UCP3. All Values were expressed as mean ± SEM. ^#^ *p* < 0.05 versus NC group, ^*^ *p* < 0.05 versus DM group; *n* = 3 for each group in A,B, G–L; *n* = 6 for each group in E; *n* = 4–5 for each group in C, M–Q.

In order to explore the potential mechanism of this herbal formula, we investigated the expression and translocation of GLUT4 by RT-PCR, WB, and IHC assays, respectively. Results from Figures 5A–E showed a decreased expression and membrane translocation of GLUT4 in skeletal muscles of the DM group compared with the NC group (*p* < 0.05), but the changes were reversed by oral administration with metformin or JTSHF (*p* < 0.05).

To further investigate whether AMPKα/SIRT1/PGC-1α signaling pathway is involved in the mechanism of JTSHF facilitating GLUT4 membrane translocation, we probed into crucial proteins and genes expressions involved in this pathway. As shown in Figures 5F–L, the expressions of the following proteins, AMPKα, p-AMPKα, PGC-1α, SIRT1, PPARα (distributed in cytoplasm and nucleus), and UCP3 were downregulated significantly in the skeletal muscles of the DM group compared with the normal mice (*p* < 0.05). As expected, the metformin or JTSHF could upregulate these proteins expressions after 8 weeks of intervention. Similarly, the mRNA expressions of AMPKα, PGC-1α, SIRT1, PPARα, and UCP3 in the skeletal muscles of diabetic mice were apparently lower than those in the NC group (*p* < 0.05). On the contrary, the downward trend of these genes went into reverse direction in the Met and JTSHF groups (Figures 5M–Q, *p* < 0.05). These results suggest that JTSHF may regulate AMPKα/SIRT1/PGC-1α signaling pathway to stimulate GLUT4 translocation, which contribute to improving IR in the skeletal muscles of diabetic mice.

## 4 Discussion

DM has attracted worldwide attention owing to a rapidly increasing morbidity and mortality. In TCM clinical practice, DM was deemed as Xiao-Ke disease. However, this idea has altered because a vast majority of patients with T2DM were not found to have typical Xiao-Ke symptoms of polydipsia, hyperphagia, polyuria, and weight loss at the same time (Pang et al., 2015; Bai et al., 2019). Consequently, the therapy concept of Xiao-Ke disease does not completely suitable for clinical prevention and treatment of DM. With the expansion of diffusion and influence on TCM worldwide, many researchers have made an in-depth investigation in the treatment of DM with TCM, and various innovative theories merging the concept of integrated traditional and western medicine have been gradually springing up (Xiao and Luo, 2018; Bi et al., 2020; Dou et al., 2021; Li et al., 2021). JTSHF was conceived from the guidance in the theory of “Treating T2DM in the homology of liver, kidney, and spleen”, which has remarkable effect on improving the clinical symptoms of patients with diabetes (Gao et al., 2009). Our previous research has validated that JTSHF granules could improve the disorder of glucolipid metabolism in obese mice by activating PI3K/AKT signaling pathway in skeletal muscle (Bai et al., 2019), while this study demonstrated a novel mechanism of JTSHF granules in promoting GLUT4 membrane translocation through regulating energy metabolism-related pathways to ameliorate IR in the skeletal muscles of diabetic mice.

Numerous studies have proven that IR is a principal characteristic and contributory factor of T2DM, and obesity caused by long-term HFD may induce IR (Yan et al., 2018; Li et al., 2020). More than that, intraperitoneal injection of STZ would damage β-cells and accelerate the development of T2DM in mice. Weight gain or loss after STZ injection has been reported in the HFD-STZ models by many researchers (Chen et al., 2019; Liu et al., 2019). In our study, the body weight and calorie intake in mice induced by HFD-STZ were higher than that in the normal mice, but little apparent weight gain was observed among diabetic mice during the whole intervention period, and there was no significant difference in body weight between the NC and DM groups at the late administration. Interestingly, JTSHF granules helped to control the calorie intake in diabetic mice, but slightly lowered the body weight of diabetic mice. However, we found that the body fat mass of mice in the JTSHF group was 5.29% lower than that of mice in the DM group, while lean body mass was 6.75% higher than that of mice in the DM group, which indicated that JTSHF granules tended to change body composition instead of losing weight. Moreover, some researchers supposed that high-dose STZ may severely damage the function of pancreatic β-cells and produce a decrease in insulin secretion (Glastras et al., 2016), which was similar to our findings that the diabetic model may not show obvious hyperinsulinemia. Although JTSHF showed little impact on serum insulin level either, it could improve insulin sensitivity in peripheral tissues. Additionally, we were surprised to notice that JTSHF granules displayed a good therapeutic effect in reducing the amount of water intake in diabetic mice, which was not investigated in earlier research. It has been proved that DM has a relationship with polydipsia (Westerberg, 2013; Saleh et al., 2015), and the potential reason for this alteration may be connected to the disorder of blood glucose regulation. For instance, with increasing blood glucose concentration, glucose is underutilized so as to form osmotic diuresis, bringing about dehydration of cells, and thereupon symptoms of dry mouth and excessive drinking are usually seen in diabetic patients (Westerberg, 2013; Saleh et al., 2015). Therefore, we could believe that the improvement of polydipsia by JTSHF may be related to the efficacy of lowering blood glucose.

Earlier studies showed that hyperlipidemia is considered a hazard factor for T2DM, and lipid metabolism disturbance may block insulin secretion and lead to impaired insulin sensitivity (Bai et al., 2015). In our experiment, the symptoms of persistent hyperglycemia, severe impaired glucose tolerance, and dyslipidemia were observed in T2DM mice induced by HFD-STZ. And treating with JTSHF greatly declined the serum lipid contents of NEFA, TC, TG, and LDL-C, but could hardly boost the serum level of HDL-C, which agree with our previous research (Bai et al., 2019). Besides, we found that JTSHF could improve IR in skeletal muscle, which may relate to its function on altering serum lipid profiles. The skeletal muscle is known to be rich in mitochondria and essential for glucose and lipid consumption *in vivo* (Richter-Stretton et al., 2020). Researches have revealed that obesity or excessive diet will ascend plasma free fatty acid (FFA) which proceeds with β-oxidation in the mitochondria of skeletal muscle, and maintains energy supply to skeletal muscle (Jung et al., 2018; Nishi et al., 2019; Richter-Stretton et al., 2020). When FFA content exceeds the metabolic need, it will give rise to incomplete fatty acid oxidation (FAO) and increase of reactive oxygen species (ROS), weaken the mitochondrial biogenesis, and further aggravate IR followed by disordered glycolipid metabolism (Nishi et al., 2019; Chen et al., 2021a). Briefly, these results validated that the hypoglycemic and lipid-lowering of JTSHF may be achieved by affecting mitochondrial-related metabolism. Furthermore, the inflammation caused by hyperglycemia and lipid accumulation may also lead to skeletal muscle injury and dysfunction (Richter-Stretton et al., 2020), and the intervention of JTSHF could alleviate the pathological changes in skeletal muscle and show a downward trend of inflammatory infiltration.

**FIGURE 6:**
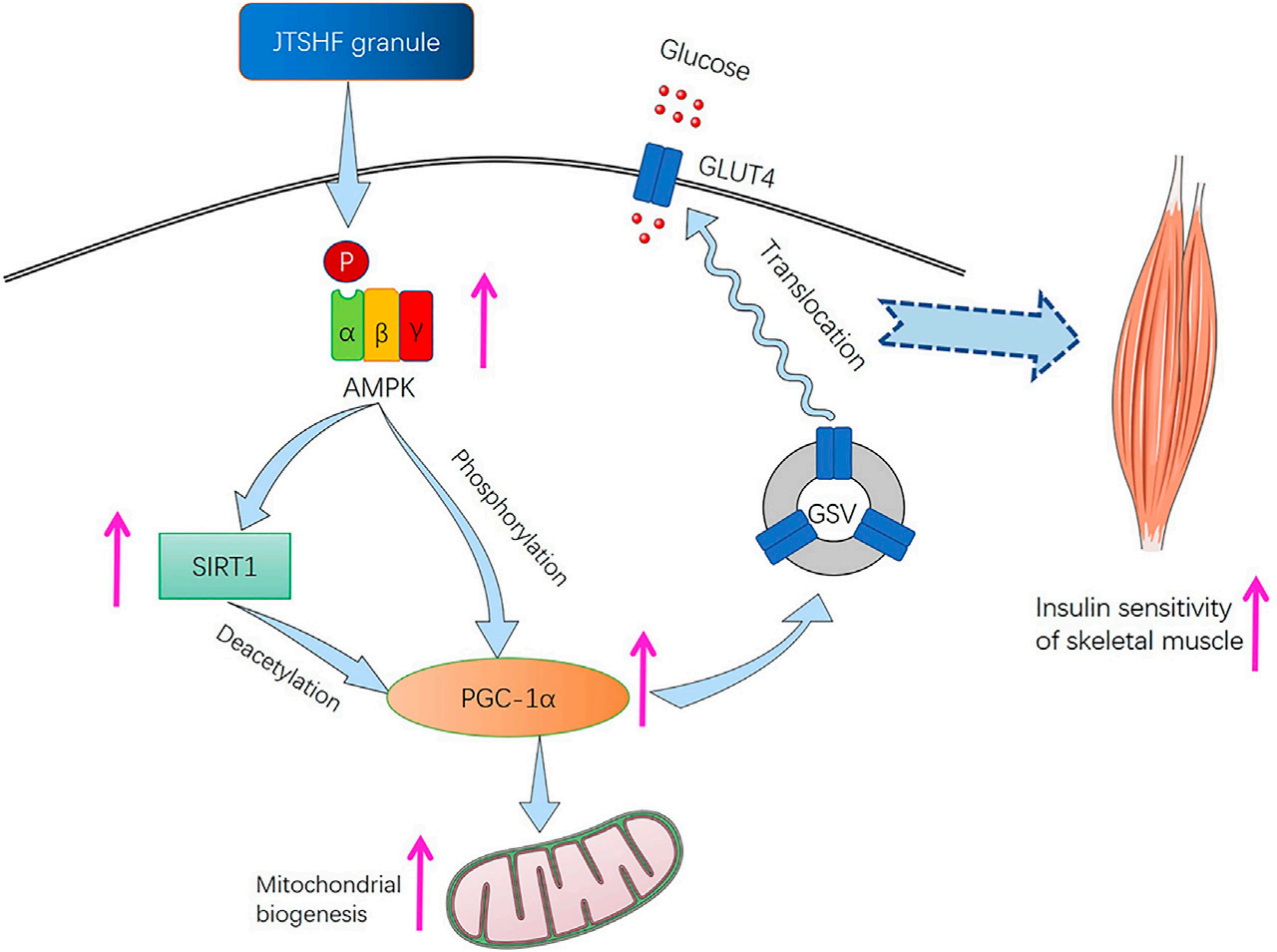
JTSHF granules ameliorated skeletal muscle IR via stimulating AMPKα/SIRT1/PGC-1α signaling pathway to modulate GLUT4 translocation.

The development of obesity, diabetes, and metabolic syndrome tends to cause a decline of mitochondrial mass in skeletal muscle. As previously described, the incomplete FAO and damaged insulin sensitivity in skeletal muscle has a certain causal relationship with insufficient quantity and function of mitochondria. Then the mitochondrial dysfunction, in turn, is chiefly responsible for the development of IR, T2DM, and other related complications (Yazıcı and Sezer, 2017; Xu et al., 2019a). Some evidence suggests that the impaired mitochondria may induce oxidative stress via increasing lipid peroxidation, and the latter may conversely alter mitochondrial biosynthesis and proteins activity participating in oxidative phosphorylation (Rains and Jain, 2011; Richter-Stretton et al., 2020). Therefore, the above process will affect the production of ATP, and then hinder the translocation of insulin-dependent GLUT4, which may eventually aggravate skeletal muscle IR. In this study, we demonstrated that JTSHF granules may enhance the expression and translocation of GLUT4 through upregulating AMPKα, PGC-1α, SIRT1, PPARα, and UCP3 proteins and genes, which were related to energy metabolism. AMPK is proven to be a vital regulator of glucose uptake, fatty acid oxidation, and mitochondrial biogenesis (Song et al., 2020; Xu et al., 2020), activated by the phosphorylation of Thr172 on AMPK autocatalytic subunit (α) with the rising of AMP-to-ATP ratio (Yano et al., 2020). Meanwhile, as an upstream regulator of PGC-1α in skeletal muscle, AMPK can also directly phosphorylate Thr177 and Ser538 on PGC-1α (Zhang et al., 2013). PGC-1α, a transcription factor coactivator, is recognized as a target for lowering the risk of IR and metabolic syndrome due to mitochondrial dysfunction (Williams and Gurd, 2012; Singh et al., 2020). Studies have shown that the expression of PGC-1α in skeletal muscles of diabetic mice was decreased, and *in vivo* study showed that the ectopic expression of PGC-1α remediated the expression level of GLUT4 (Zhang et al., 2013), which demonstrate that PGC-1α may have a beneficial role in the development of IR. SIRT1 is one of the most extensively described sirtuins. Its deacetylation of PGC-1α has kept being the research emphasis on energy metabolism and mitochondrial biogenesis of cells (Tang, 2016; Shen et al., 2022). Notably, SIRT1 also belongs to the downstream target of AMPK, because the deacetylation of SIRT1 is dependent on nicotinamide adenine dinucleotide (NAD^+^), which is enhanced by AMPK. In addition, AMPK signaling pathway could indirectly regulate PGC-1α by activating SIRT1 to facilitate the translocation of GLUT4 so as to show a marked increase in glucose utilization (Prasun, 2020; Entezari et al., 2022). In current study, we found that together with the enhanced translation of GLUT4, JTSHF increased mRNA and protein expressions of AMPKα, p-AMPKα, PGC-1α, SIRT1 in the skeletal muscles of diabetic mice, which indicated that JTSHF granules may activate GLUT4 via regulating AMPKα/SIRT1/PGC-1α signaling pathway*.* Moreover, strands of evidence have already supported that PPARα, the member of the nuclear receptor superfamily of transcription factors (Manickam et al., 2020), enhances muscle insulin sensitivity and upregulates the activities involved in fatty acid catabolism and mitochondrial β-oxidation (Ross et al., 2013; Manickam et al., 2020). As a common transcriptional coactivator to PPARα, PGC-1α can bind with PPARα to execute key metabolic regulation in skeletal muscle, adipose tissue, and other crucial organs, and participate in regulating FAO related enzymes and mitochondrial biogenesis (Cheng et al., 2018; Kalliora et al., 2019). It has been reported that exercise can upregulate PPARα and PGC-1α expressions in skeletal muscle, and the synergy of the two come to increase the mitochondrial content and function in skeletal muscle, which is conducive to enhancing insulin sensitivity and reversing the adverse effects of IR and T2DM in skeletal muscle physiology (Bajpeyi et al., 2017). All in all, our results substantiate that JTSHF granules may increase the expression and translocation of GLUT4 via regulating AMPKα/SIRT1/PGC-1α signaling pathway so as to ameliorate IR in skeletal muscle (Figure 6).

In conclusion, our study demonstrates that JTSHF may ameliorate skeletal muscle IR in diabetes mice, and its underlying mechanism may be attributed to stimulate the expression and translocation of GLUT4 by activating the AMPKα/SIRT1/PGC-1α signaling pathway, which not only provides sound scientific evidence for clinical application of the formula, but also offers a new strategy for prevention and treatment of T2DM.

## Data Availability

The original contributions presented in the study are included in the article/Supplementary Material, further inquiries can be directed to the corresponding authors.

## References

[B1] Abdul-Ghani M. A. DeFronzo R. A. (2010). Pathogenesis of insulin resistance in skeletal muscle. J. Biomed. Biotechnol. 2010, 476279. 10.1155/2010/476279 20445742 PMC2860140

[B2] Bai J. Zheng S. Jiang D. Han T. Li Y. Zhang Y. (2015). Oxidative stress contributes to abnormal glucose metabolism and insulin sensitivity in two hyperlipidemia models. Int. J. Clin. Exp. Pathol. 8 (10), 13193–13200. 26722518 PMC4680463

[B3] Bai Y. Bao X. Zhao D. Zhu R. Li R. Tian Y. (2020). Mechanism study of Jiang Tang San Hao Formula improving glucolipid metabolism in diet-induced obese mice. China J. Traditional Chin. Med. Pharm. 35 (05), 2253–2258.

[B4] Bai Y. Zhao D. Zhu R. Liu C. Liu H. Qin Y. (2019). Effects of jiangtangsanhao formula on glucolipid metabolism and PI3K/AKT signaling pathway in obese mice. Prog. Mod. Biomed. 19 (16), 3039–3043. 10.13241/j.cnki.pmb.2019.16.007

[B5] Bai Y. Zuo J. Fang X. Ma R. Tian T. Mo F. (2021). Protective effect of jiang tang Xiao ke granules against skeletal muscle IR via activation of the AMPK/SIRT1/PGC-1α signaling pathway. Oxid. Med. Cell. Longev. 2021, 5566053. 10.1155/2021/5566053 34326919 PMC8277912

[B6] Bajpeyi S. Covington J. D. Taylor E. M. Stewart L. K. Galgani J. E. Henagan T. M. (2017). Skeletal muscle PGC1α -1 nucleosome position and -260 nt DNA methylation determine exercise response and prevent ectopic lipid accumulation in men. Endocrinology 158 (7), 2190–2199. 10.1210/en.2017-00051 28398573 PMC5505213

[B7] Bi T. Zhan L. Zhou W. Sui H. (2020). Effect of the ZiBuPiYin recipe on diabetes-associated cognitive decline in zucker diabetic fatty rats after chronic psychological stress. Front. Psychiatry 11, 272. 10.3389/fpsyt.2020.00272 32372981 PMC7186306

[B8] Carnagarin R. Dharmarajan A. M. Dass C. R. (2015). Molecular aspects of glucose homeostasis in skeletal muscle--A focus on the molecular mechanisms of insulin resistance. Mol. Cell. Endocrinol. 417, 52–62. 10.1016/j.mce.2015.09.004 26362689

[B9] Chen H. W. Yang M. Y. Hung T. W. Chang Y. C. Wang C. J. (2019). *Nelumbo nucifera* leaves extract attenuate the pathological progression of diabetic nephropathy in high-fat diet-fed and streptozotocin-induced diabetic rats. J. Food Drug Anal. 27 (3), 736–748. 10.1016/j.jfda.2018.12.009 31324289 PMC9307034

[B10] Chen J. Fang W.-X. Li S.-J. Xiao S.-X. Li H.-J. Situ Y.-L. (2021a). Protective effect of ginsenoside rd on lipopolysaccharide-induced acute lung injury through its anti-inflammatory and anti-oxidative activity. World J. Tradit. Chin. Med. 7 (3), 383–390. 10.4103/wjtcm.wjtcm_12_21

[B11] Chen M. Huang N. Liu J. Huang J. Shi J. Jin F. (2021b). Ampk: A bridge between diabetes mellitus and alzheimer's disease. Behav. Brain Res. 400, 113043. 10.1016/j.bbr.2020.113043 33307136

[B12] Cheng C. F. Ku H. C. Lin H. (2018). PGC-1α as a pivotal factor in lipid and metabolic regulation. Int. J. Mol. Sci. 19 (11), E3447. 10.3390/ijms19113447 30400212 PMC6274980

[B13] DeFronzo R. A. Tripathy D. (2009). Skeletal muscle insulin resistance is the primary defect in type 2 diabetes. Diabetes Care 32 (2), S157–S163. 10.2337/dc09-S302 19875544 PMC2811436

[B14] Distefano G. Goodpaster B. H. (2018). Effects of exercise and aging on skeletal muscle. Cold Spring Harb. Perspect. Med. 8 (3), a029785. 10.1101/cshperspect.a029785 28432116 PMC5830901

[B15] Dou Z. Xia Y. Zhang J. Li Y. Zhang Y. Zhao L. (2021). Syndrome differentiation and treatment regularity in traditional Chinese medicine for type 2 diabetes: A text mining analysis. Front. Endocrinol. 12, 728032. 10.3389/fendo.2021.728032 PMC8733618 35002950

[B16] Elhassan S. A. M. Candasamy M. Chan E. W. L. Bhattamisra S. K. (2018). Autophagy and GLUT4: The missing pieces. Diabetes Metab. Syndr. 12 (6), 1109–1116. 10.1016/j.dsx.2018.05.020 29843994

[B17] Entezari M. Hashemi D. Taheriazam A. Zabolian A. Mohammadi S. Fakhri F. (2022). AMPK signaling in diabetes mellitus, insulin resistance and diabetic complications: A pre-clinical and clinical investigation. Biomed. Pharmacother. 146, 112563. 10.1016/j.biopha.2021.112563 35062059

[B18] Fazakerley D. J. Koumanov F. Holman G. D. (2022). GLUT4 on the move. Biochem. J. 479 (3), 445–462. 10.1042/bcj20210073 35147164 PMC8883492

[B19] Gadallah S. H. Ghanem H. M. Abdel-Ghaffar A. Metwaly F. G. Hanafy L. K. Ahmed E. K. (2021). 4-Phenylbutyric acid and rapamycin improved diabetic status in high fat diet/streptozotocin-induced type 2 diabetes through activation of autophagy. Arch. Physiol. Biochem. 127 (3), 235–244. 10.1080/13813455.2019.1628069 31215250

[B20] Gao S. Gong Y. Ni Q. Luo Z. Zhao J. Gao Y. (2009). Clinical study on treatment of type 2 diabetes from aspects of liver, spleen and kidney. China J. Traditional Chin. Med. Pharm. 24 (08), 1007–1010.

[B21] Glastras S. J. Chen H. Teh R. McGrath R. T. Chen J. Pollock C. A. (2016). Mouse models of diabetes, obesity and related kidney disease. PLoS One 11 (8), e0162131. 10.1371/journal.pone.0162131 27579698 PMC5006968

[B22] Guo W. L. Chen M. Pan W. L. Zhang Q. Xu J. X. Lin Y. C. (2020). Hypoglycemic and hypolipidemic mechanism of organic chromium derived from chelation of Grifola frondosa polysaccharide-chromium (III) and its modulation of intestinal microflora in high fat-diet and STZ-induced diabetic mice. Int. J. Biol. Macromol. 145, 1208–1218. 10.1016/j.ijbiomac.2019.09.206 31726162

[B23] Guo X. Sun W. Luo G. Wu L. Xu G. Hou D. (2019). Panax notoginseng saponins alleviate skeletal muscle insulin resistance by regulating the IRS1-PI3K-AKT signaling pathway and GLUT4 expression. FEBS Open Bio 9 (5), 1008–1019. 10.1002/2211-5463.12635 PMC6487711 30945455

[B24] Hagiwara N. (2014). Genetic dissection of the physiological role of skeletal muscle in metabolic syndrome. New J. Sci. 2014, 1–21. 10.1155/2014/635146

[B25] Han Y. C. Tang S. Q. Liu Y. T. Li A. M. Zhan M. Yang M. (2021). AMPK agonist alleviate renal tubulointerstitial fibrosis via activating mitophagy in high fat and streptozotocin induced diabetic mice. Cell Death Dis. 12 (10), 925. 10.1038/s41419-021-04184-8 34628484 PMC8502176

[B26] Herman R. Kravos N. A. Jensterle M. Janež A. Dolžan V. (2022). Metformin and insulin resistance: A review of the underlying mechanisms behind changes in GLUT4-mediated glucose transport. Int. J. Mol. Sci. 23 (3), 1264. 10.3390/ijms23031264 35163187 PMC8836112

[B27] Herzig S. Shaw R. J. (2018). Ampk: Guardian of metabolism and mitochondrial homeostasis. Nat. Rev. Mol. Cell Biol. 19 (2), 121–135. 10.1038/nrm.2017.95 28974774 PMC5780224

[B28] Inaishi J. Saisho Y. (2020). Beta-cell mass in obesity and type 2 diabetes, and its relation to pancreas fat: A mini-review. Nutrients 12 (12), E3846. 10.3390/nu12123846 33339276 PMC7766247

[B29] Jung T. W. Lee S. H. Kim H. C. Bang J. S. Abd El-Aty A. M. Hacımüftüoğlu A. (2018). METRNL attenuates lipid-induced inflammation and insulin resistance via AMPK or PPARδ-dependent pathways in skeletal muscle of mice. Exp. Mol. Med. 50 (9), 122–211. 10.1038/s12276-018-0147-5 PMC6137187 30213948

[B30] Kalliora C. Kyriazis I. D. Oka S. I. Lieu M. J. Yue Y. Area-Gomez E. (2019). Dual peroxisome-proliferator-activated-receptor-α/γ activation inhibits SIRT1-PGC1α axis and causes cardiac dysfunction. JCI Insight 5 (17), 129556. 10.1172/jci.insight.129556 31393858 PMC6777908

[B31] Kong H. L. Wang J. P. Li Z. Q. Zhao S. M. Dong J. Zhang W. W. (2009). Anti-hypoxic effect of ginsenoside Rbl on neonatal rat cardiomyocytes is mediated through the specific activation of glucose transporter-4 *ex vivo* . Acta Pharmacol. Sin. 30 (4), 396–403. 10.1038/aps.2009.2 19305424 PMC4002281

[B32] Leclerc I. Rutter G. A. (2004). AMP-Activated protein kinase: A new beta-cell glucose sensor?: Regulation by amino acids and calcium ions. Diabetes 53 (3), S67–S74. 10.2337/diabetes.53.suppl_3.s67 15561925

[B33] Lee K. Jin H. Chei S. Oh H. J. Lee J. Y. Lee B. Y. (2020). Effect of dietary silk peptide on obesity, hyperglycemia, and skeletal muscle regeneration in high-fat diet-fed mice. Cells 9 (2), E377. 10.3390/cells9020377 32041272 PMC7072146

[B34] Lee Y. S. Lee D. Park G. S. Ko S. H. Park J. Lee Y. K. (2021). Lactobacillus plantarum HAC01 ameliorates type 2 diabetes in high-fat diet and streptozotocin-induced diabetic mice in association with modulating the gut microbiota. Food Funct. 12 (14), 6363–6373. 10.1039/d1fo00698c 34105563

[B35] Leng Y. P. Qiu N. Fang W. J. Zhang M. He Z. M. Xiong Y. (2014). Involvement of increased endogenous asymmetric dimethylarginine in the hepatic endoplasmic reticulum stress of type 2 diabetic rats. PLoS One 9 (2), e97125. 10.1371/journal.pone.0097125 24918756 PMC4053342

[B36] Li L. Chen B. Zhu R. Li R. Tian Y. Liu C. (2019). Fructus Ligustri Lucidi preserves bone quality through the regulation of gut microbiota diversity, oxidative stress, TMAO and Sirt6 levels in aging mice. Aging (Albany NY) 11 (21), 9348–9368. 10.18632/aging.102376 31715585 PMC6874471

[B37] Li Q. P. Dou Y. X. Huang Z. W. Chen H. B. Li Y. C. Chen J. N. (2021). Therapeutic effect of oxyberberine on obese non-alcoholic fatty liver disease rats. Phytomedicine. 85, 153550. 10.1016/j.phymed.2021.153550 33831691

[B38] Li T. Chang R. Zhang H. Du M. Mao X. (2020). Water extract of potentilla discolor Bunge improves hepatic glucose homeostasis by regulating gluconeogenesis and glycogen synthesis in high-fat diet and streptozotocin-induced type 2 diabetic mice. Front. Nutr. 7, 161. 10.3389/fnut.2020.00161 33043040 PMC7522508

[B39] Liu Y. Deng J. Fan D. (2019). Ginsenoside Rk3 ameliorates high-fat-diet/streptozocin induced type 2 diabetes mellitus in mice via the AMPK/Akt signaling pathway. Food Funct. 10 (5), 2538–2551. 10.1039/c9fo00095j 30993294

[B40] Lu J. Chen P. P. Zhang J. X. Li X. Q. Wang G. H. Yuan B. Y. (2021). GPR43 deficiency protects against podocyte insulin resistance in diabetic nephropathy through the restoration of AMPKα activity. Theranostics 11 (10), 4728–4742. 10.7150/thno.56598 33754024 PMC7978296

[B41] Manickam R. Duszka K. Wahli W. (2020). PPARs and microbiota in skeletal muscle health and wasting. Int. J. Mol. Sci. 21 (21), E8056. 10.3390/ijms21218056 33137899 PMC7662636

[B42] Meng Q. Qi X. Fu Y. Chen Q. Cheng P. Yu X. (2020). Flavonoids extracted from mulberry (Morus alba L.) leaf improve skeletal muscle mitochondrial function by activating AMPK in type 2 diabetes. J. Ethnopharmacol. 248, 112326. 10.1016/j.jep.2019.112326 31639486

[B43] Merz K. E. Thurmond D. C. (2020). Role of skeletal muscle in insulin resistance and glucose uptake. Compr. Physiol. 10 (3), 785–809. 10.1002/cphy.c190029 32940941 PMC8074531

[B44] Mi J. He W. Lv J. Zhuang K. Huang H. Quan S. (2019). Effect of berberine on the HPA-axis pathway and skeletal muscle GLUT4 in type 2 diabetes mellitus rats. Diabetes Metab. Syndr. Obes. 12, 1717–1725. 10.2147/dmso.S211188 31564939 PMC6731988

[B45] Miyamoto L. (2018). AMPK as a metabolic intersection between diet and physical exercise. Yakugaku Zasshi 138 (10), 1291–1296. 10.1248/yakushi.18-00091-6 30270274

[B46] Moghetti P. Bacchi E. Brangani C. Donà S. Negri C. (2016). Metabolic effects of exercise. Front. Horm. Res. 47, 44–57. 10.1159/000445156 27348753

[B47] Nishi H. Higashihara T. Inagi R. (2019). Lipotoxicity in kidney, heart, and skeletal muscle dysfunction. Nutrients 11 (7), E1664. 10.3390/nu11071664 31330812 PMC6682887

[B48] Pang B. Li Q. W. Qin Y. L. Dong G. T. Feng S. Wang J. (2020). Traditional Chinese medicine for diabetic retinopathy: A systematic review and meta-analysis. Med. Baltim. 99 (7), e19102. 10.1097/md.0000000000019102 PMC7035093 32049817

[B49] Pang B. Zhou Q. Zhao T. Y. He L. S. Guo J. Chen H. D. (2015). Innovative thoughts on treating diabetes from the perspective of traditional Chinese medicine. Evid. Based. Complement. Altern. Med. 2015, 905432. 10.1155/2015/905432 PMC4609429 26504482

[B50] Prasun P. (2020). Mitochondrial dysfunction in metabolic syndrome. Biochim. Biophys. Acta. Mol. Basis Dis. 1866 (10), 165838. 10.1016/j.bbadis.2020.165838 32428560

[B51] Pyun D. H. Kim T. J. Park S. Y. Lee H. J. Abd El-Aty A. M. Jeong J. H. (2021). Patchouli alcohol ameliorates skeletal muscle insulin resistance and NAFLD via AMPK/SIRT1-mediated suppression of inflammation. Mol. Cell. Endocrinol. 538, 111464. 10.1016/j.mce.2021.111464 34601002

[B52] Rains J. L. Jain S. K. (2011). Oxidative stress, insulin signaling, and diabetes. Free Radic. Biol. Med. 50 (5), 567–575. 10.1016/j.freeradbiomed.2010.12.006 21163346 PMC3557825

[B53] Rena G. Hardie D. G. Pearson E. R. (2017). The mechanisms of action of metformin. Diabetologia 60 (9), 1577–1585. 10.1007/s00125-017-4342-z 28776086 PMC5552828

[B54] Richter E. A. Hargreaves M. (2013). Exercise, GLUT4, and skeletal muscle glucose uptake. Physiol. Rev. 93 (3), 993–1017. 10.1152/physrev.00038.2012 23899560

[B55] Richter E. A. (2021). Is GLUT4 translocation the answer to exercise-stimulated muscle glucose uptake? Am. J. Physiol. Endocrinol. Metab. 320 (2), E240–e243. 10.1152/ajpendo.00503.2020 33166188 PMC8260367

[B56] Richter-Stretton G. L. Fenning A. S. Vella R. K. (2020). Skeletal muscle - a bystander or influencer of metabolic syndrome? Diabetes Metab. Syndr. 14 (5), 867–875. 10.1016/j.dsx.2020.06.006 32562864

[B57] Ross J. S. Hu W. Rosen B. Snider A. J. Obeid L. M. Cowart L. A. (2013). Sphingosine kinase 1 is regulated by peroxisome proliferator-activated receptor α in response to free fatty acids and is essential for skeletal muscle interleukin-6 production and signaling in diet-induced obesity. J. Biol. Chem. 288 (31), 22193–22206. 10.1074/jbc.M113.477786 23766515 PMC3829312

[B58] Saleh J. Figueiredo M. A. Cherubini K. Salum F. G. (2015). Salivary hypofunction: An update on aetiology, diagnosis and therapeutics. Arch. Oral Biol. 60 (2), 242–255. 10.1016/j.archoralbio.2014.10.004 25463902

[B59] Sampath Kumar A. Maiya A. G. Shastry B. A. Vaishali K. Ravishankar N. Hazari A. (2019). Exercise and insulin resistance in type 2 diabetes mellitus: A systematic review and meta-analysis. Ann. Phys. Rehabil. Med. 62 (2), 98–103. 10.1016/j.rehab.2018.11.001 30553010

[B60] Sanvee G. M. Panajatovic M. V. Bouitbir J. Krähenbühl S. (2019). Mechanisms of insulin resistance by simvastatin in C2C12 myotubes and in mouse skeletal muscle. Biochem. Pharmacol. 164, 23–33. 10.1016/j.bcp.2019.02.025 30796916

[B61] Shen S. H. Singh S. P. Raffaele M. Waldman M. Hochhauser E. Ospino J. (2022). Adipocyte-specific expression of PGC1α promotes adipocyte browning and alleviates obesity-induced metabolic dysfunction in an HO-1-Dependent fashion. Antioxidants (Basel) 11 (6), 1147. 10.3390/antiox11061147 35740043 PMC9220759

[B62] Shrestha M. M. Lim C. Y. Bi X. Robinson R. C. Han W. (2021). Tmod3 phosphorylation mediates AMPK-dependent GLUT4 plasma membrane insertion in myoblasts. Front. Endocrinol. 12, 653557. 10.3389/fendo.2021.653557 PMC8095187 33959097

[B63] Singh S. P. Greenberg M. Glick Y. Bellner L. Favero G. Rezzani R. (2020). Adipocyte specific HO-1 gene therapy is effective in antioxidant treatment of insulin resistance and vascular function in an obese mice model. Antioxidants (Basel) 9 (1), E40. 10.3390/antiox9010040 31906399 PMC7022335

[B64] Song K. Zhang Y. Ga Q. Bai Z. Ge R. L. (2020). Increased insulin sensitivity by high-altitude hypoxia in mice with high-fat diet-induced obesity is associated with activated AMPK signaling and subsequently enhanced mitochondrial biogenesis in skeletal muscles. Obes. Facts 13 (5), 455–472. 10.1159/000508112 32966981 PMC7670386

[B65] Sun H. Saeedi P. Karuranga S. Pinkepank M. Ogurtsova K. Duncan B. B. (2022). IDF Diabetes Atlas: Global, regional and country-level diabetes prevalence estimates for 2021 and projections for 2045. Diabetes Res. Clin. Pract. 183, 109119. 10.1016/j.diabres.2021.109119 34879977 PMC11057359

[B66] Tabandeh M. R. Hosseini S. A. Hosseini M. (2017). Ginsenoside Rb1 exerts antidiabetic action on C2C12 muscle cells by leptin receptor signaling pathway. J. Recept. Signal Transduct. Res. 37 (4), 370–378. 10.1080/10799893.2017.1286676 28554304

[B67] Tanase D. M. Gosav E. M. Neculae E. Costea C. F. Ciocoiu M. Hurjui L. L. (2020). Role of gut microbiota on onset and progression of microvascular complications of type 2 diabetes (T2DM). Nutrients 12 (12), E3719. 10.3390/nu12123719 33276482 PMC7760723

[B68] Tang B. L. (2016). Sirt1 and the mitochondria. Mol. Cells 39 (2), 87–95. 10.14348/molcells.2016.2318 26831453 PMC4757807

[B69] Thomas C. C. Philipson L. H. (2015). Update on diabetes classification. Med. Clin. North Am. 99 (1), 1–16. 10.1016/j.mcna.2014.08.015 25456640

[B70] Wang C. Yue F. Kuang S. (2017). Muscle histology characterization using H&E staining and muscle fiber type classification using immunofluorescence staining. Bio. Protoc. 7 (10), e2279. 10.21769/BioProtoc.2279 PMC5526362 28752107

[B71] Wang J. He Y. Yu D. Jin L. Gong X. Zhang B. (2020a). Perilla oil regulates intestinal microbiota and alleviates insulin resistance through the PI3K/AKT signaling pathway in type-2 diabetic KKAy mice. Food Chem. Toxicol. 135, 110965. 10.1016/j.fct.2019.110965 31743741

[B72] Wang J. Ma Q. Li Y. Li P. Wang M. Wang T. (2020b). Research progress on Traditional Chinese Medicine syndromes of diabetes mellitus. Biomed. Pharmacother. 121, 109565. 10.1016/j.biopha.2019.109565 31704615

[B73] Westerberg D. P. (2013). Diabetic ketoacidosis: Evaluation and treatment. Am. Fam. Physician 87 (5), 337–346. 23547550

[B74] Williams C. B. Gurd B. J. (2012). Skeletal muscle SIRT1 and the genetics of metabolic health: Therapeutic activation by pharmaceuticals and exercise. Appl. Clin. Genet. 5, 81–91. 10.2147/tacg.S31276 23776383 PMC3681195

[B75] Xiao E. Luo L. (2018). Alternative therapies for diabetes: A comparison of western and traditional Chinese medicine (TCM) approaches. Curr. Diabetes Rev. 14 (6), 487–496. 10.2174/1573399813666170519103230 28523995

[B76] Xu D. Jiang Z. Sun Z. Wang L. Zhao G. Hassan H. M. (2019a). Mitochondrial dysfunction and inhibition of myoblast differentiation in mice with high-fat-diet-induced pre-diabetes. J. Cell. Physiol. 234 (5), 7510–7523. 10.1002/jcp.27512 30362548

[B77] Xu D. Q. Li C. J. Jiang Z. Z. Wang L. Huang H. F. Li Z. J. (2020). The hypoglycemic mechanism of catalpol involves increased AMPK-mediated mitochondrial biogenesis. Acta Pharmacol. Sin. 41 (6), 791–799. 10.1038/s41401-019-0345-2 31937931 PMC7470840

[B78] Xu L. Li Y. Dai Y. Peng J. (2018). Natural products for the treatment of type 2 diabetes mellitus: Pharmacology and mechanisms. Pharmacol. Res. 130, 451–465. 10.1016/j.phrs.2018.01.015 29395440

[B79] Xu S. Bian R. Chen X. (2003). Experimental methodology of pharmacology. Beijing: People's Medical Publishing House.

[B80] Xu Y. X. Z. Xi S. Qian X. (2019b). Evaluating traditional Chinese medicine and herbal products for the treatment of gestational diabetes mellitus. J. Diabetes Res. 2019, 9182595. 10.1155/2019/9182595 31886289 PMC6915007

[B81] Yan J. Wang C. Jin Y. Meng Q. Liu Q. Liu Z. (2018). Catalpol ameliorates hepatic insulin resistance in type 2 diabetes through acting on AMPK/NOX4/PI3K/AKT pathway. Pharmacol. Res. 130, 466–480. 10.1016/j.phrs.2017.12.026 29284152

[B82] Yano N. Zhang L. Wei D. Dubielecka P. M. Wei L. Zhuang S. (2020). Irisin counteracts high glucose and fatty acid-induced cytotoxicity by preserving the AMPK-insulin receptor signaling axis in C2C12 myoblasts. Am. J. Physiol. Endocrinol. Metab. 318 (5), E791–e805. 10.1152/ajpendo.00219.2019 32182124 PMC7272726

[B83] Yazıcı D. Sezer H. (2017). Insulin resistance, obesity and lipotoxicity. Adv. Exp. Med. Biol. 960, 277–304. 10.1007/978-3-319-48382-5_12 28585204

[B84] YuY.ZhaoY.TengF.LiJ.GuanY.XuJ. (2018). Berberine improves cognitive deficiency and muscular dysfunction via activation of the AMPK/SIRT1/PGC-1a pathway in skeletal muscle from naturally aging rats. J. Nutr. Health Aging 22 (6), 710–717. 10.1007/s12603-018-1015-7 29806860

[B85] Zhang C. Deng J. Liu D. Tuo X. Xiao L. Lai B. (2018a). Nuciferine ameliorates hepatic steatosis in high-fat diet/streptozocin-induced diabetic mice through a PPARα/PPARγ coactivator-1α pathway. Br. J. Pharmacol. 175 (22), 4218–4228. 10.1111/bph.14482 30129056 PMC6193881

[B86] Zhang C. Jiang Y. Liu J. Jin M. Qin N. Chen Y. (2018b). AMPK/AS160 mediates tiliroside derivatives-stimulated GLUT4 translocation in muscle cells. Drug Des. devel. Ther. 12, 1581–1587. 10.2147/dddt.S164441 PMC5989705 29910604

[B87] Zhang L. N. Zhou H. Y. Fu Y. Y. Li Y. Y. Wu F. Gu M. (2013). Novel small-molecule PGC-1α transcriptional regulator with beneficial effects on diabetic db/db mice. Diabetes 62 (4), 1297–1307. 10.2337/db12-0703 23250358 PMC3609556

[B88] Zheng W. Wang G. Zhang Z. Wang Z. Ma K. (2020). Research progress on classical traditional Chinese medicine formula Liuwei Dihuang pills in the treatment of type 2 diabetes. Biomed. Pharmacother. 121, 109564. 10.1016/j.biopha.2019.109564 31683180

