# Supplementary figures and images for: Jiangtang Sanhao formula ameliorates skeletal muscle insulin resistance via regulating GLUT4 translocation in diabetic mice
Source: Front Pharmacol. 2022 Sep 8;13:950535. doi: 10.3389/fphar.2022.950535 (PMC9492927; doi:10.3389/fphar.2022.950535)

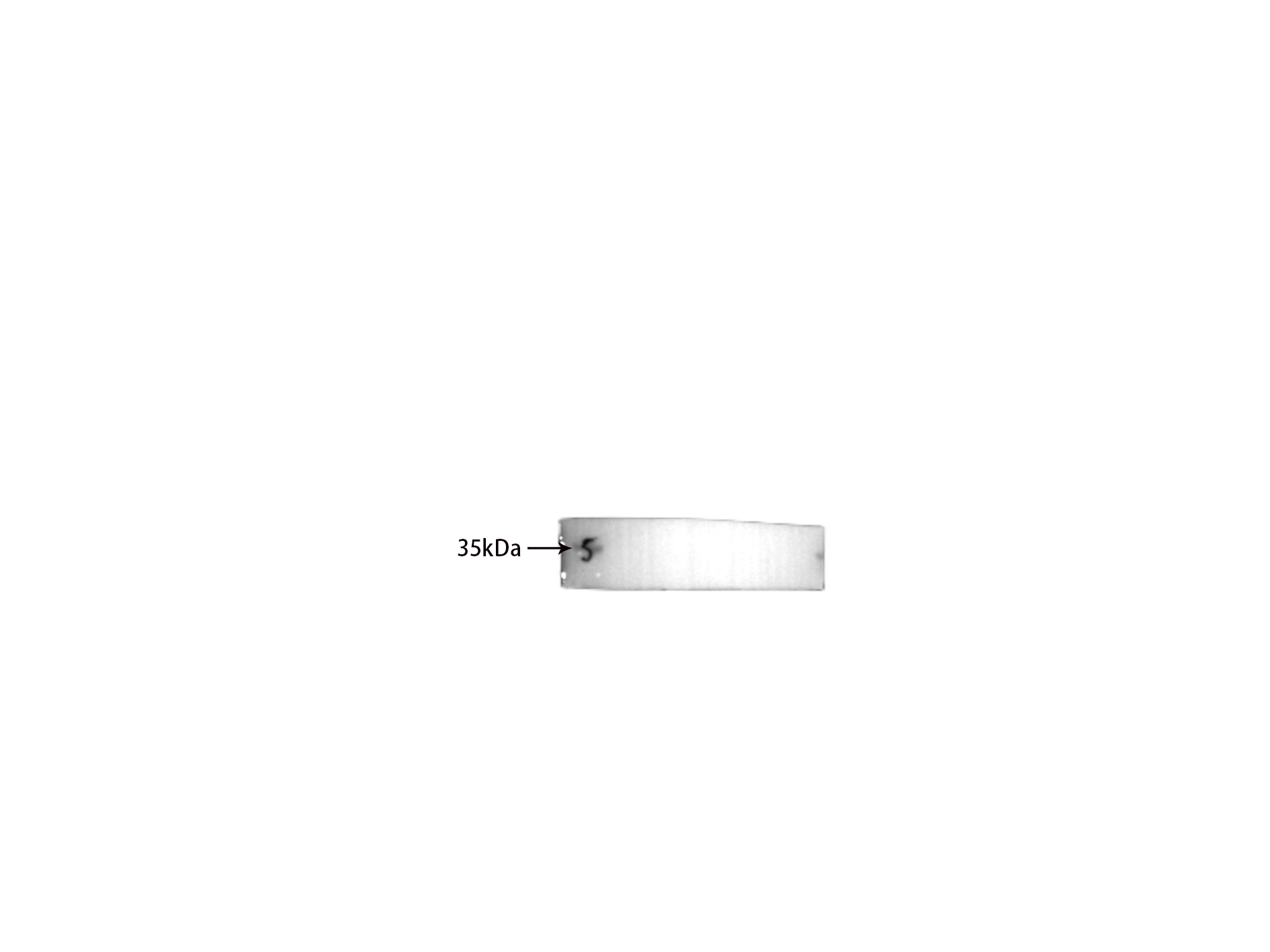

Supplement: Supplementary file 2 [file DataSheet1.ZIP › original WB photo/GAPDH/GAPDH-F-marker_pub.jpg]

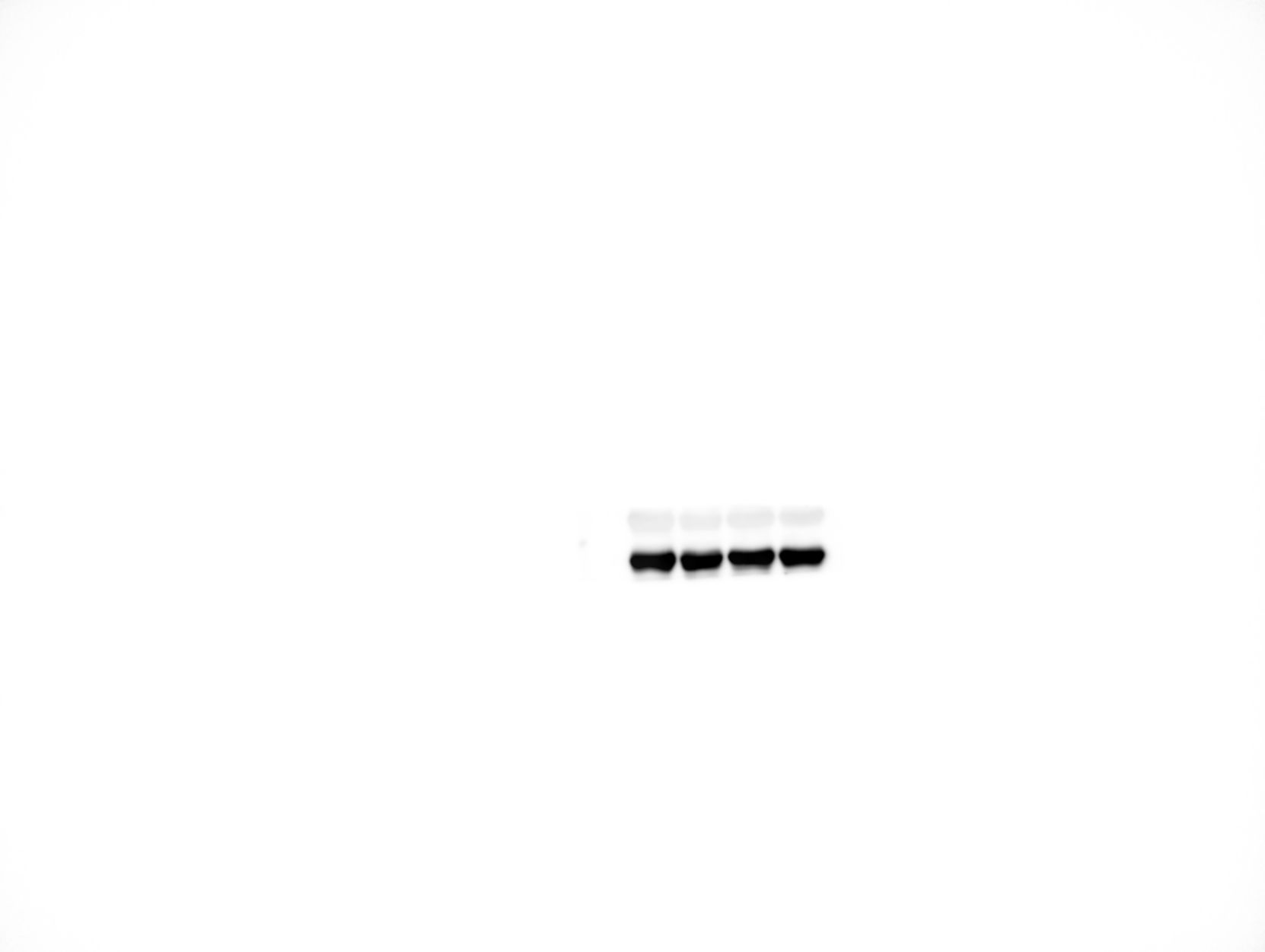

Supplement: Supplementary file 2 [file DataSheet1.ZIP › original WB photo/GAPDH/GAPDH-F_pub.jpg]

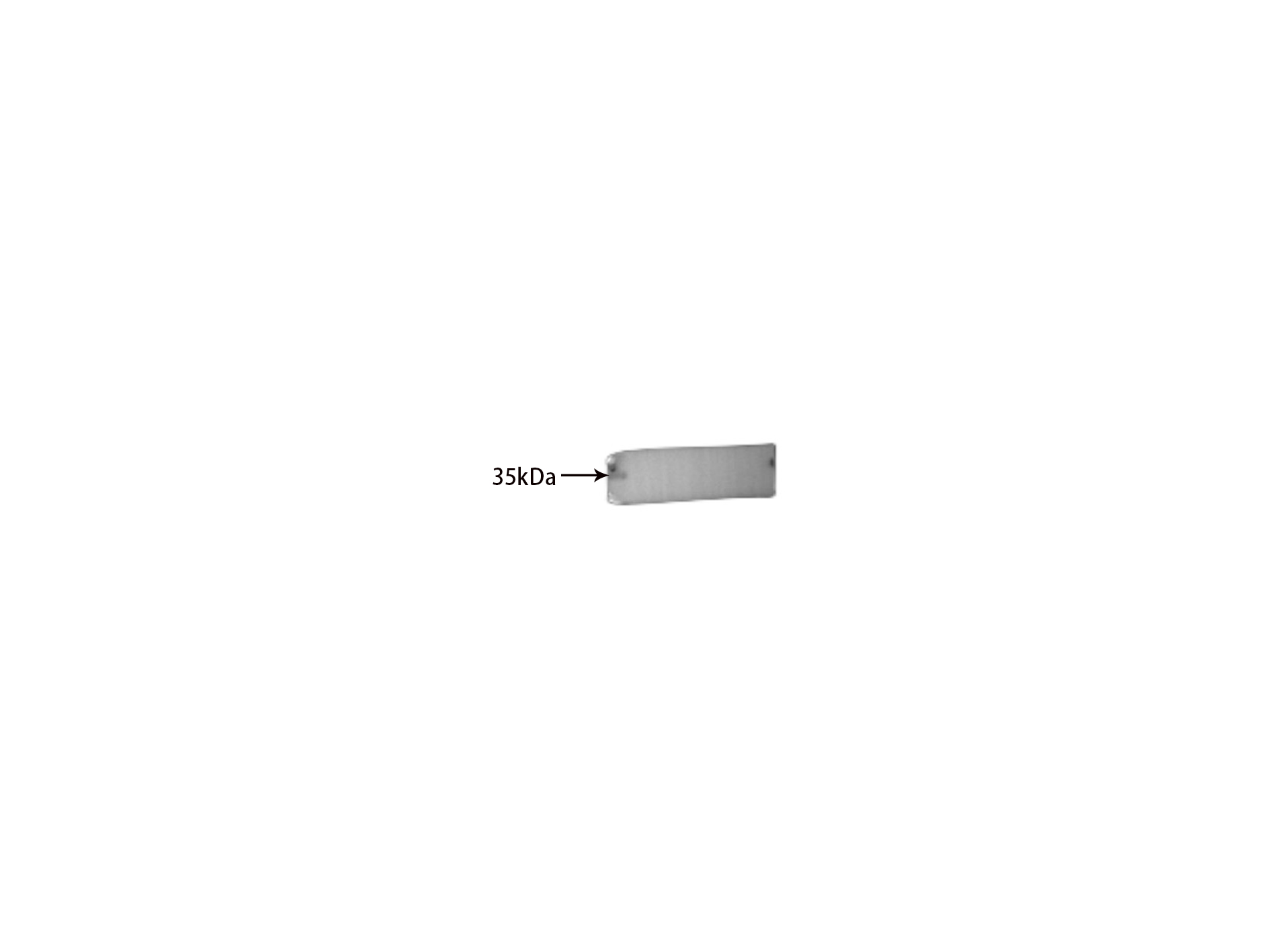

Supplement: Supplementary file 2 [file DataSheet1.ZIP › original WB photo/GAPDH/GAPDH-S-marker_pub.jpg]

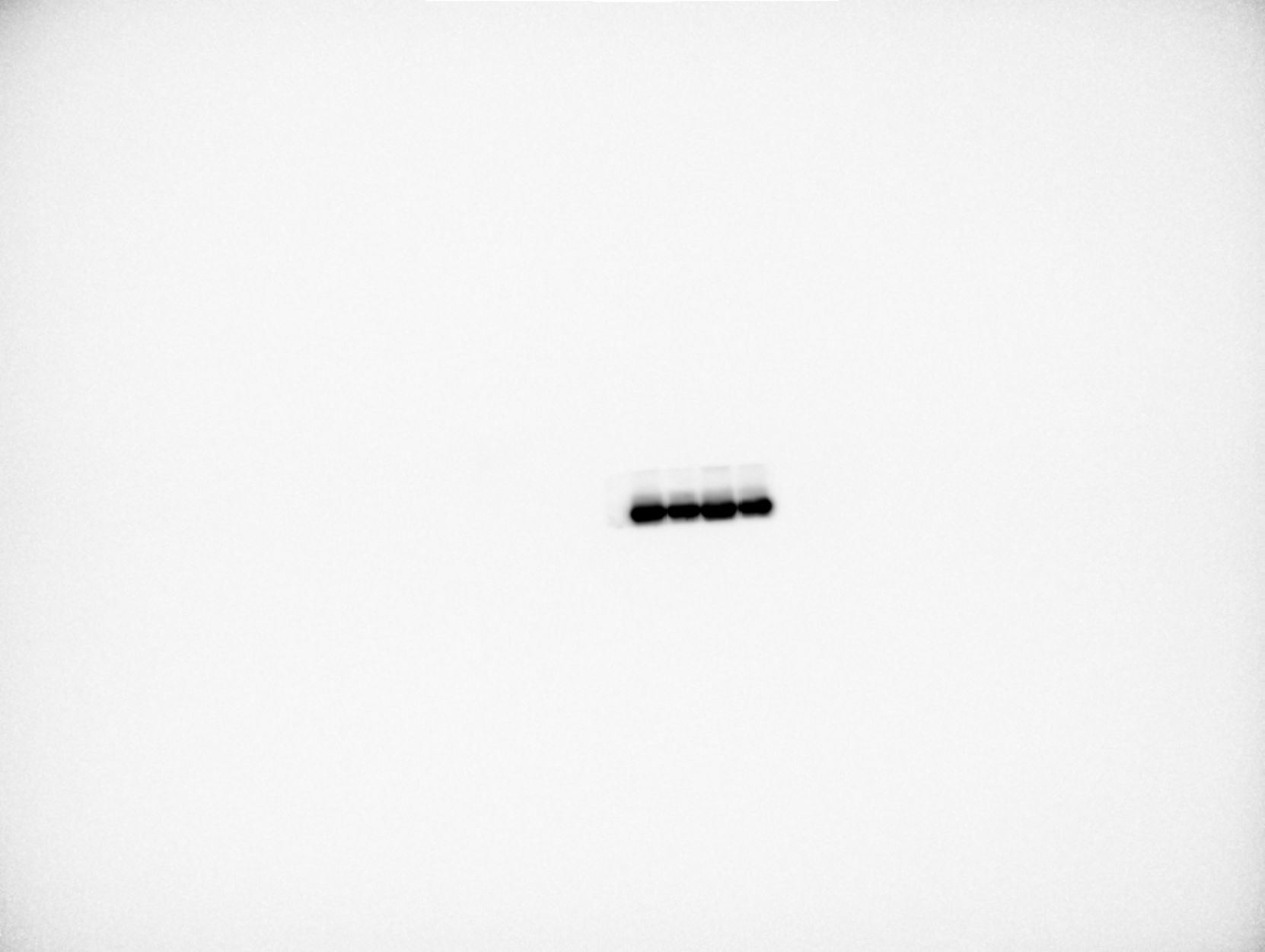

Supplement: Supplementary file 2 [file DataSheet1.ZIP › original WB photo/GAPDH/GAPDH-S_pub.jpg]

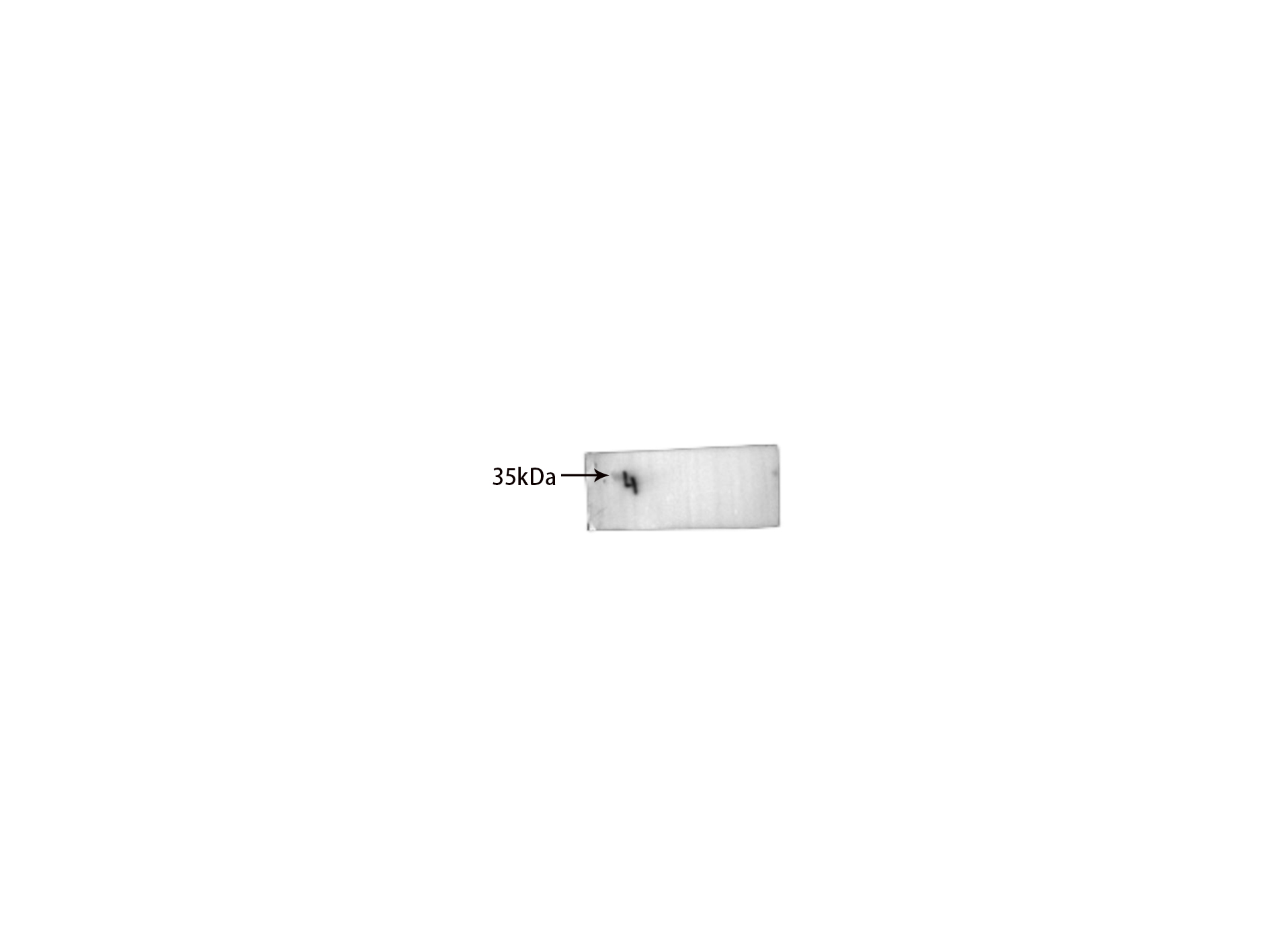

Supplement: Supplementary file 2 [file DataSheet1.ZIP › original WB photo/GAPDH/GAPDH-T-marker_pub.jpg]

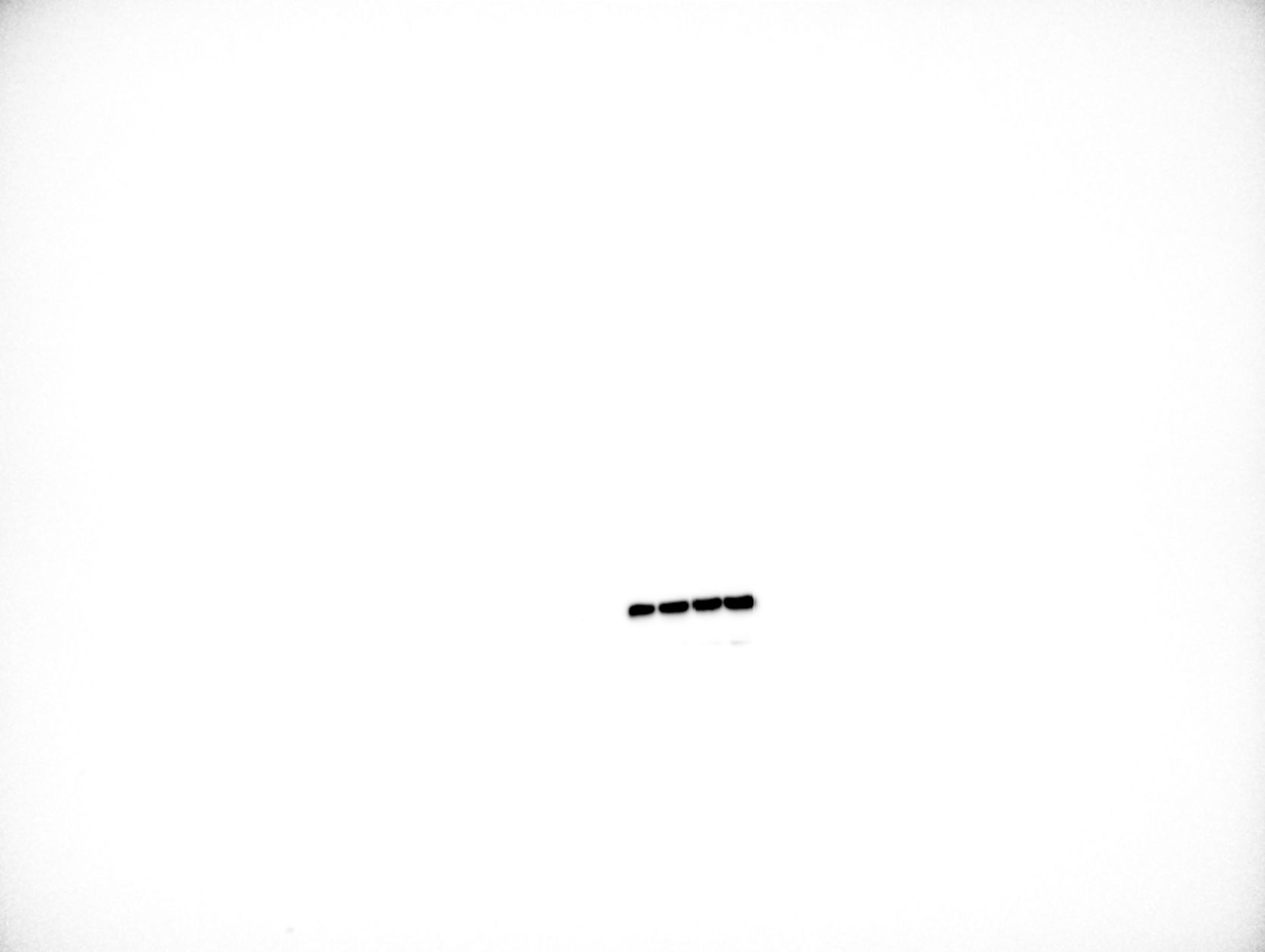

Supplement: Supplementary file 2 [file DataSheet1.ZIP › original WB photo/GAPDH/GAPDH-T_pub.jpg]

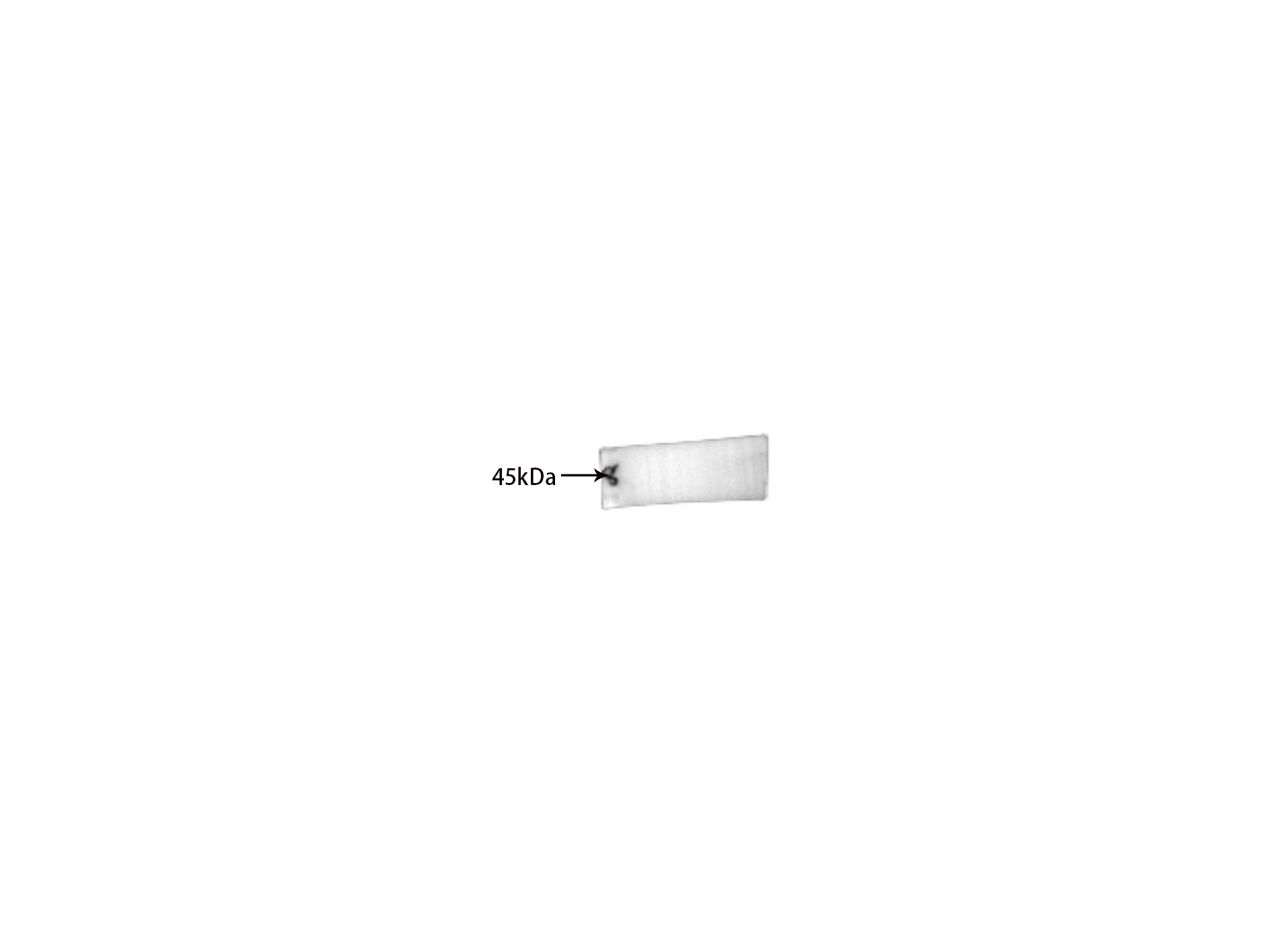

Supplement: Supplementary file 2 [file DataSheet1.ZIP › original WB photo/GLUT4/GLUT4-F-marker_pub.jpg]

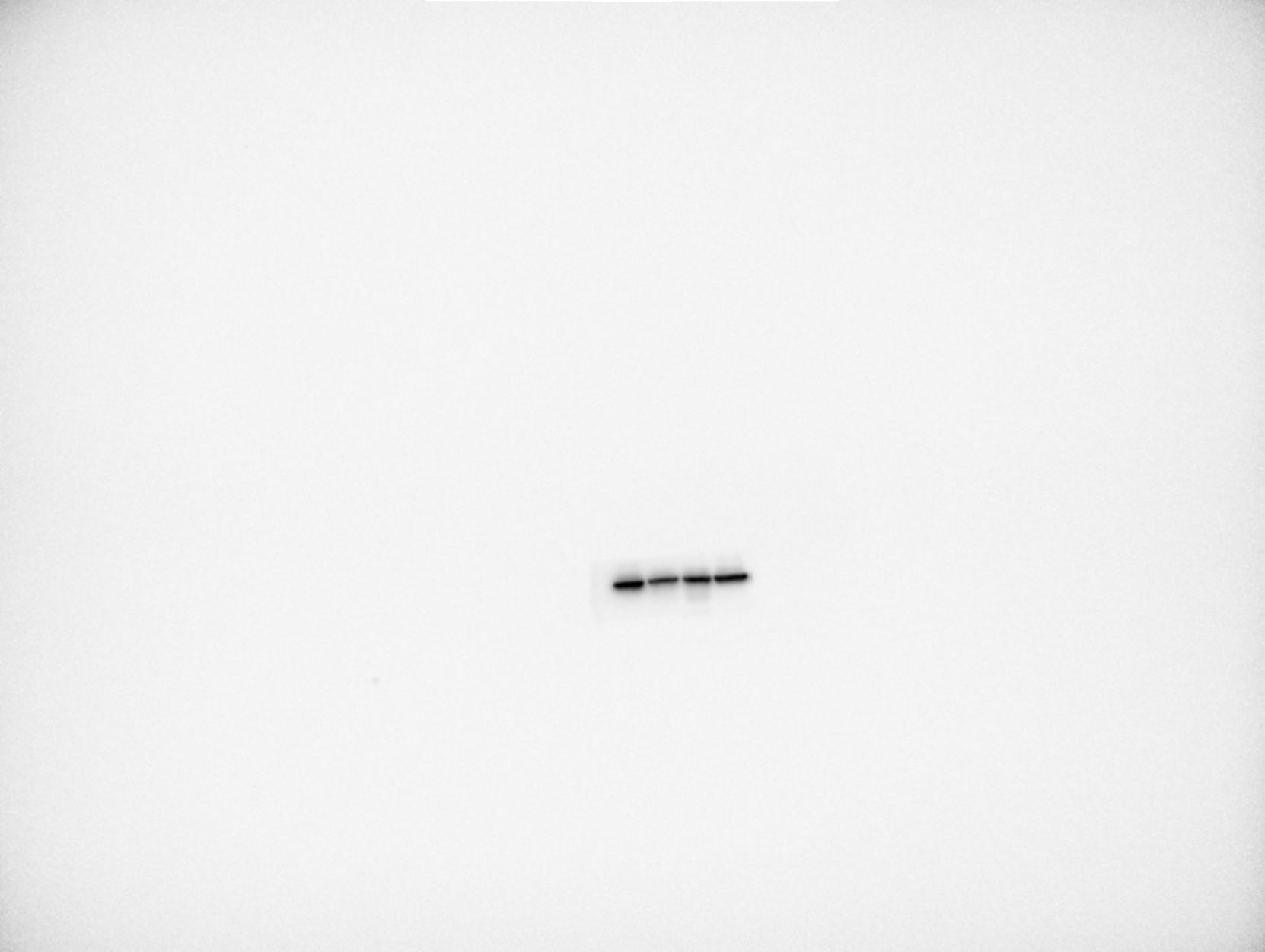

Supplement: Supplementary file 2 [file DataSheet1.ZIP › original WB photo/GLUT4/GLUT4-F_pub.jpg]

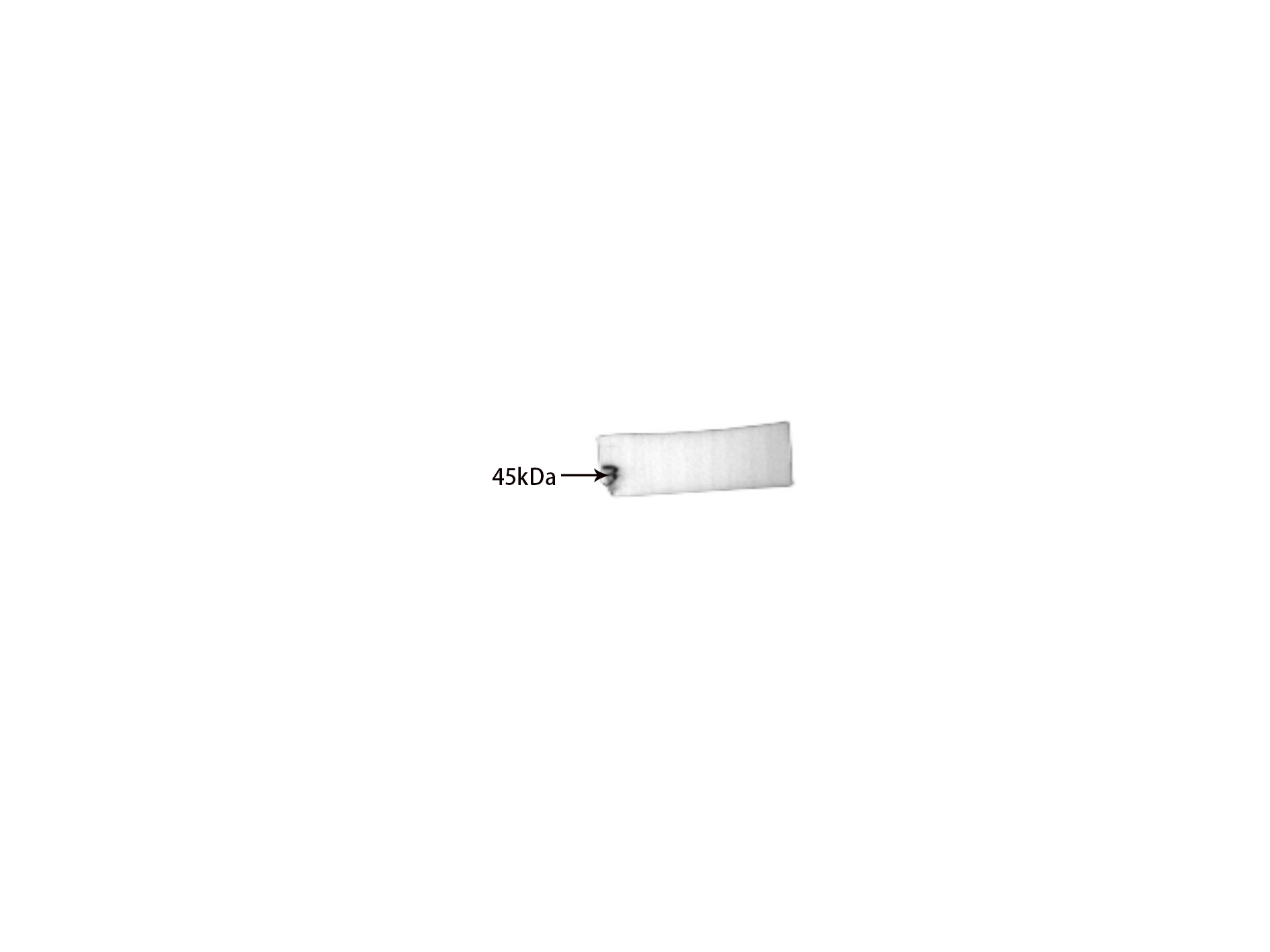

Supplement: Supplementary file 2 [file DataSheet1.ZIP › original WB photo/GLUT4/GLUT4-S-marker_pub.jpg]

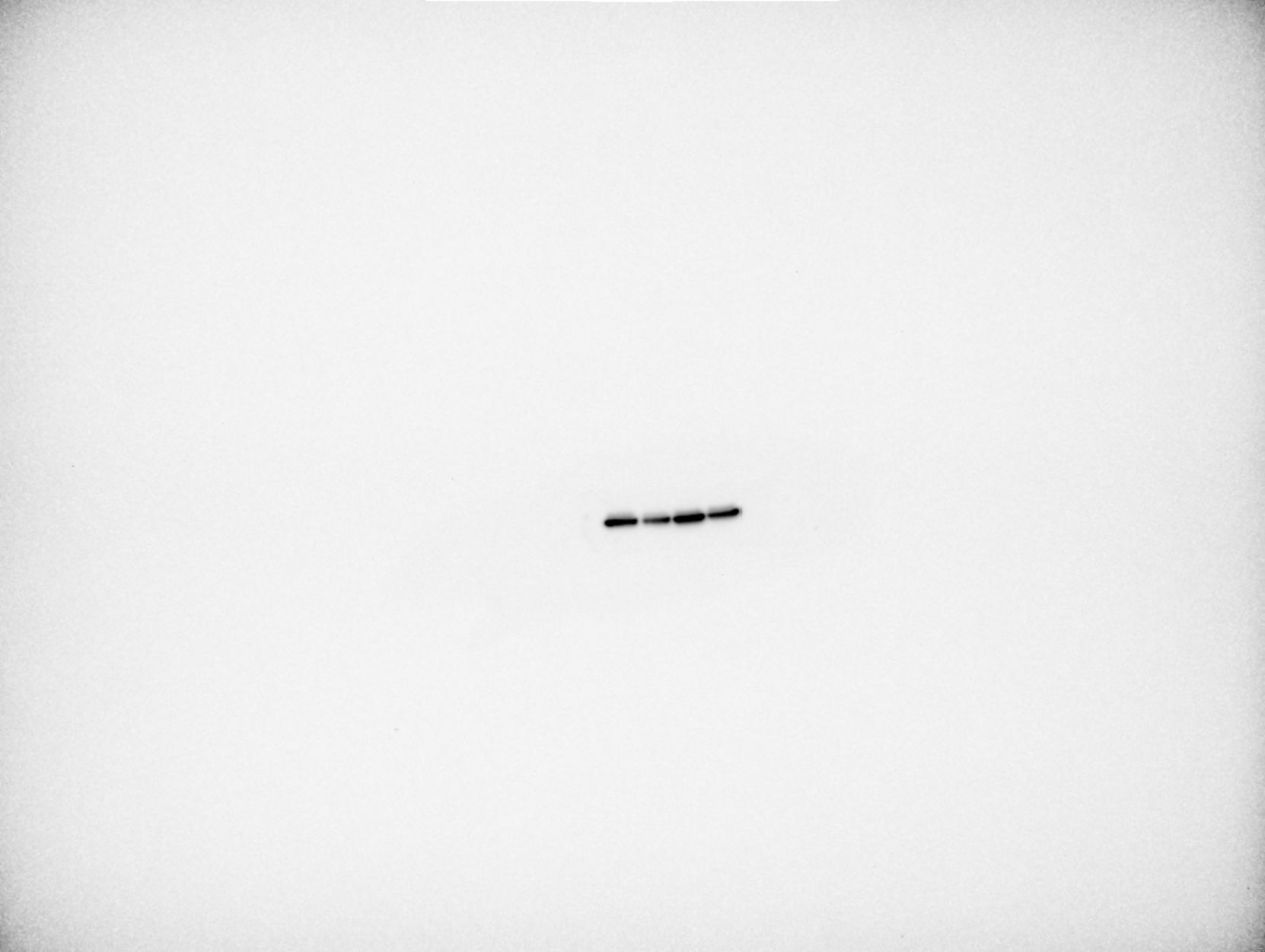

Supplement: Supplementary file 2 [file DataSheet1.ZIP › original WB photo/GLUT4/GLUT4-S_pub.jpg]

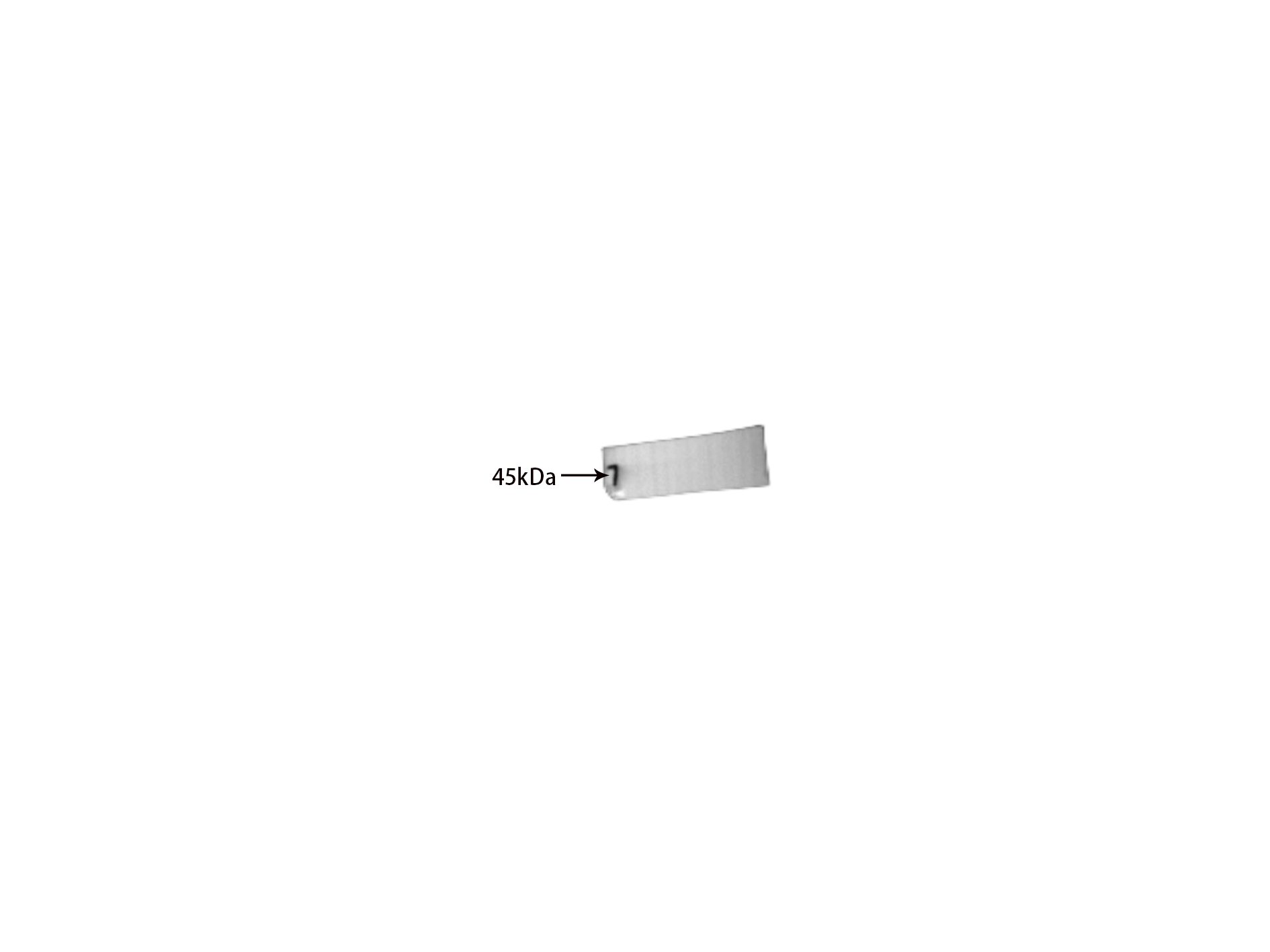

Supplement: Supplementary file 2 [file DataSheet1.ZIP › original WB photo/GLUT4/GLUT4-T-marker_pub.jpg]

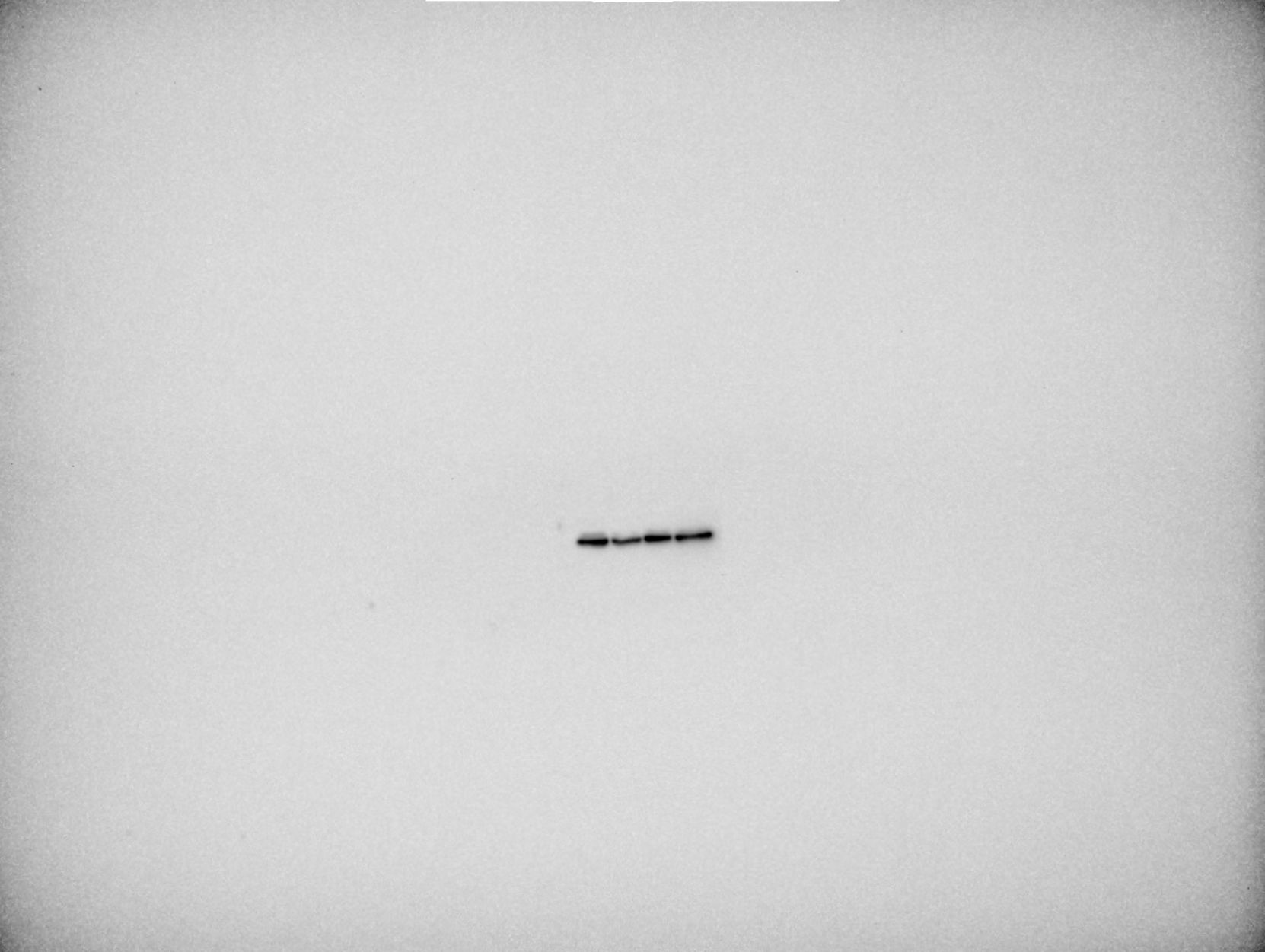

Supplement: Supplementary file 2 [file DataSheet1.ZIP › original WB photo/GLUT4/GLUT4-T_pub.jpg]

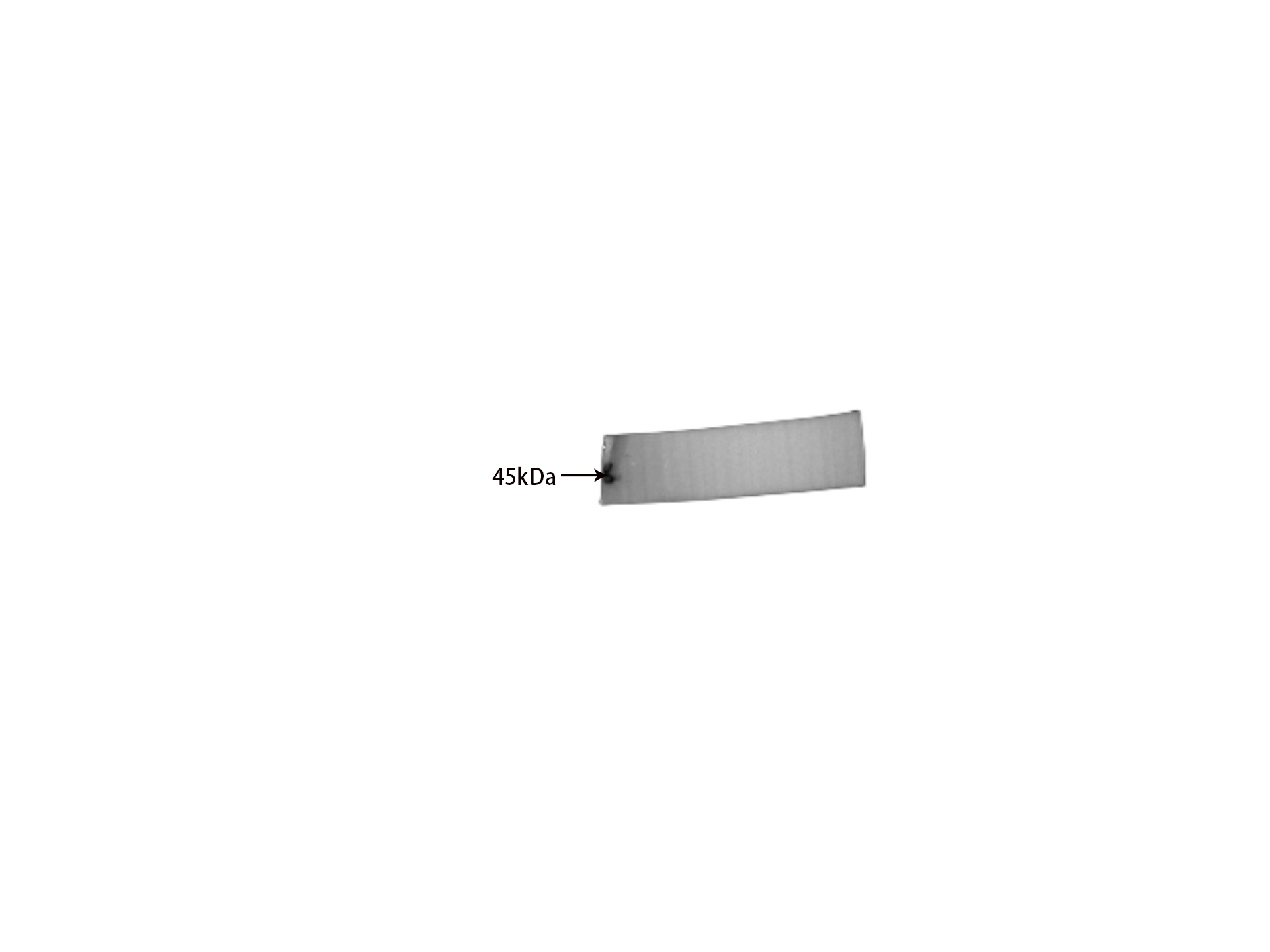

Supplement: Supplementary file 2 [file DataSheet1.ZIP › original WB photo/GLUT4-membrane/GLUT4-membrane-F-marker_pub.jpg]

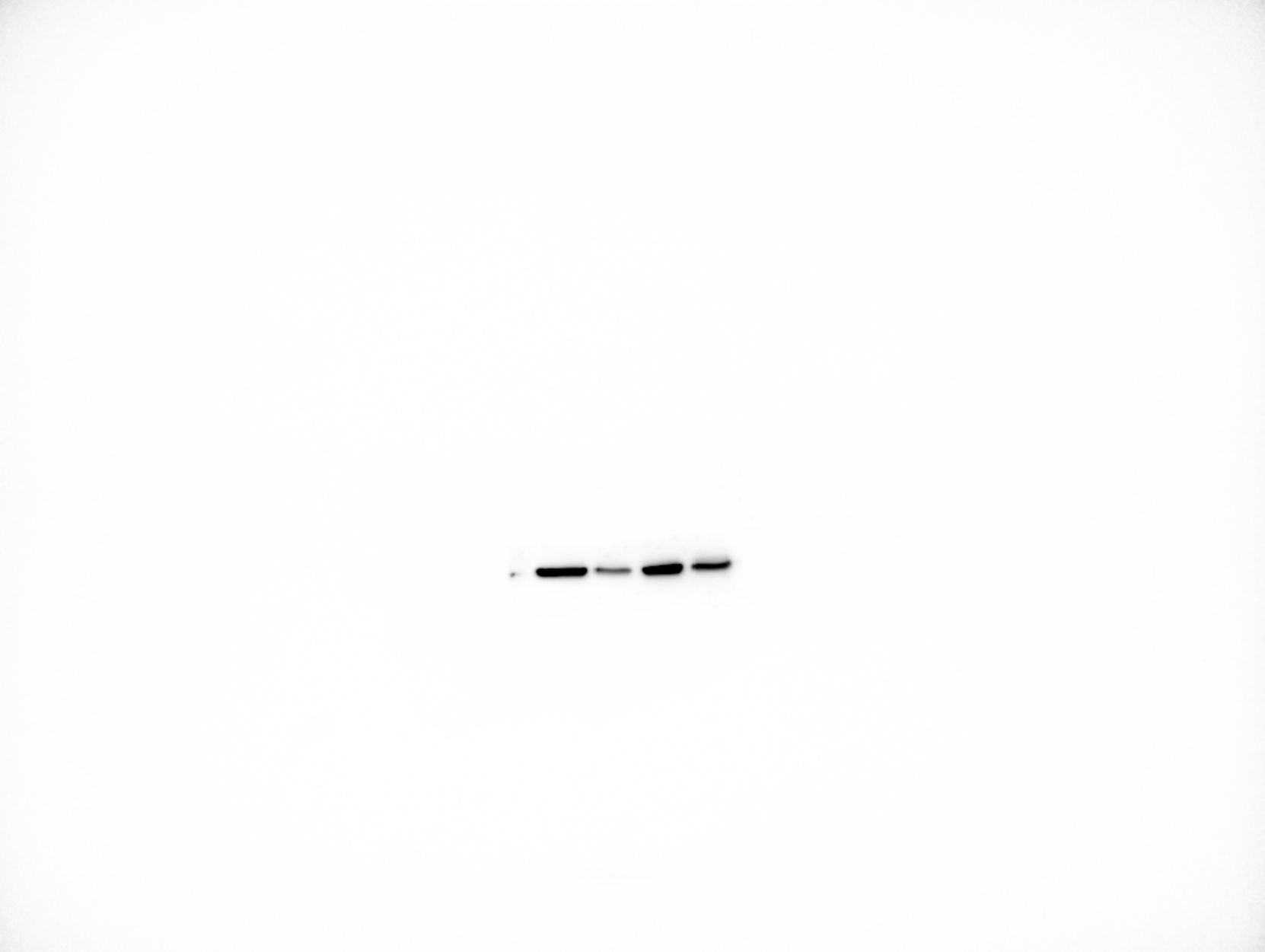

Supplement: Supplementary file 2 [file DataSheet1.ZIP › original WB photo/GLUT4-membrane/GLUT4-membrane-F_pub.jpg]

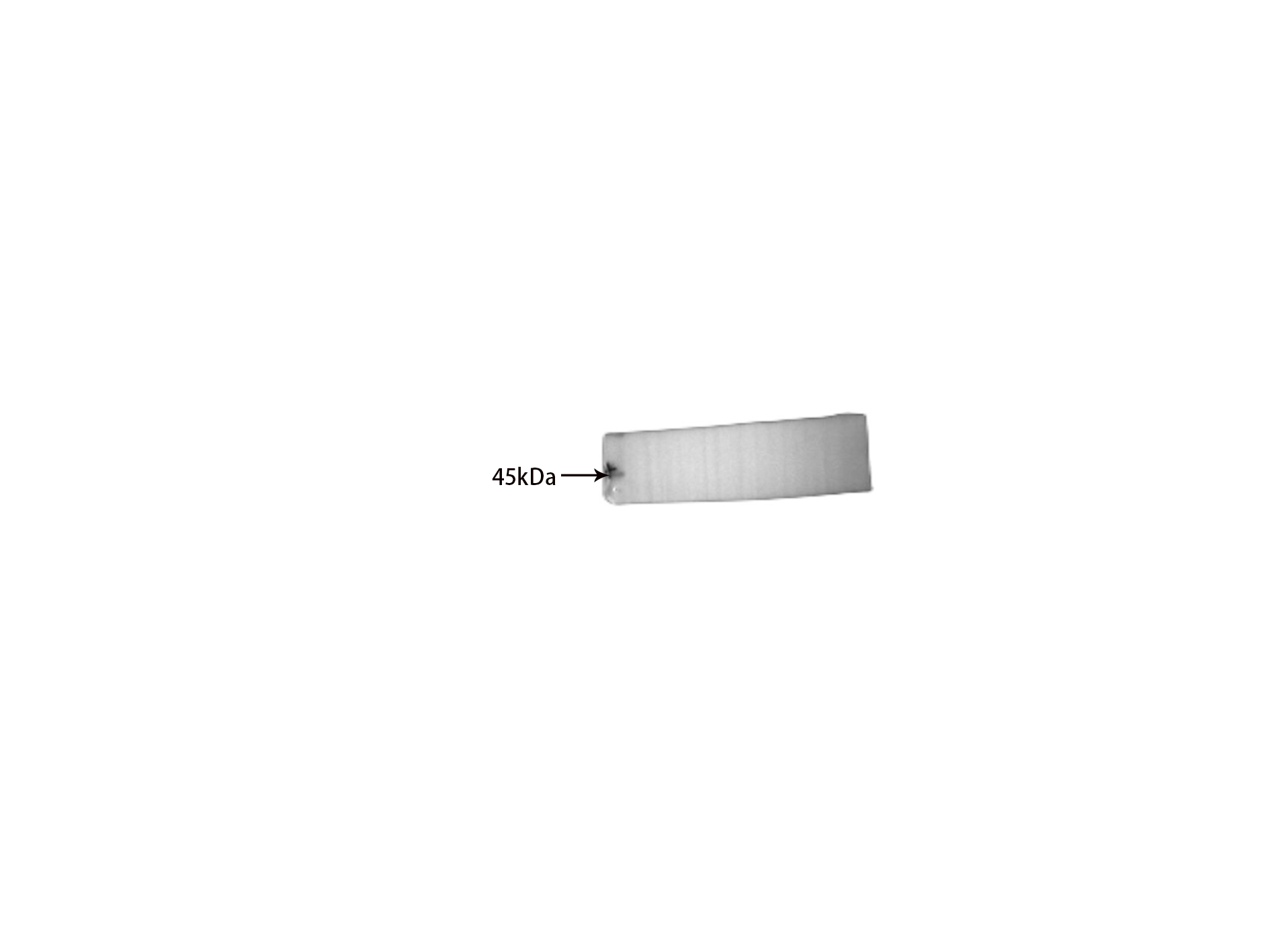

Supplement: Supplementary file 2 [file DataSheet1.ZIP › original WB photo/GLUT4-membrane/GLUT4-membrane-S-marker_pub.jpg]

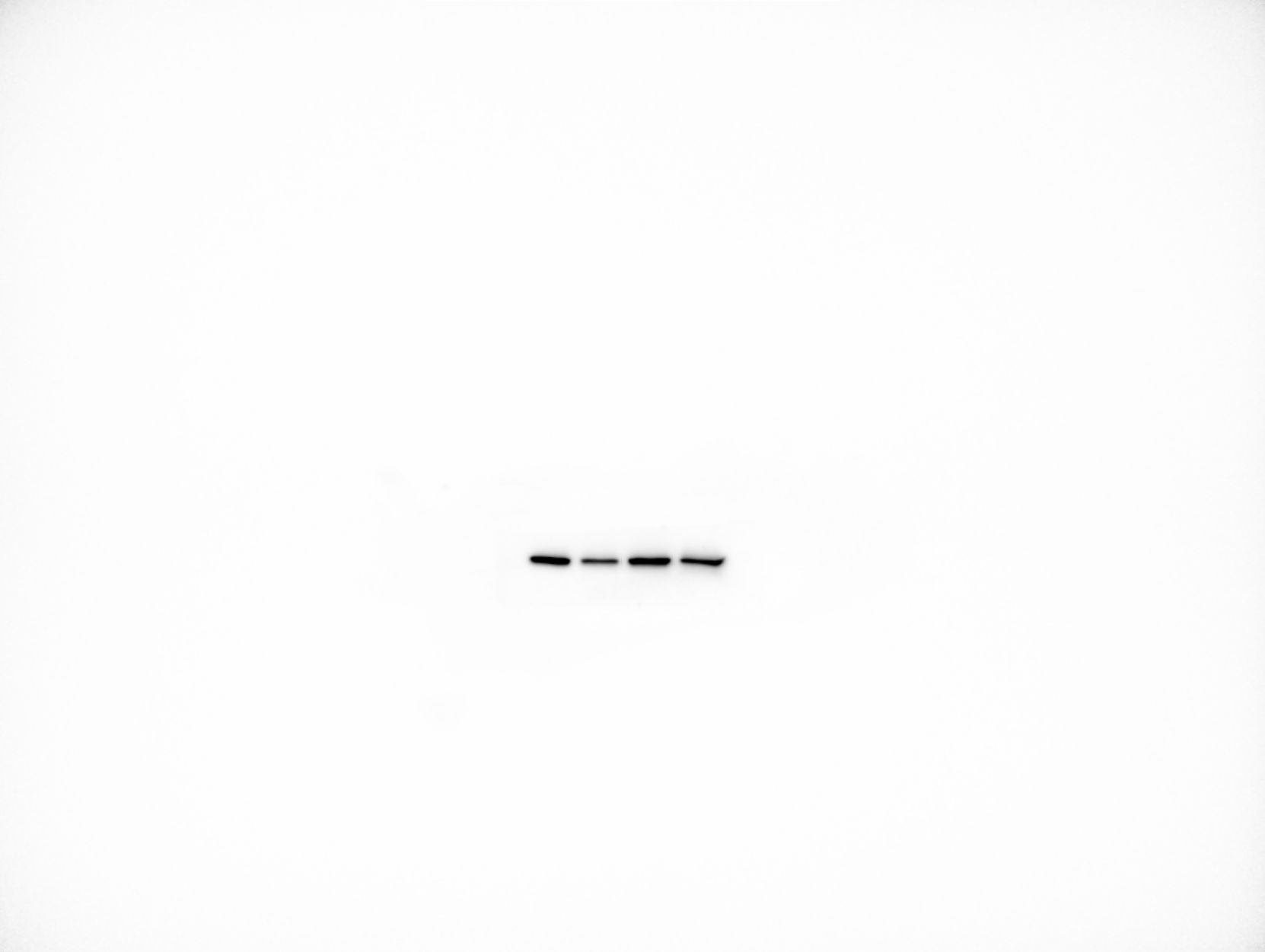

Supplement: Supplementary file 2 [file DataSheet1.ZIP › original WB photo/GLUT4-membrane/GLUT4-membrane-S_pub.jpg]

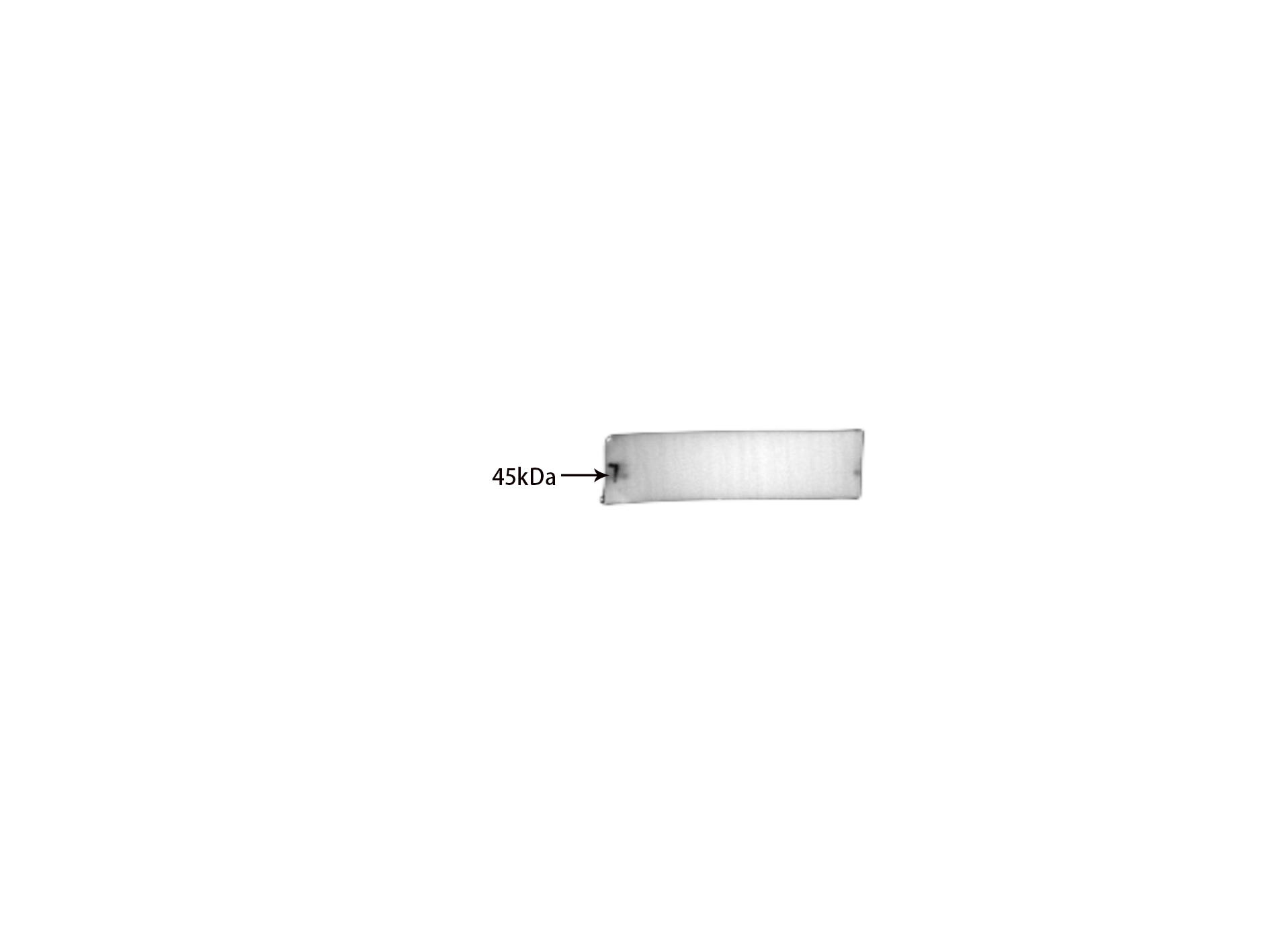

Supplement: Supplementary file 2 [file DataSheet1.ZIP › original WB photo/GLUT4-membrane/GLUT4-membrane-T-marker_pub.jpg]

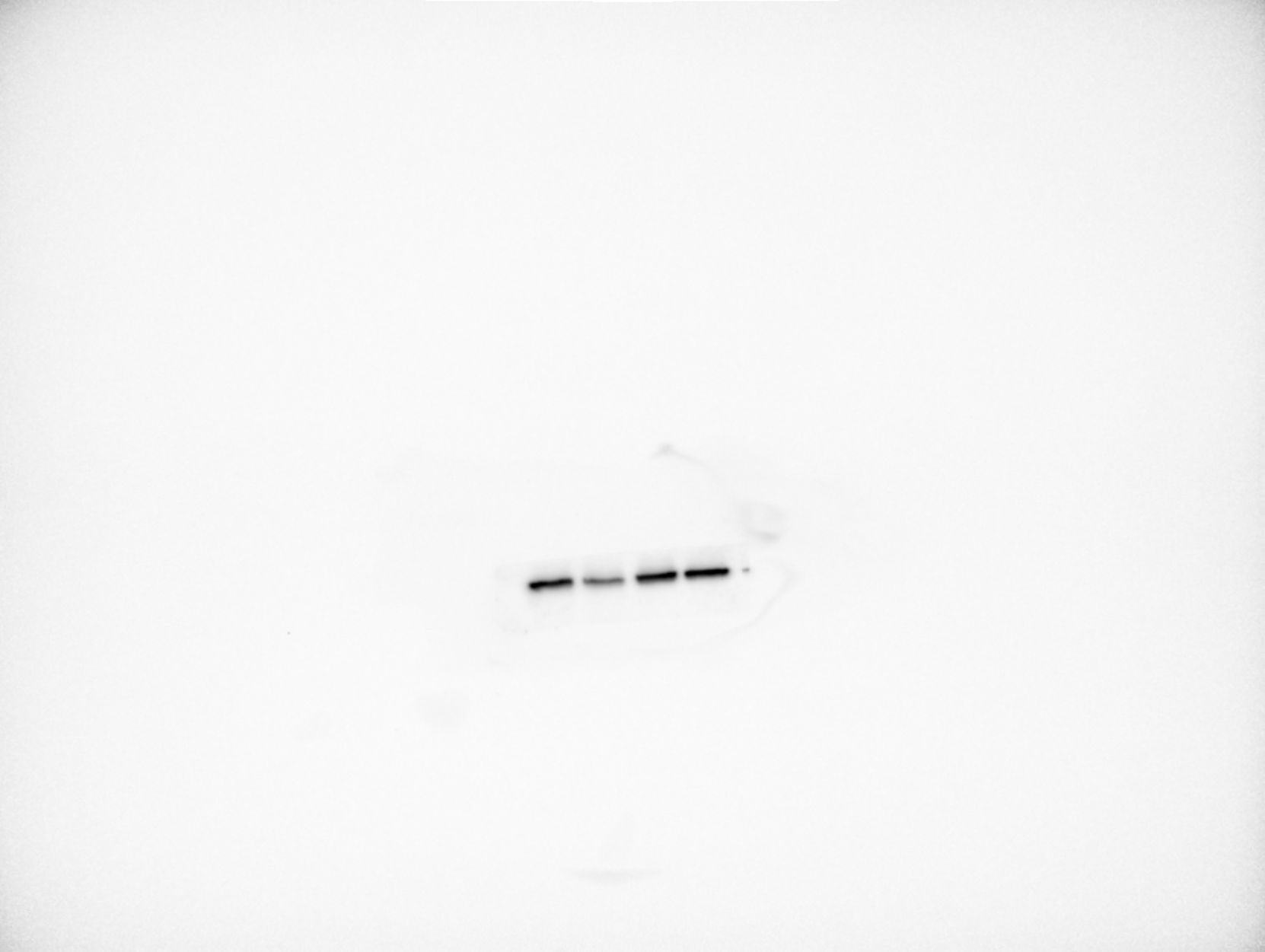

Supplement: Supplementary file 2 [file DataSheet1.ZIP › original WB photo/GLUT4-membrane/GLUT4-membrane-T_pub.jpg]

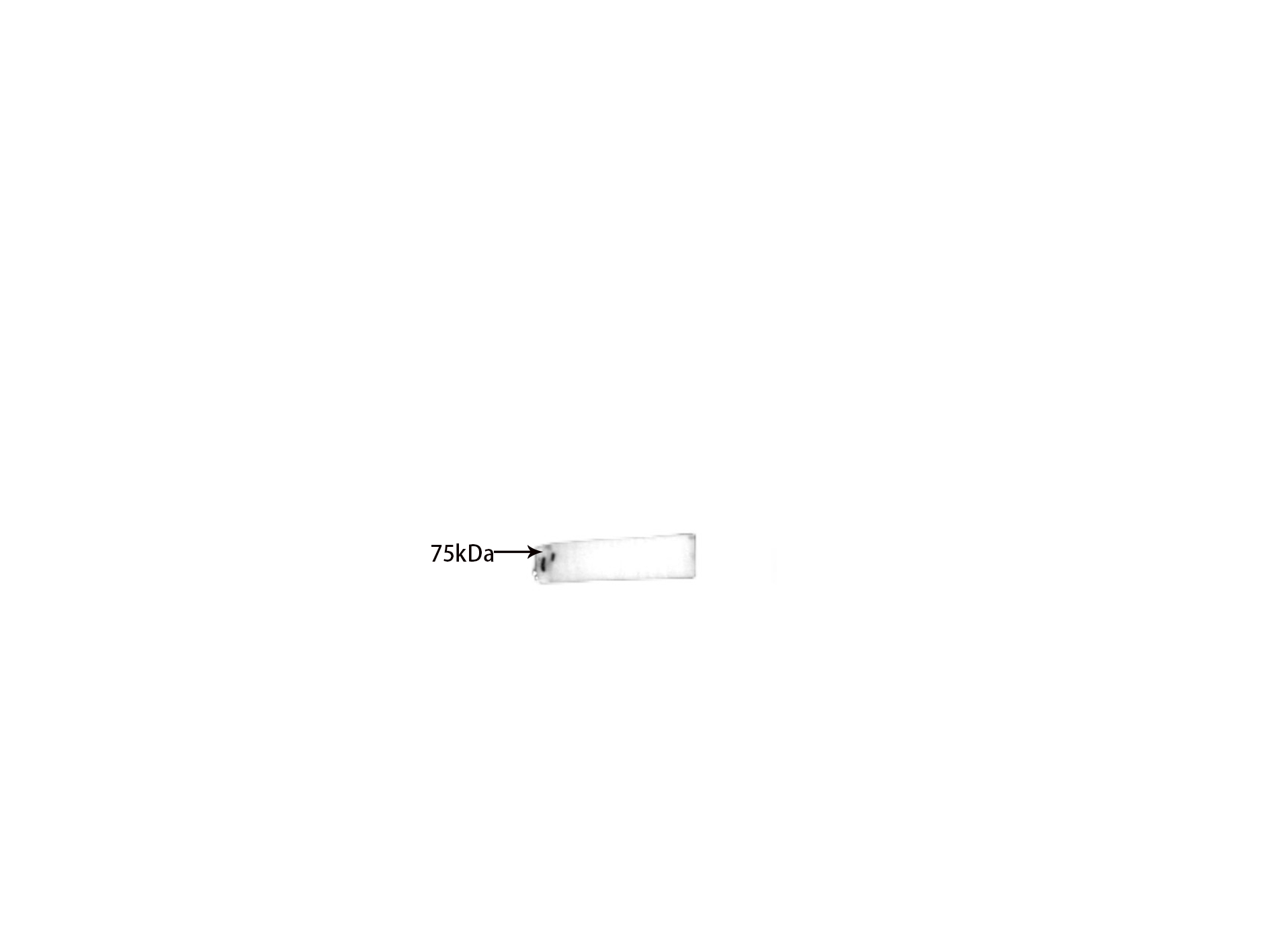

Supplement: Supplementary file 2 [file DataSheet1.ZIP › original WB photo/LB/LB-F-marker_pub.jpg]

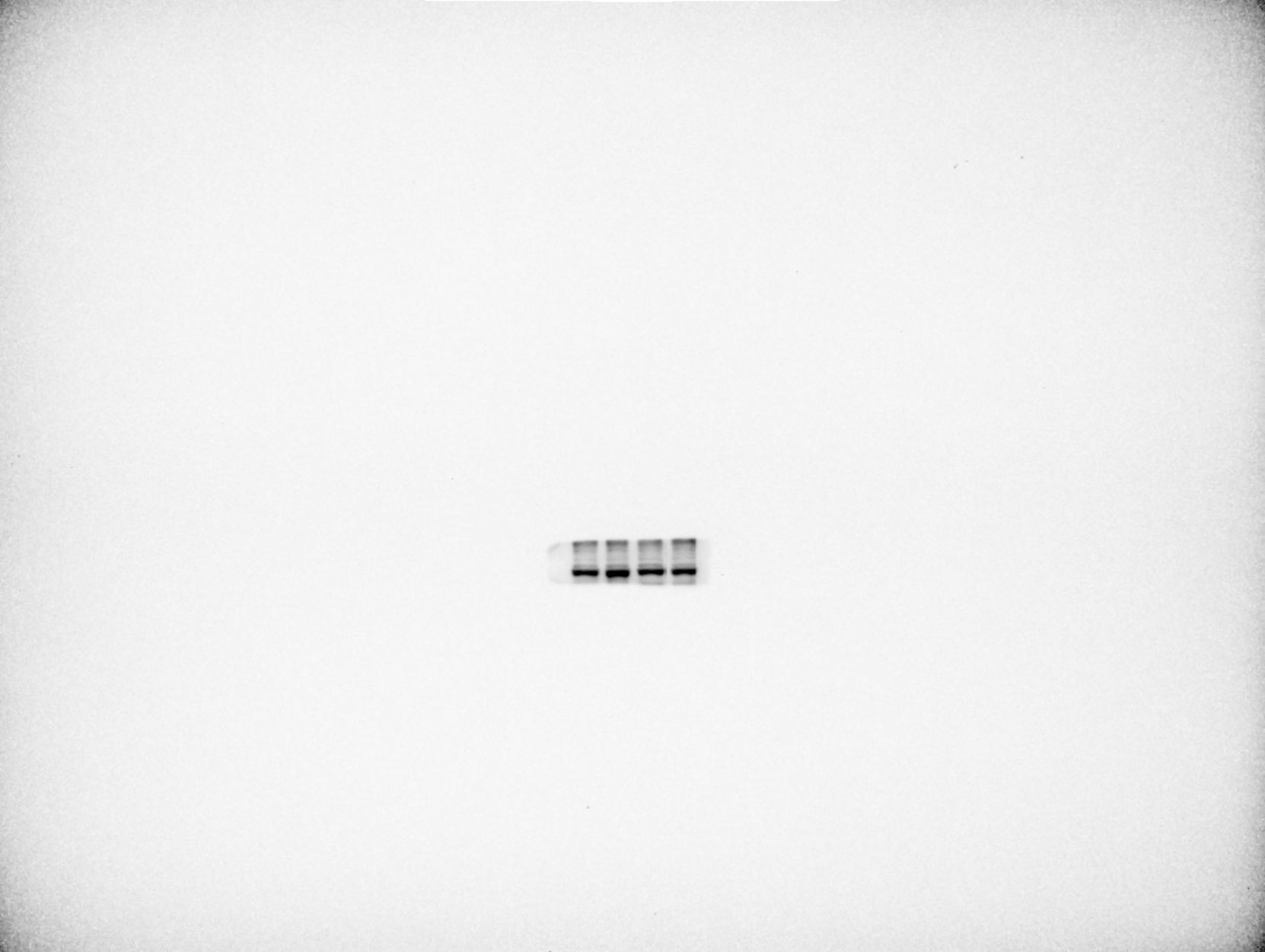

Supplement: Supplementary file 2 [file DataSheet1.ZIP › original WB photo/LB/LB-F_pub.jpg]

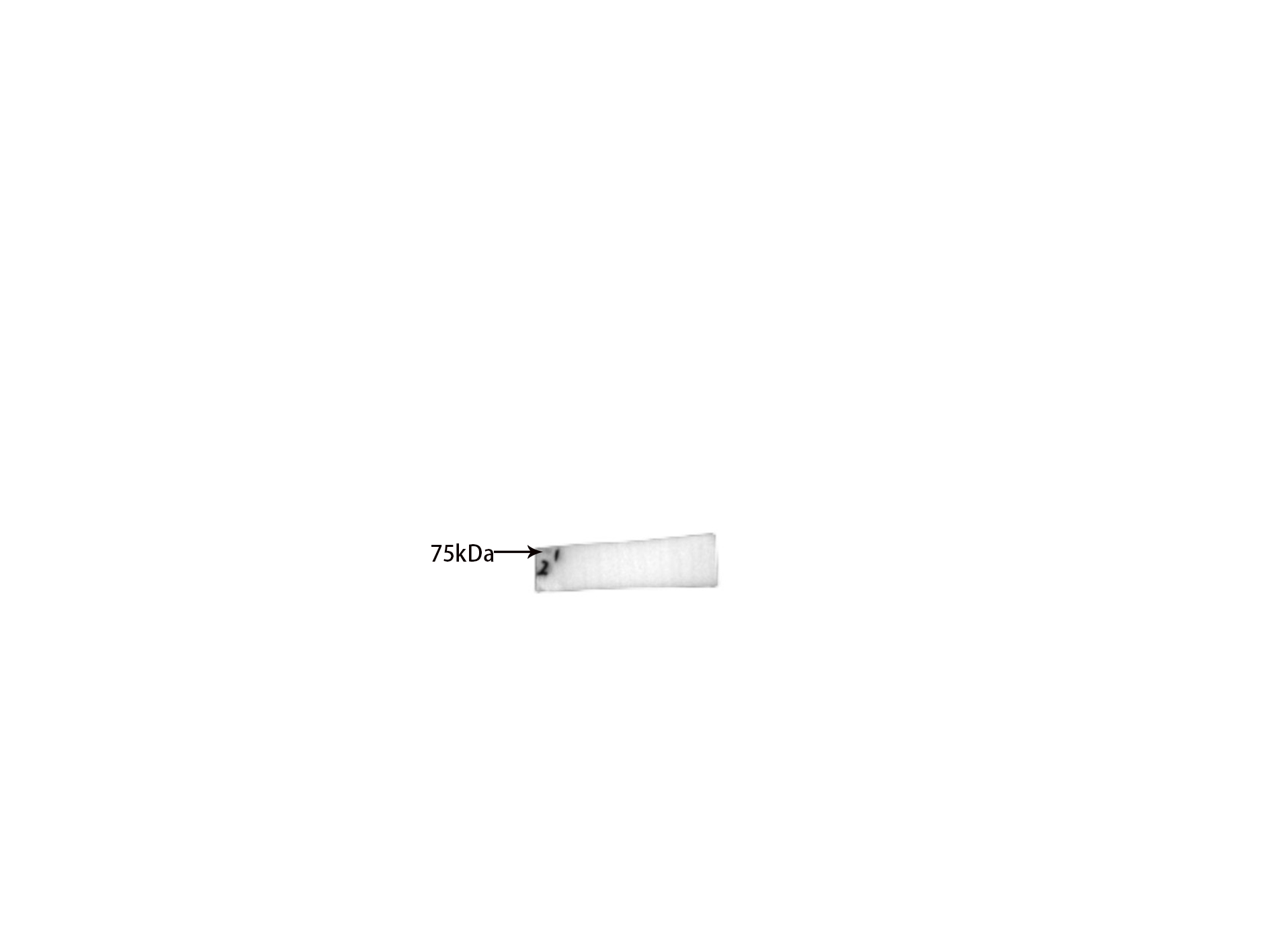

Supplement: Supplementary file 2 [file DataSheet1.ZIP › original WB photo/LB/LB-S-marker_pub.jpg]

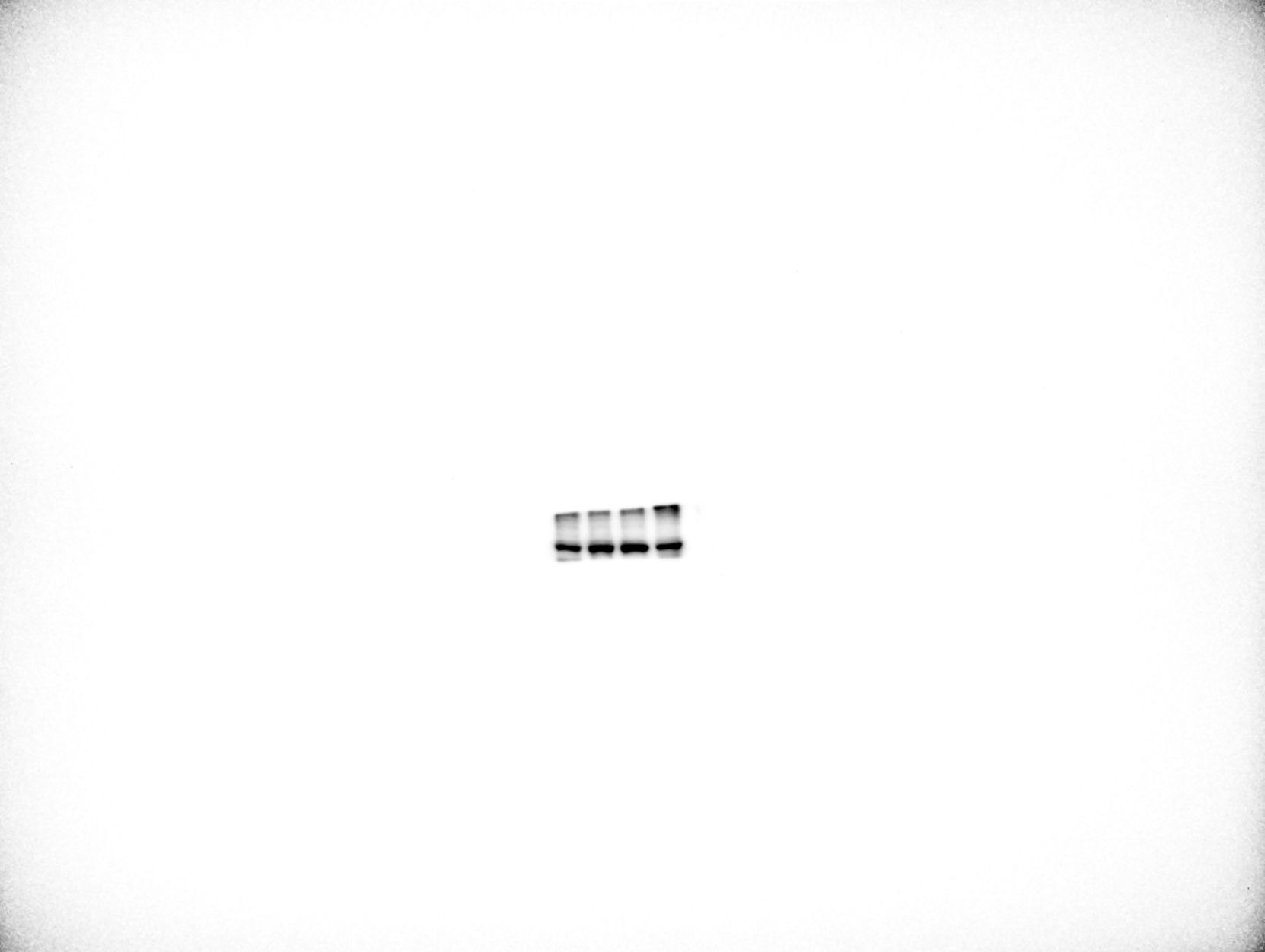

Supplement: Supplementary file 2 [file DataSheet1.ZIP › original WB photo/LB/LB-S_pub.jpg]

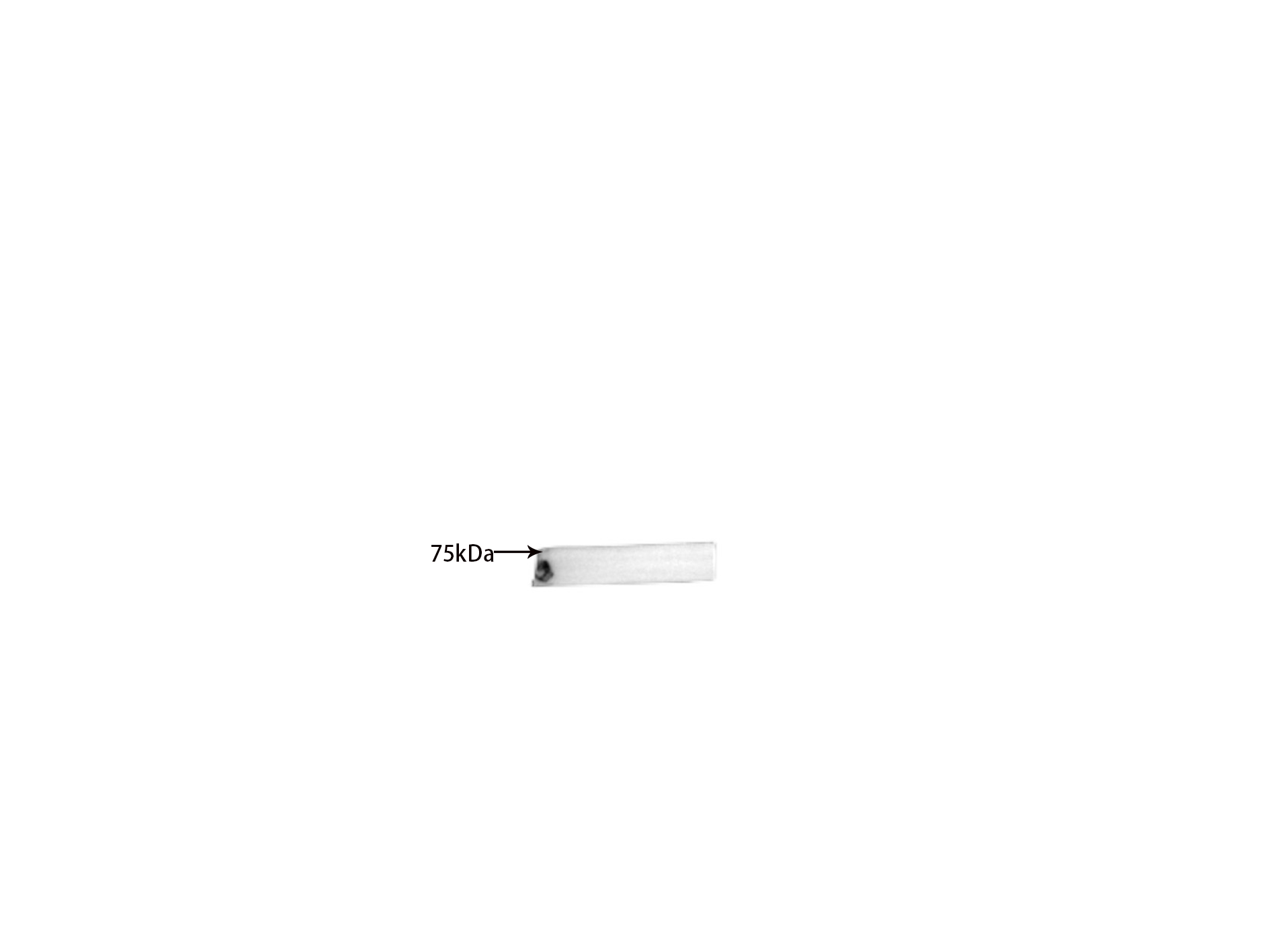

Supplement: Supplementary file 2 [file DataSheet1.ZIP › original WB photo/LB/LB-T-marker_pub.jpg]

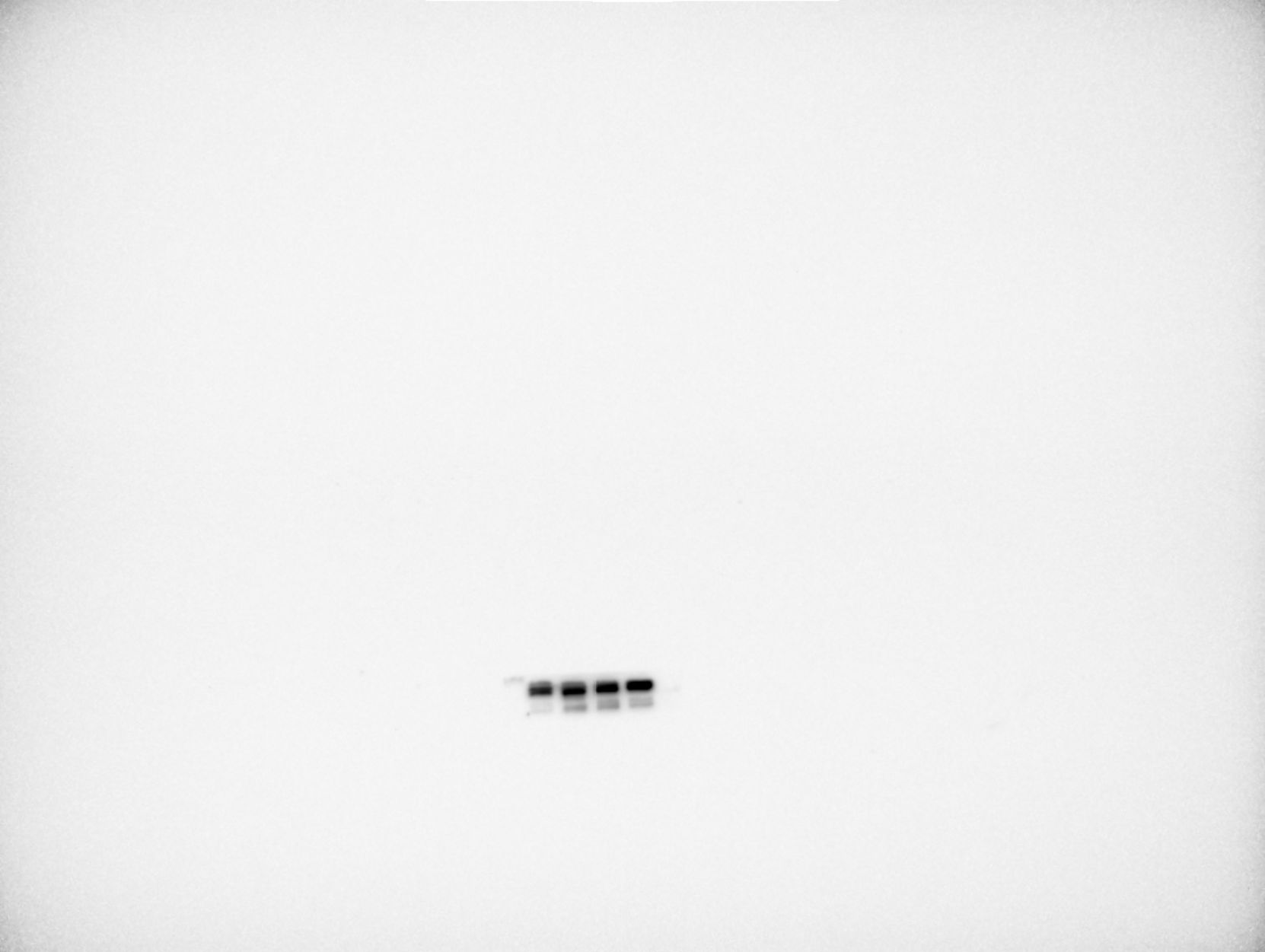

Supplement: Supplementary file 2 [file DataSheet1.ZIP › original WB photo/LB/LB-T_pub.jpg]

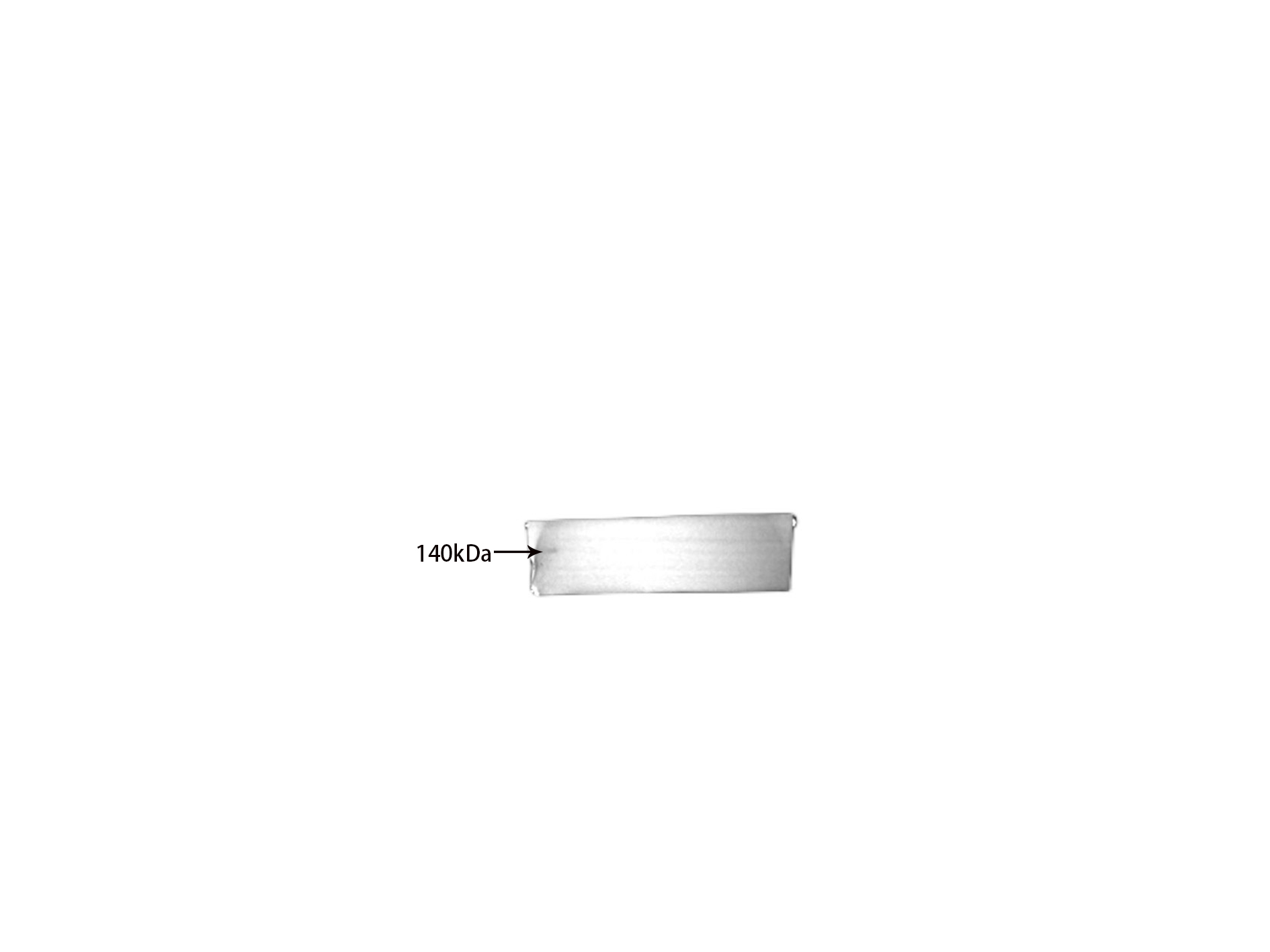

Supplement: Supplementary file 2 [file DataSheet1.ZIP › original WB photo/Na,K-ATPase/Na,K-ATPase-F-marker_pub.jpg]

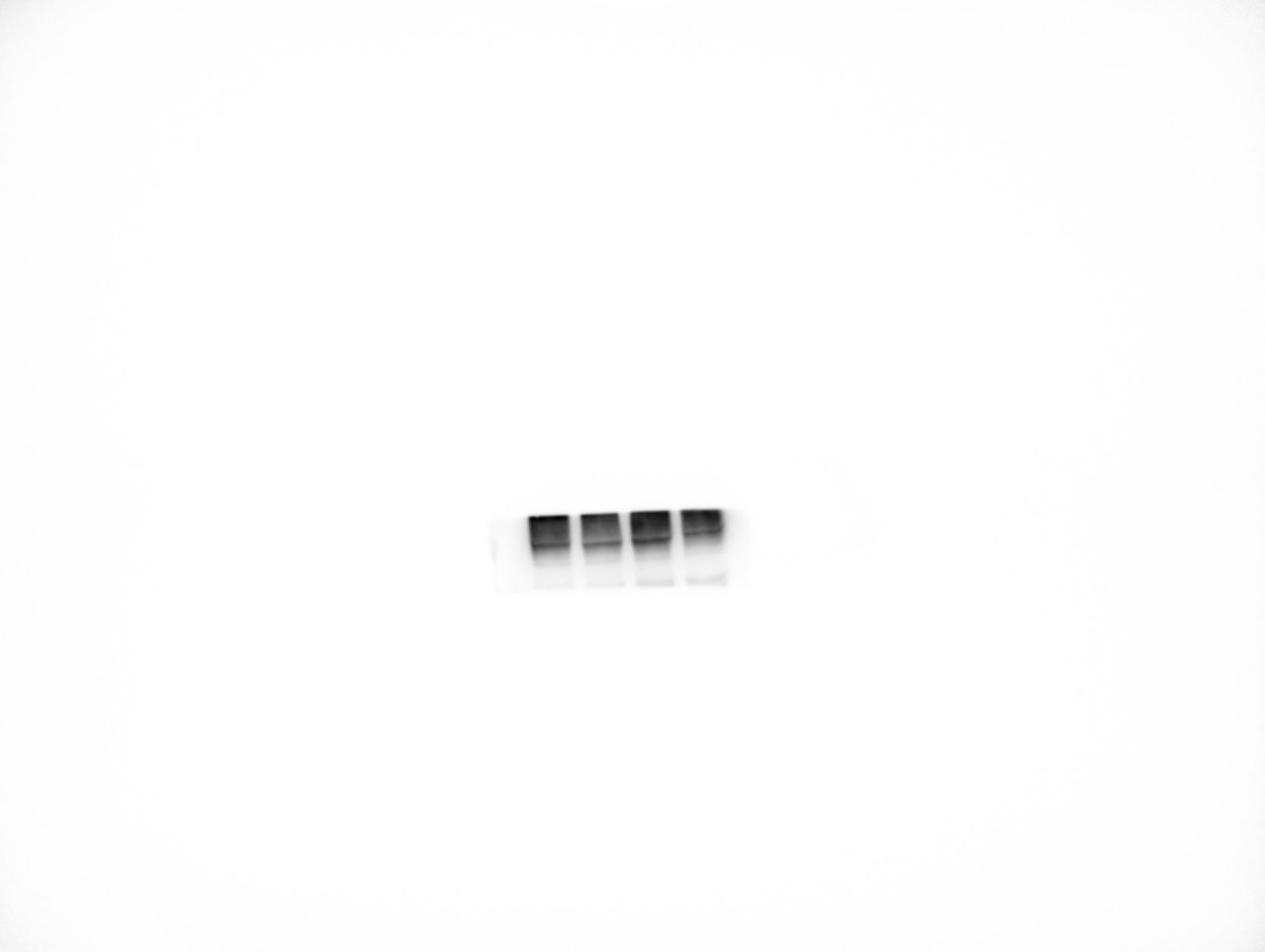

Supplement: Supplementary file 2 [file DataSheet1.ZIP › original WB photo/Na,K-ATPase/Na,K-ATPase-F_pub.jpg]

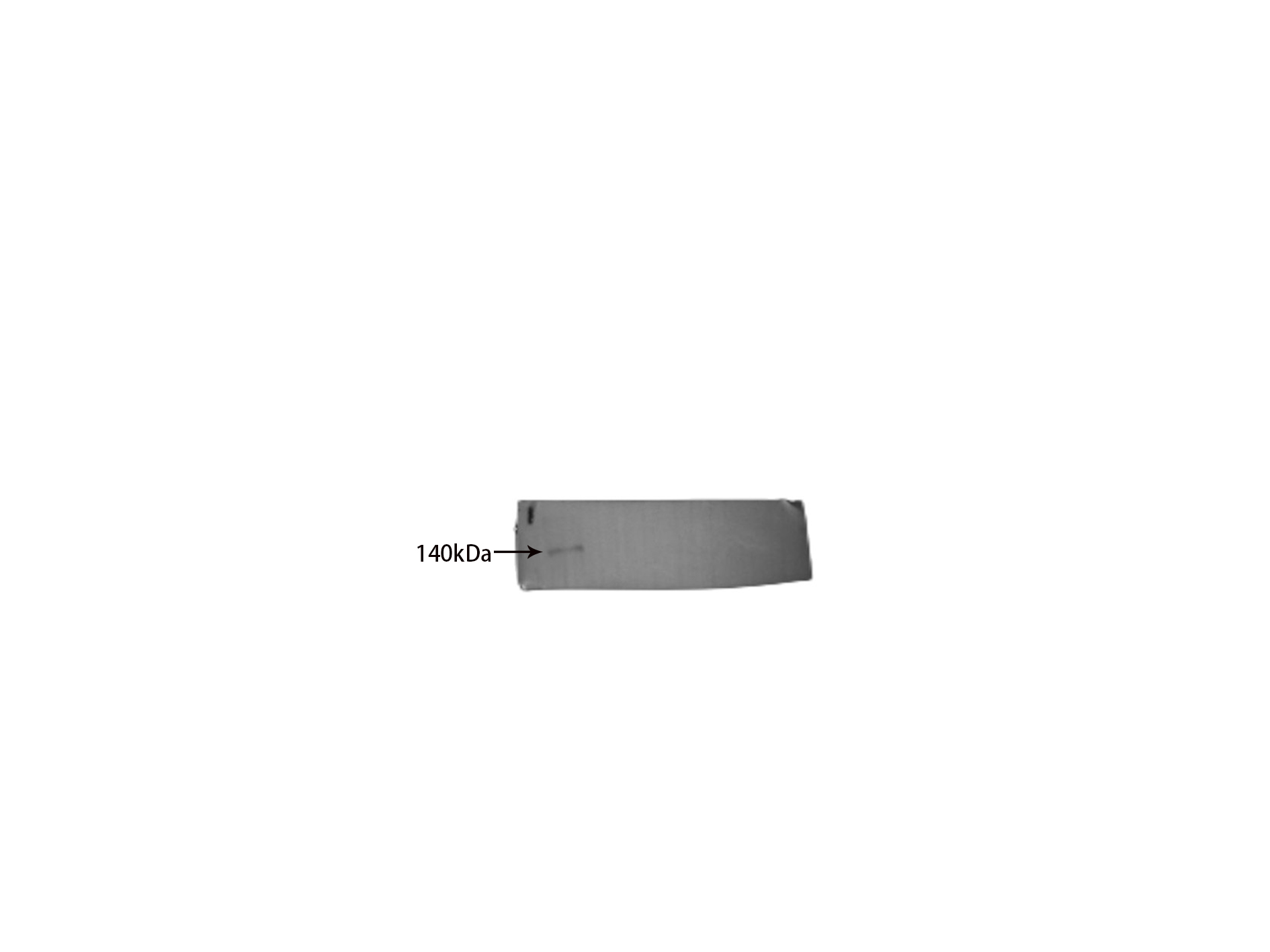

Supplement: Supplementary file 2 [file DataSheet1.ZIP › original WB photo/Na,K-ATPase/Na,K-ATPase-S-marker_pub.jpg]

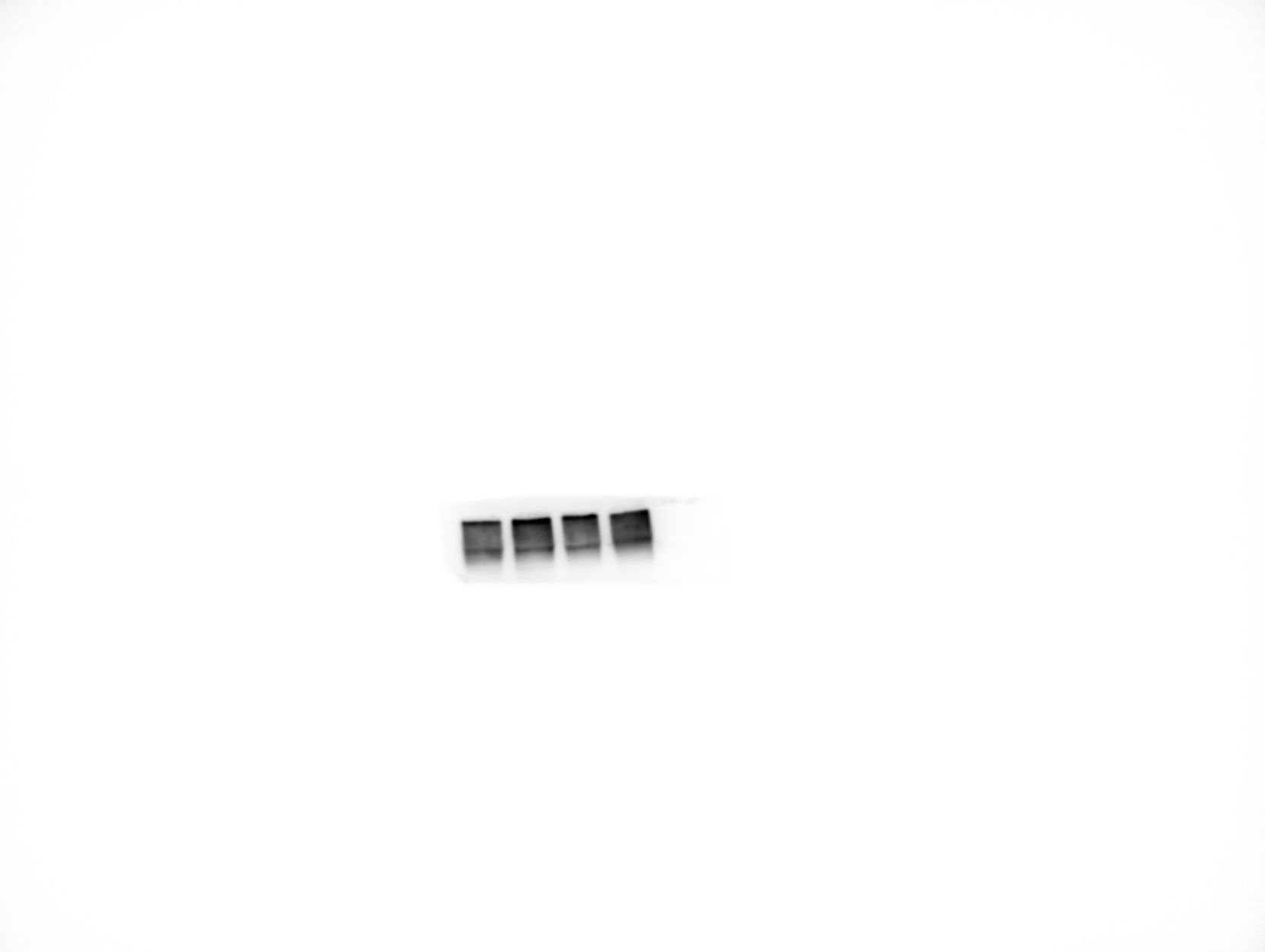

Supplement: Supplementary file 2 [file DataSheet1.ZIP › original WB photo/Na,K-ATPase/Na,K-ATPase-S_pub.jpg]

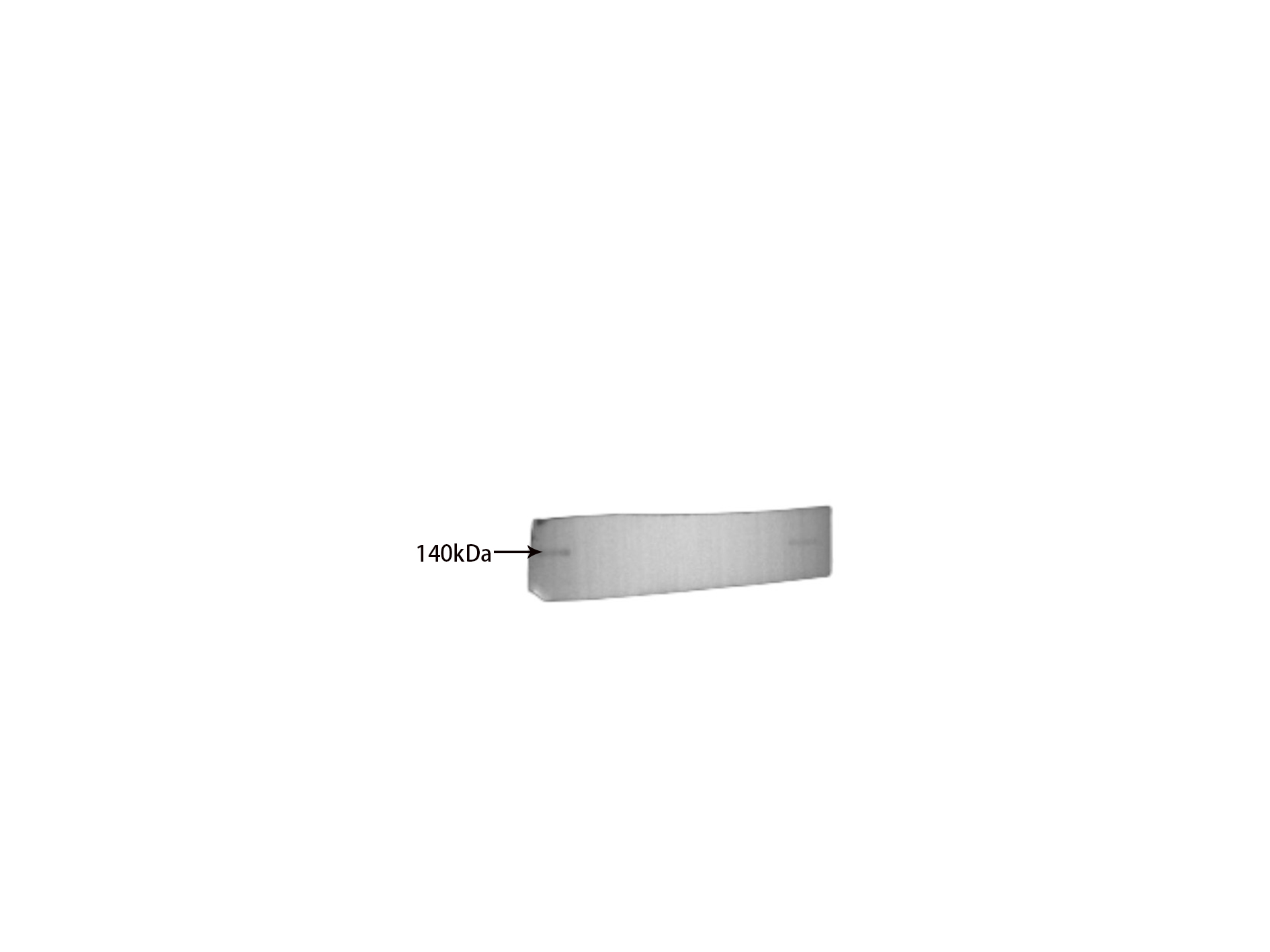

Supplement: Supplementary file 2 [file DataSheet1.ZIP › original WB photo/Na,K-ATPase/Na,K-ATPase-T-marker_pub.jpg]

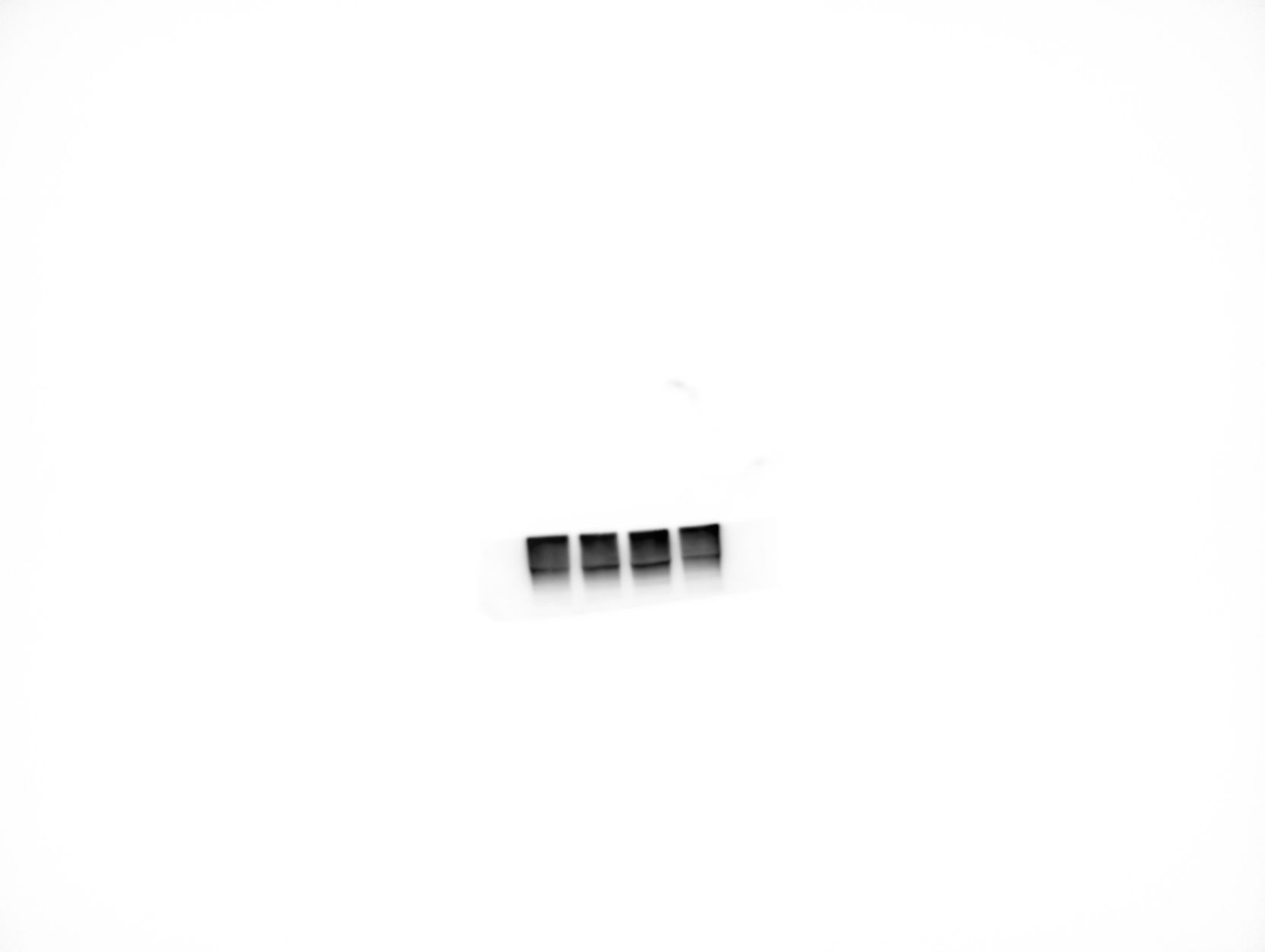

Supplement: Supplementary file 2 [file DataSheet1.ZIP › original WB photo/Na,K-ATPase/Na,K-ATPase-T_pub.jpg]

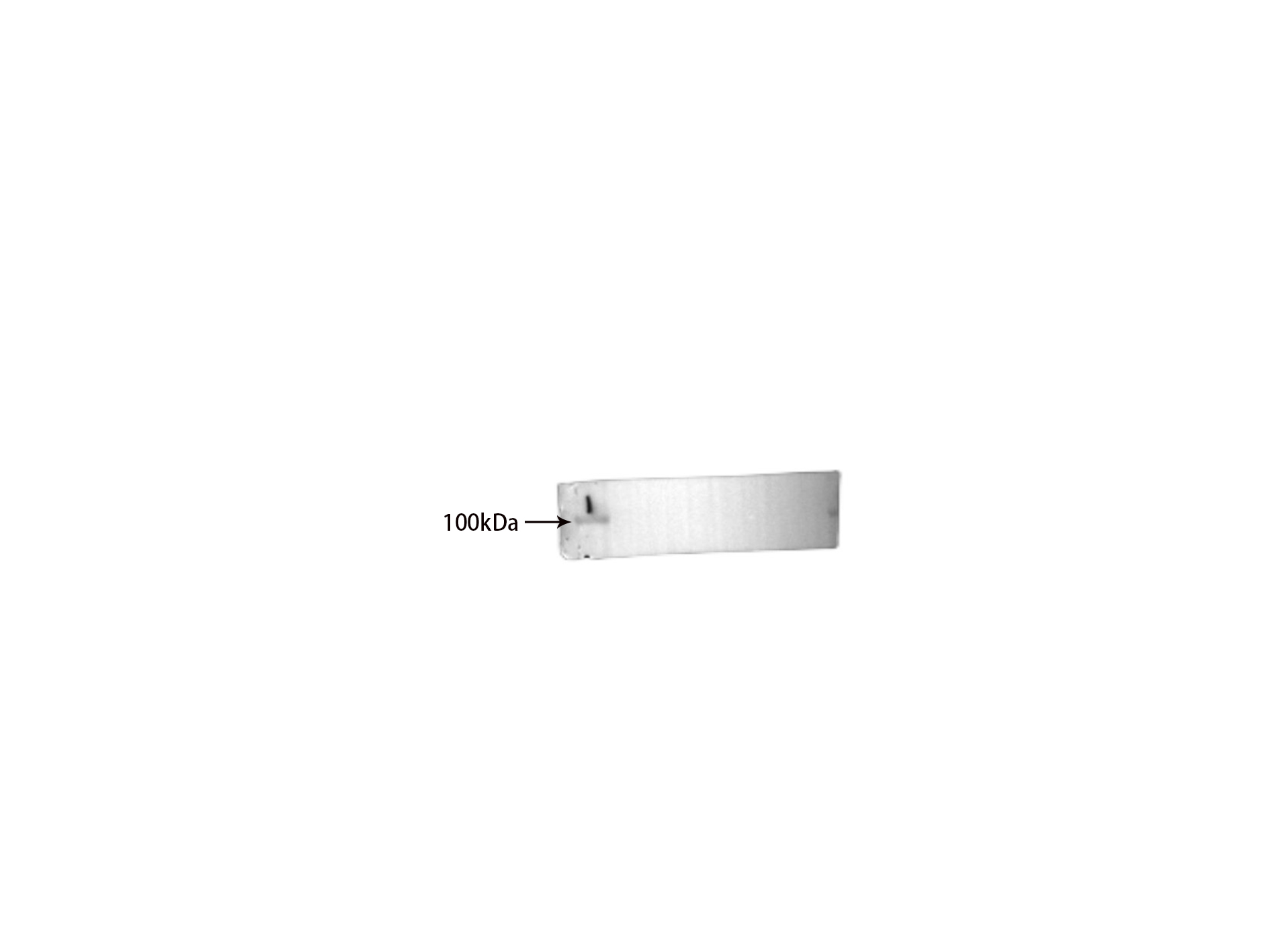

Supplement: Supplementary file 2 [file DataSheet1.ZIP › original WB photo/PGC-1a┴/PGC-1a┴-F-marker_pub.jpg]

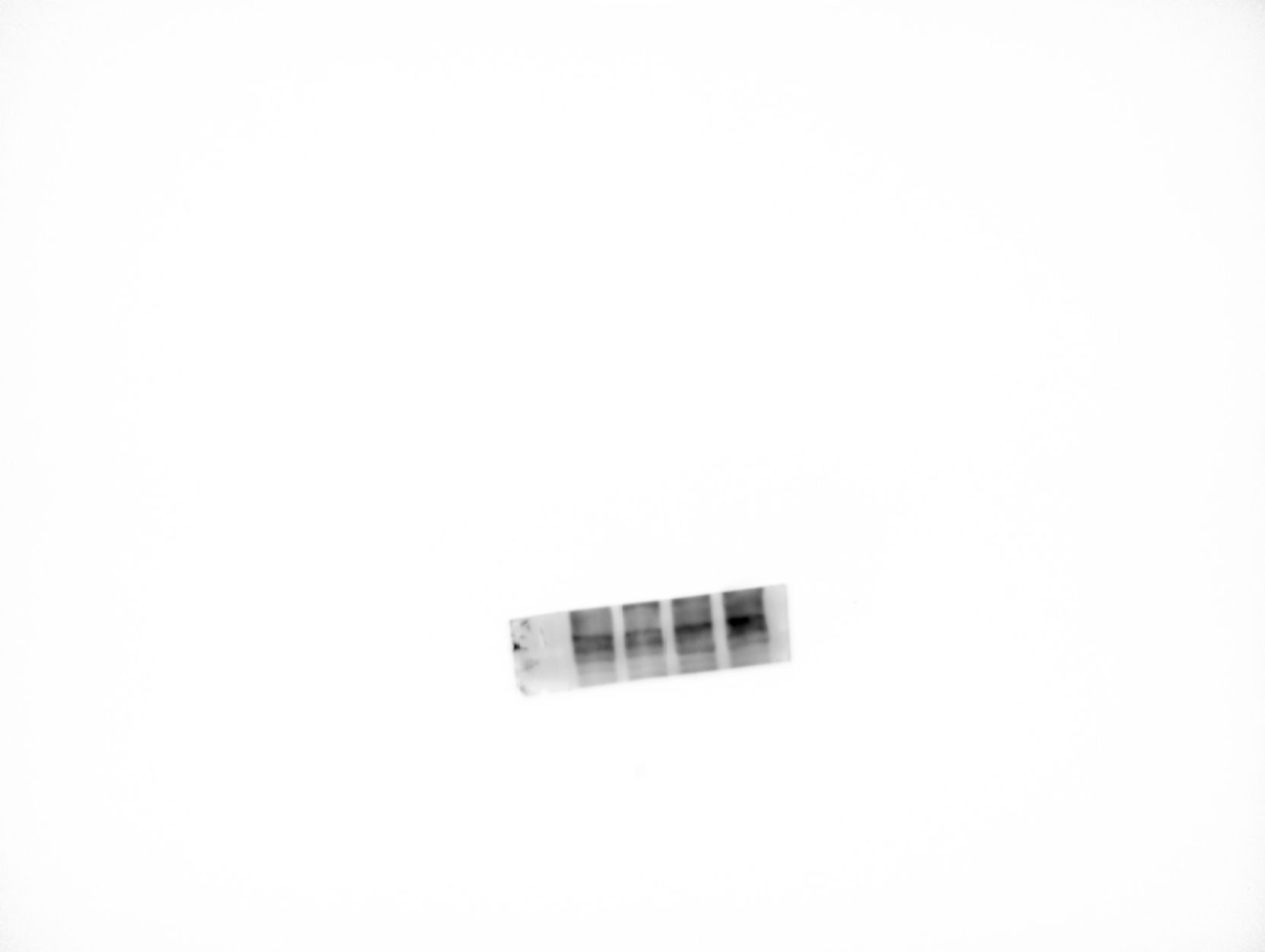

Supplement: Supplementary file 2 [file DataSheet1.ZIP › original WB photo/PGC-1a┴/PGC-1a┴-F_pub.jpg]

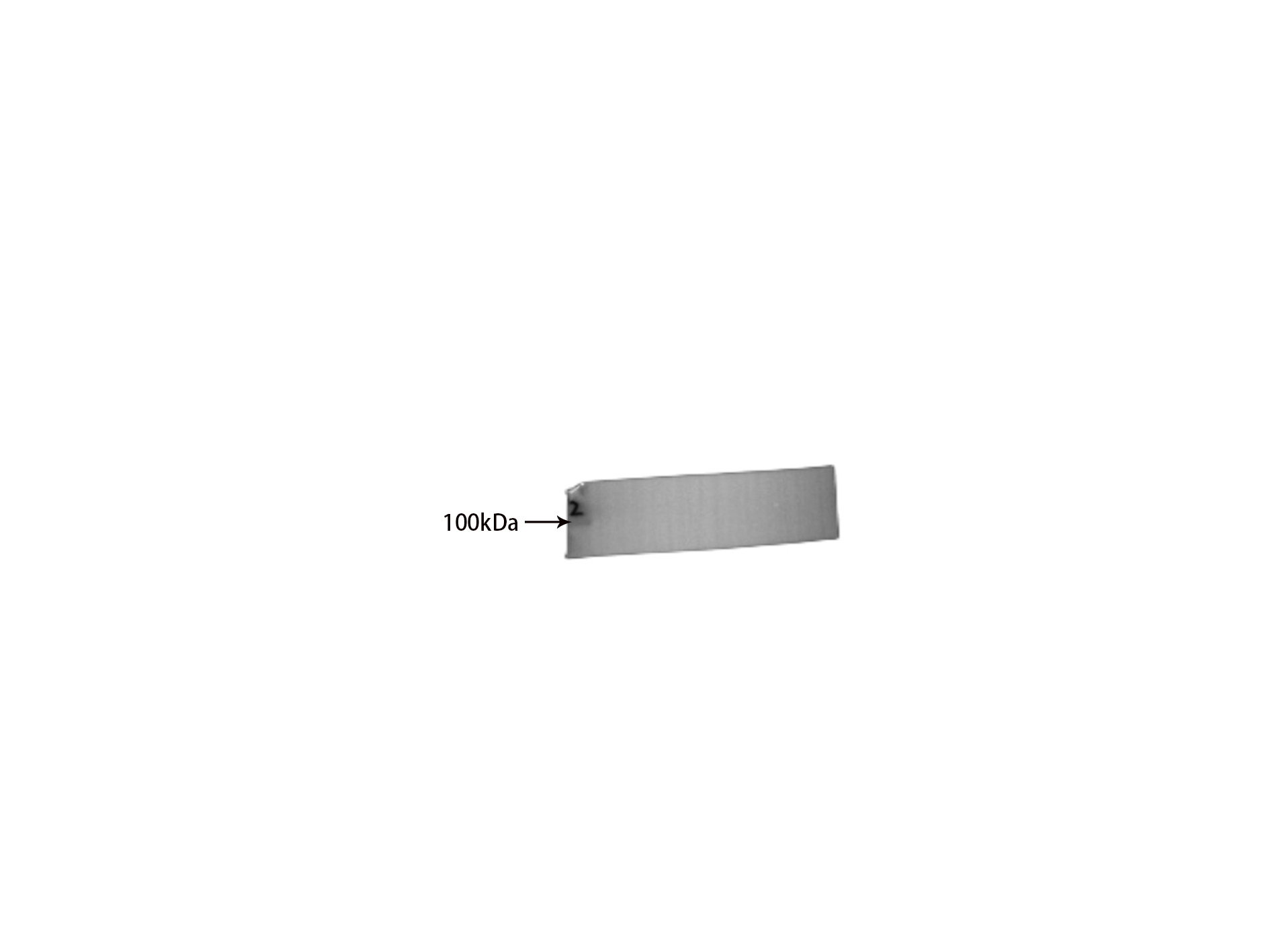

Supplement: Supplementary file 2 [file DataSheet1.ZIP › original WB photo/PGC-1a┴/PGC-1a┴-S-marker_pub.jpg]

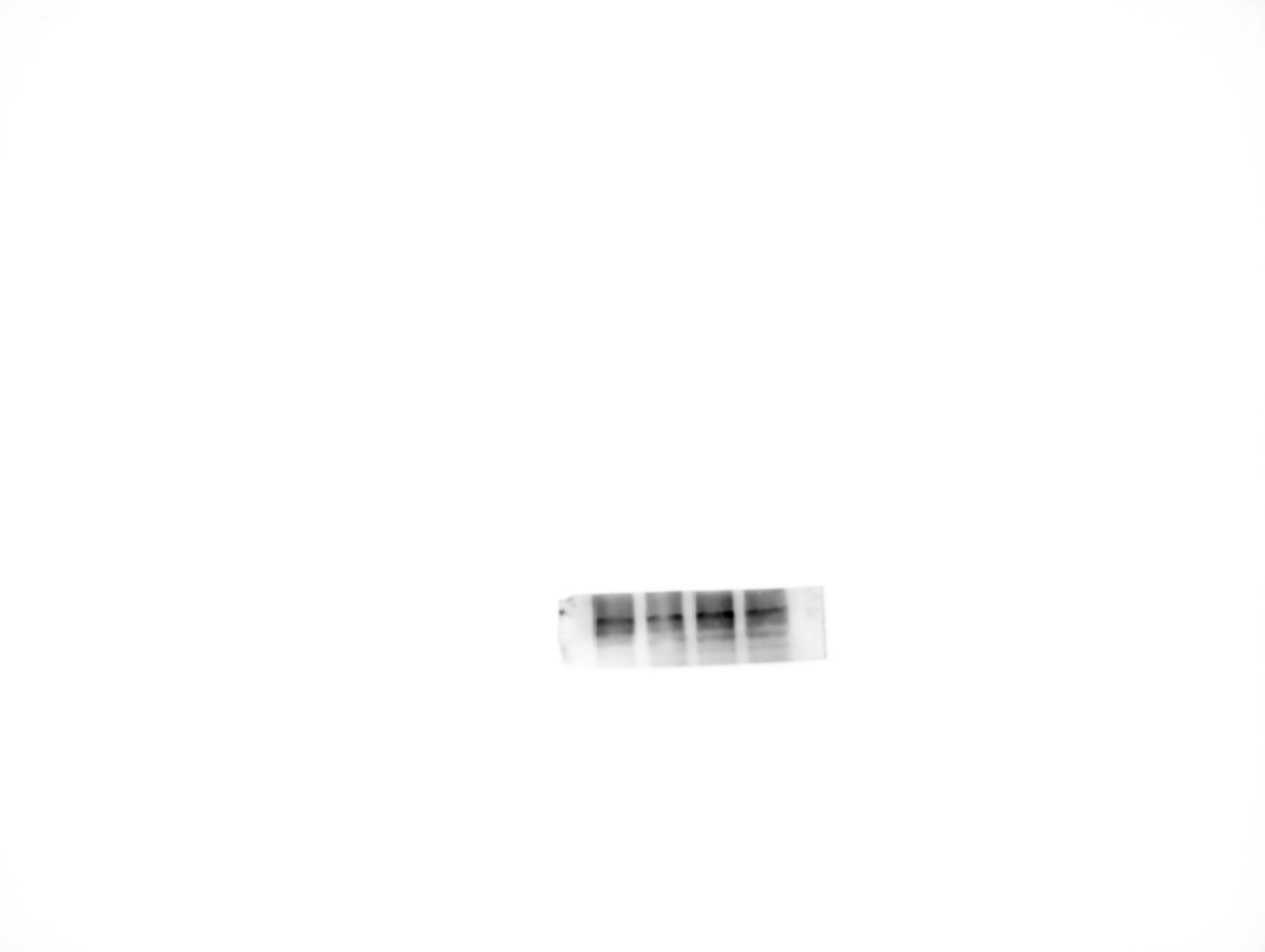

Supplement: Supplementary file 2 [file DataSheet1.ZIP › original WB photo/PGC-1a┴/PGC-1a┴-S_pub.jpg]

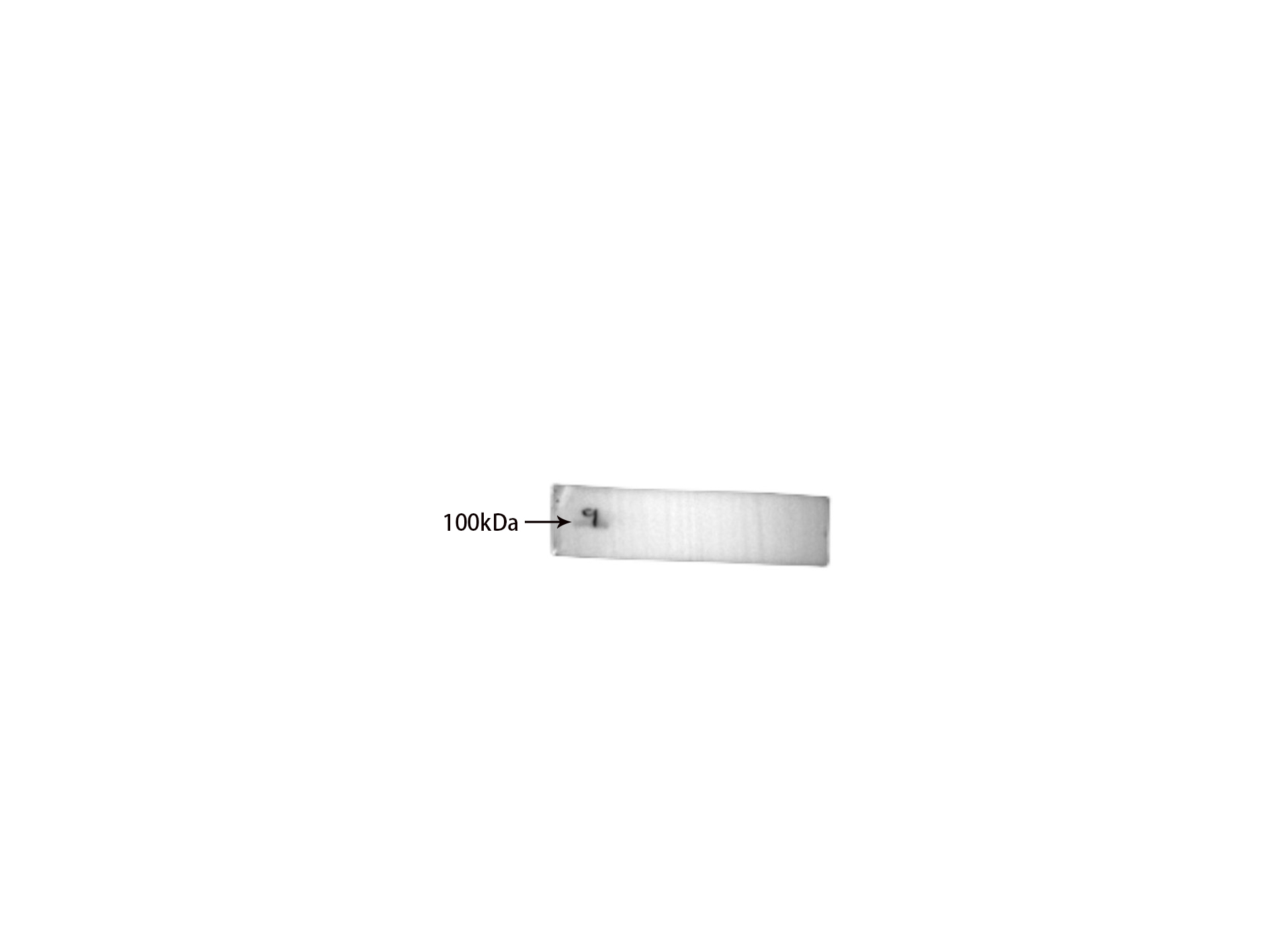

Supplement: Supplementary file 2 [file DataSheet1.ZIP › original WB photo/PGC-1a┴/PGC-1a┴-T-marker_pub.jpg]

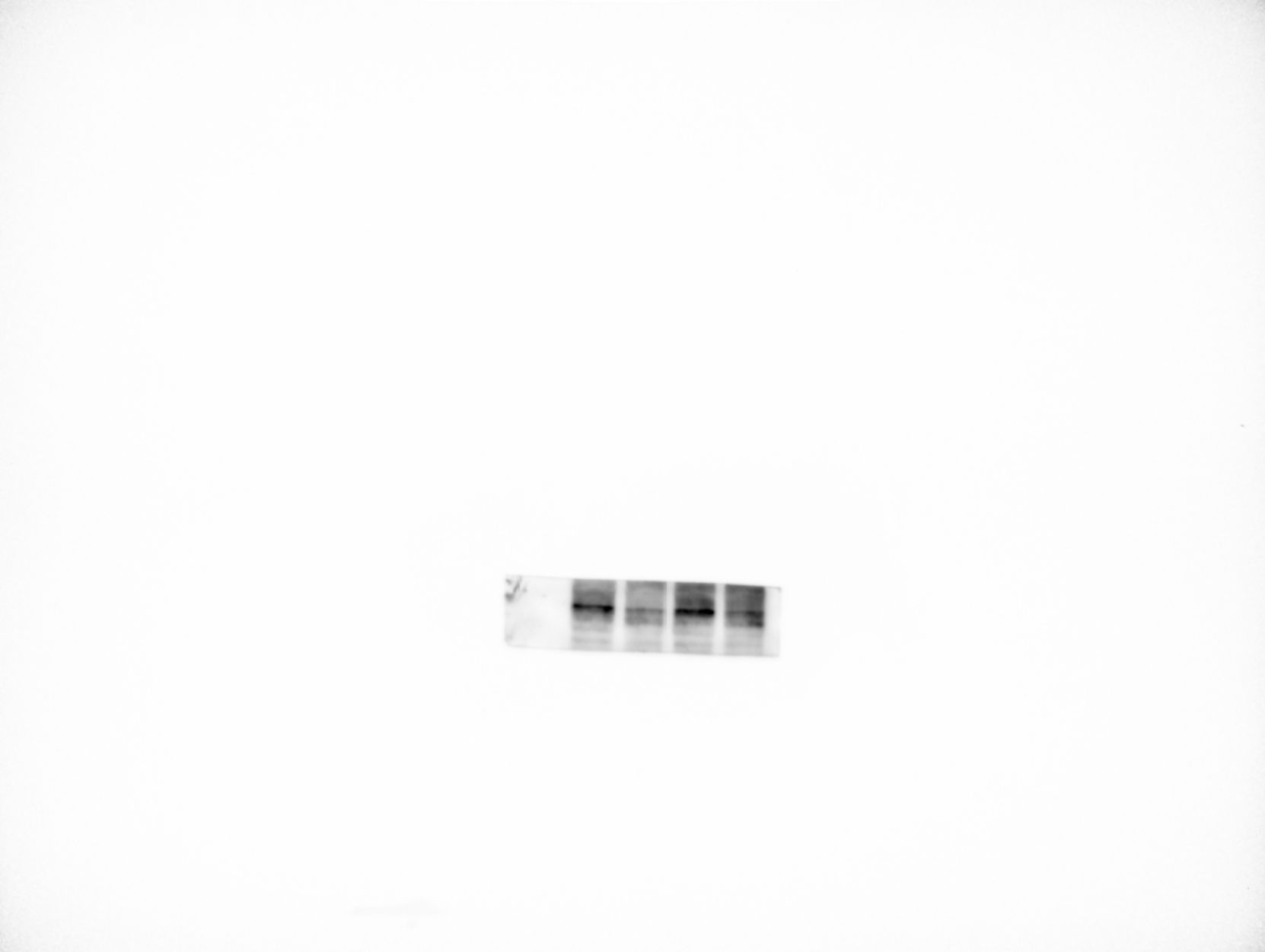

Supplement: Supplementary file 2 [file DataSheet1.ZIP › original WB photo/PGC-1a┴/PGC-1a┴-T_pub.jpg]

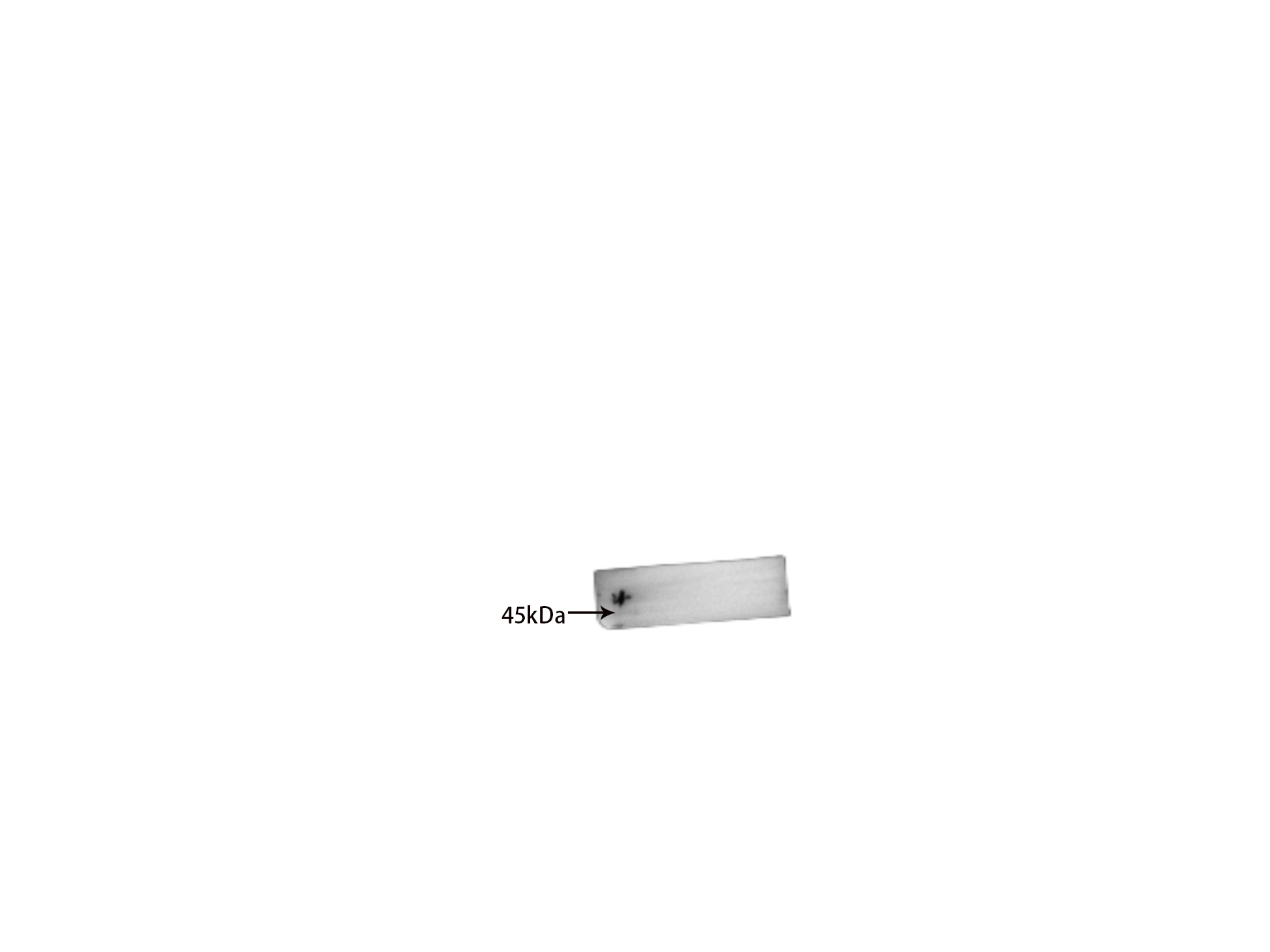

Supplement: Supplementary file 2 [file DataSheet1.ZIP › original WB photo/Ppara-nucleus/Ppara-nucleus-F-marker_pub.jpg]

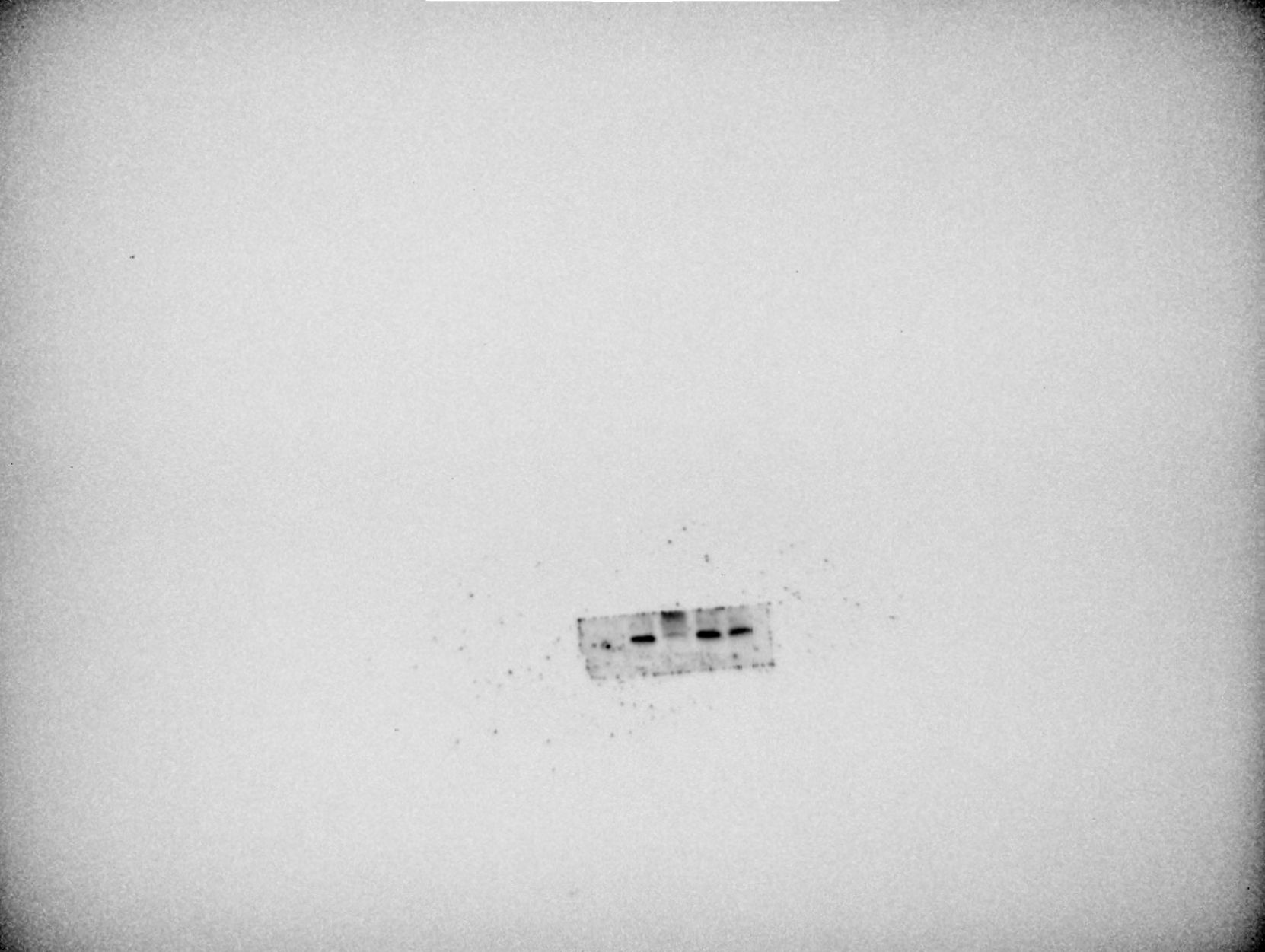

Supplement: Supplementary file 2 [file DataSheet1.ZIP › original WB photo/Ppara-nucleus/Ppara-nucleus-F_pub.jpg]

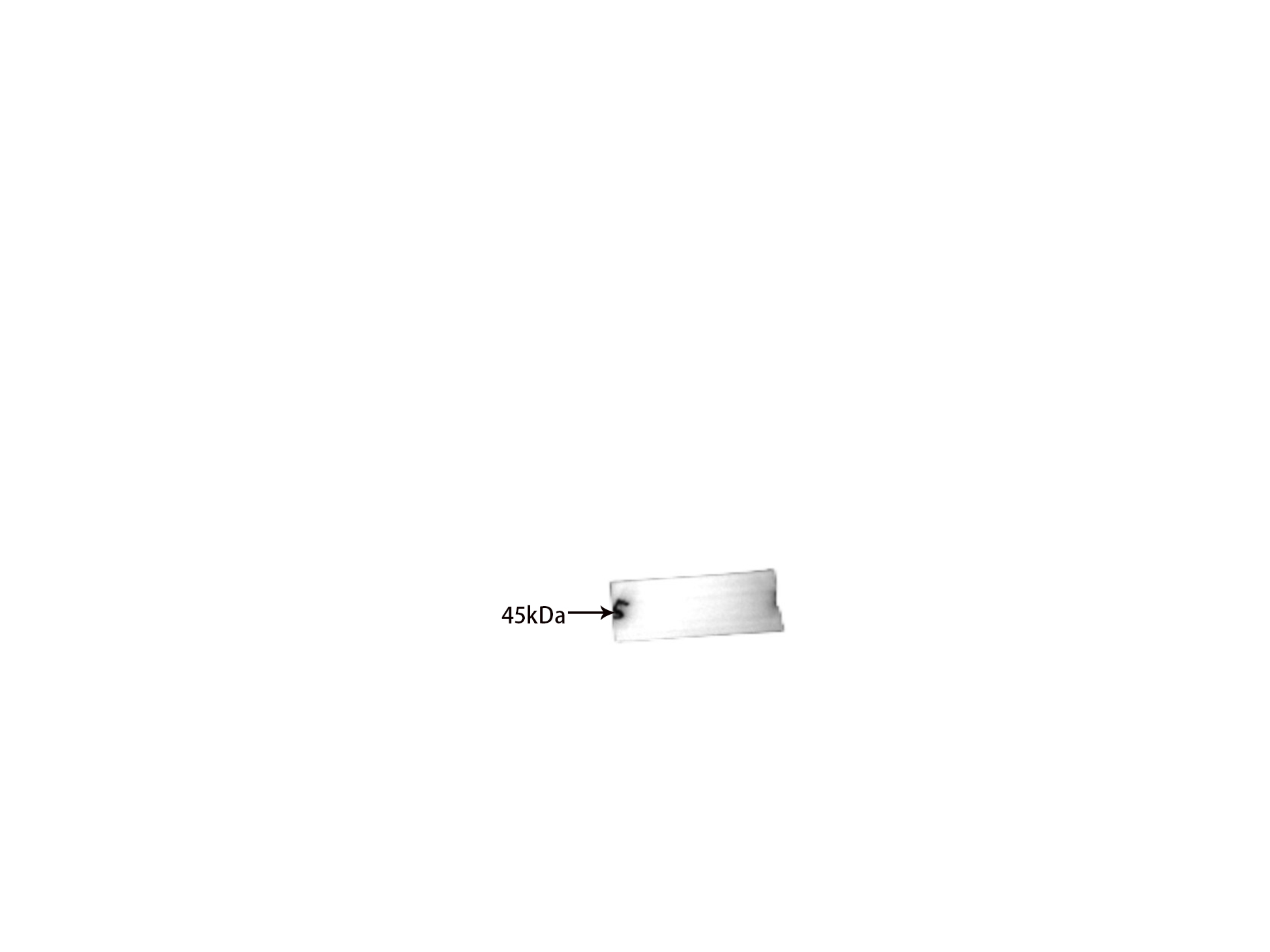

Supplement: Supplementary file 2 [file DataSheet1.ZIP › original WB photo/Ppara-nucleus/Ppara-nucleus-S-marker_pub.jpg]

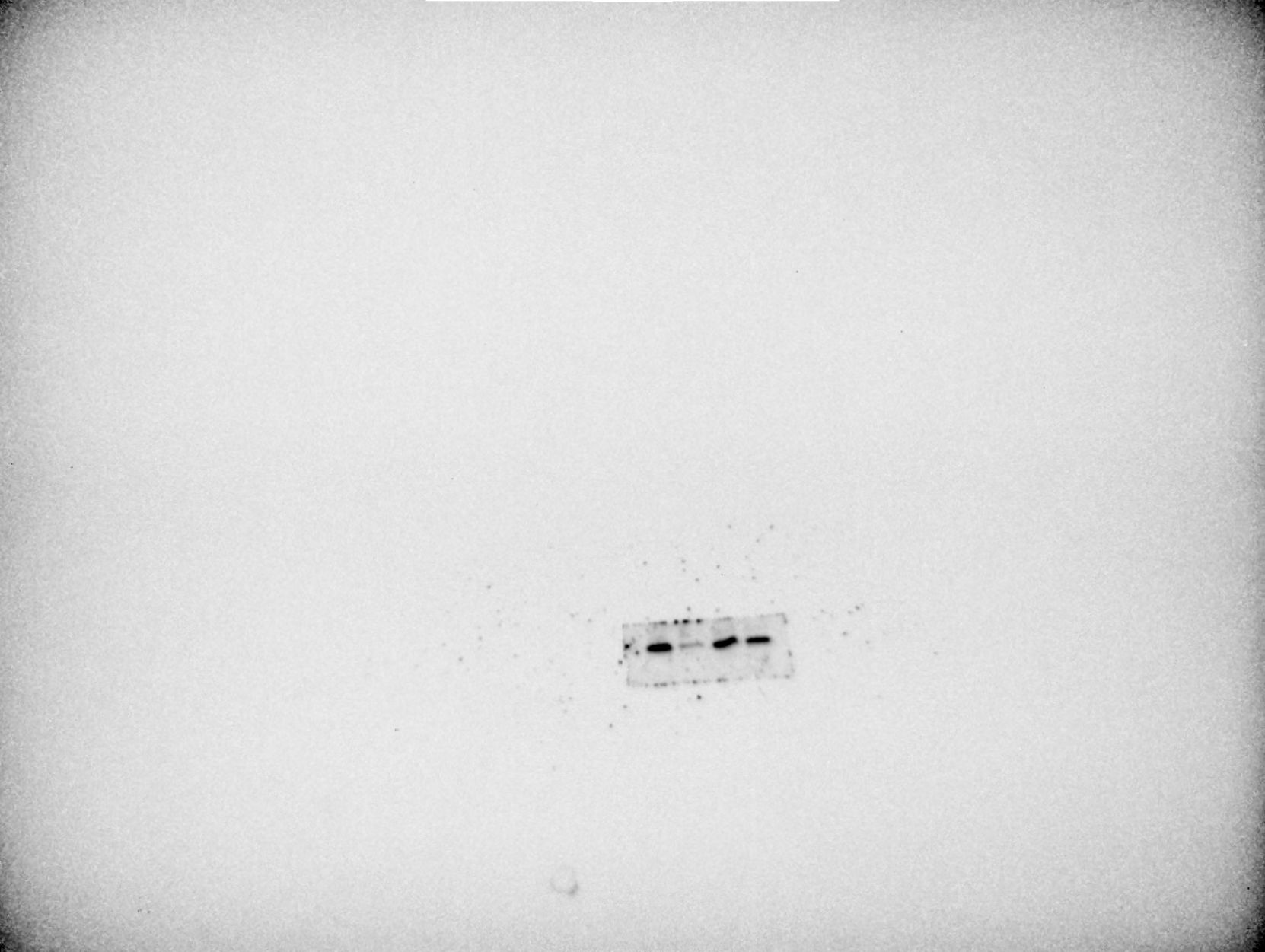

Supplement: Supplementary file 2 [file DataSheet1.ZIP › original WB photo/Ppara-nucleus/Ppara-nucleus-S_pub.jpg]

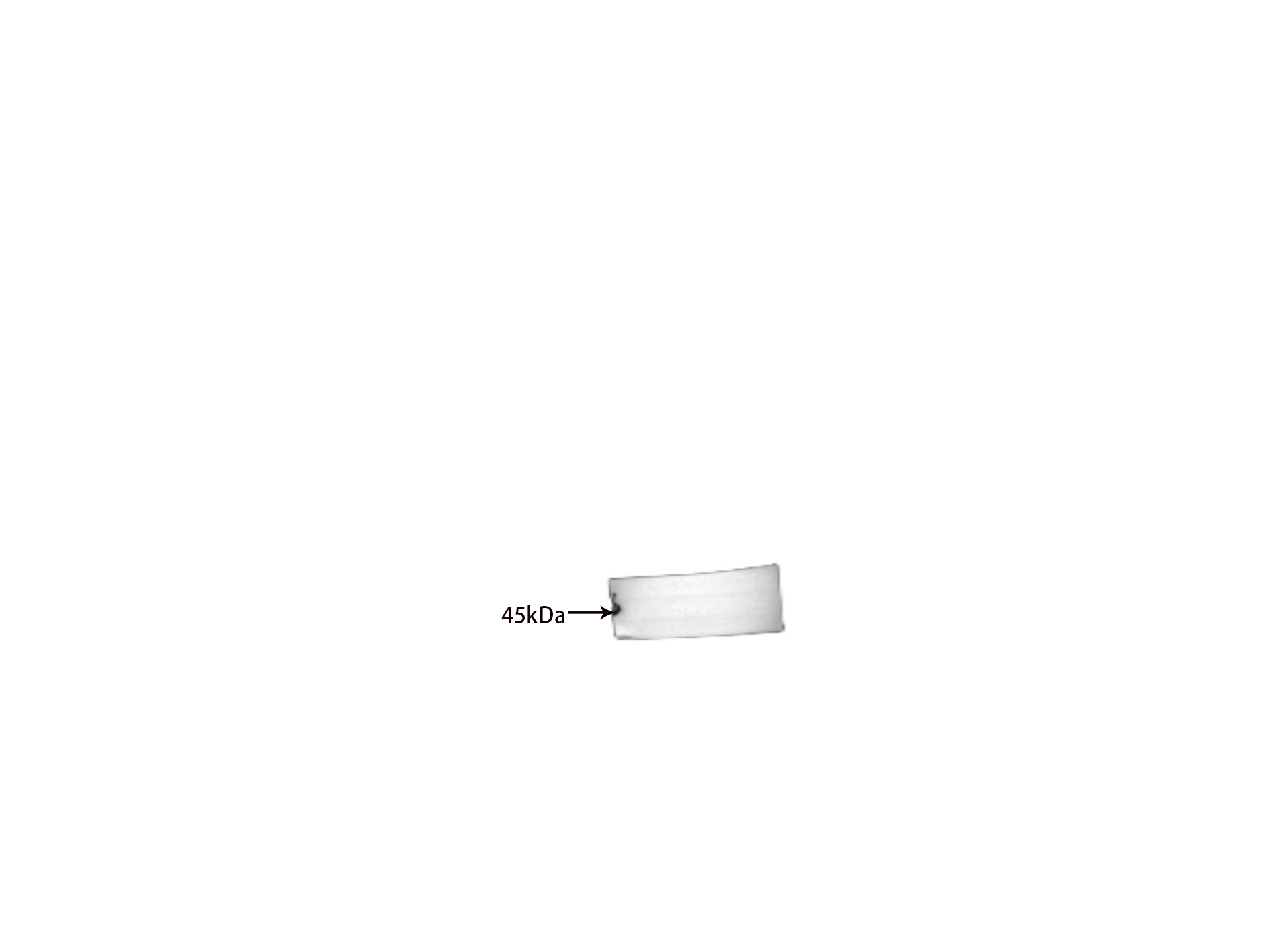

Supplement: Supplementary file 2 [file DataSheet1.ZIP › original WB photo/Ppara-nucleus/Ppara-nucleus-T-marker_pub.jpg]

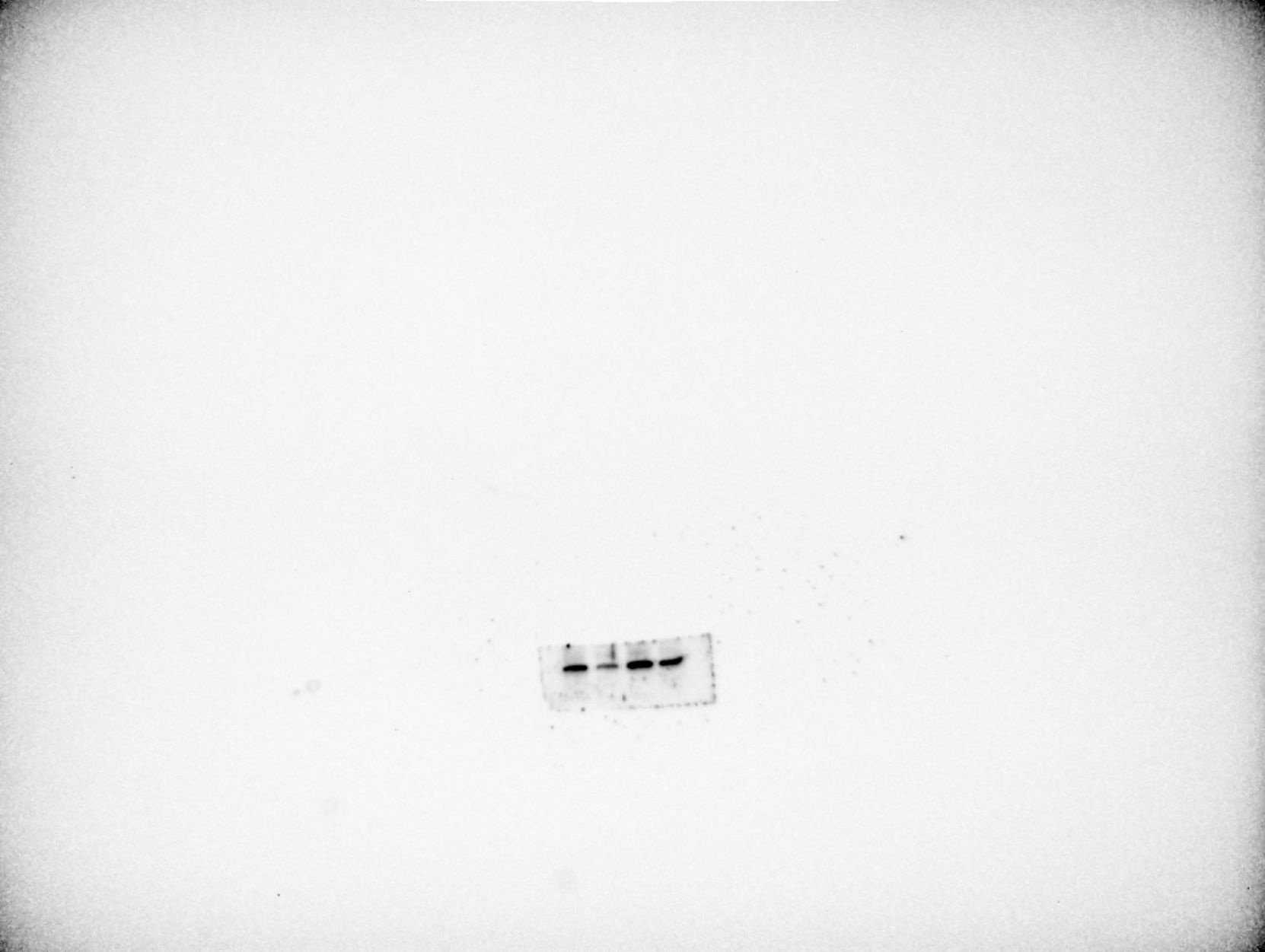

Supplement: Supplementary file 2 [file DataSheet1.ZIP › original WB photo/Ppara-nucleus/Ppara-nucleus-T_pub.jpg]

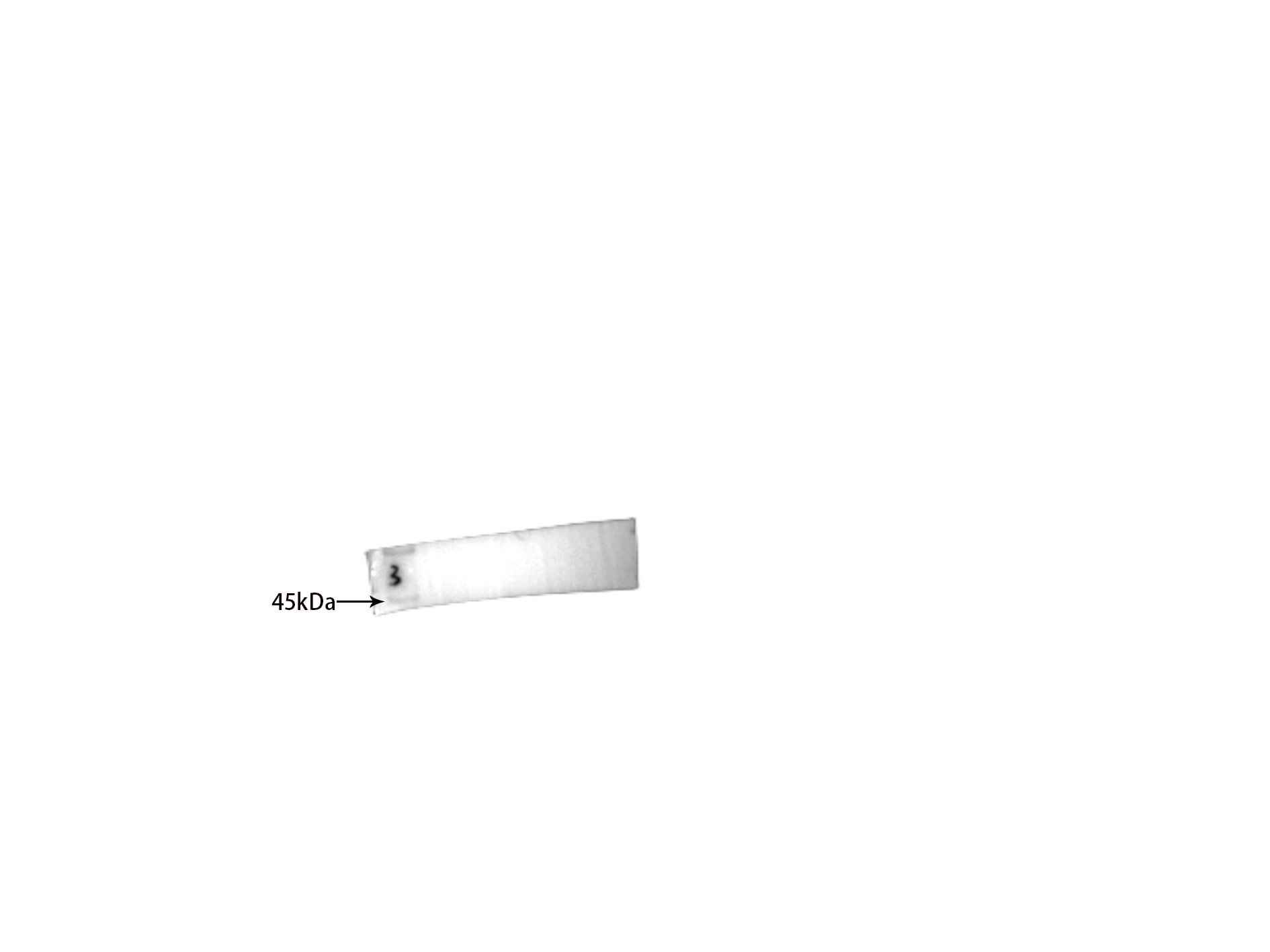

Supplement: Supplementary file 2 [file DataSheet1.ZIP › original WB photo/Ppara┴/Ppara-F-marker_pub.jpg]

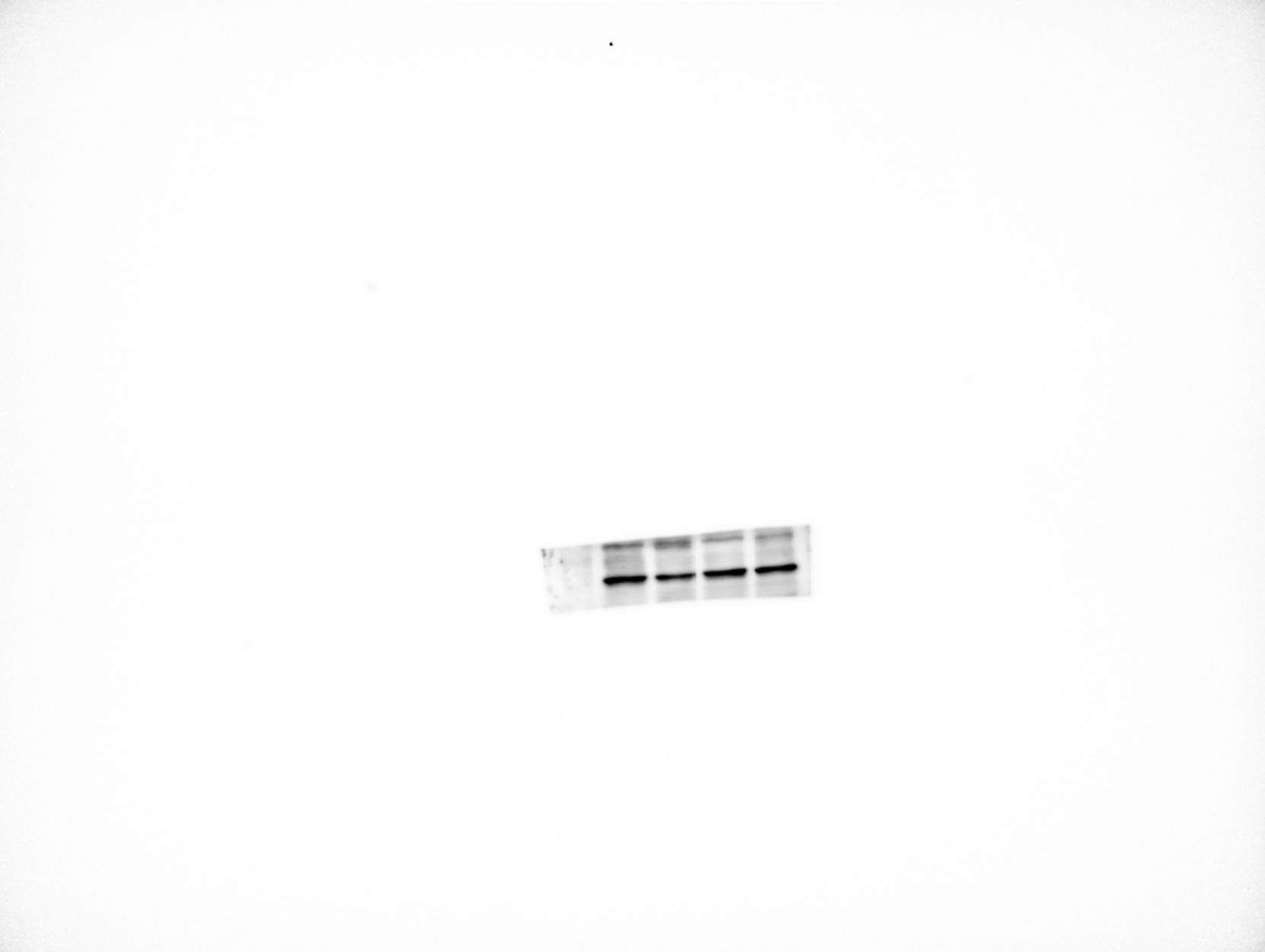

Supplement: Supplementary file 2 [file DataSheet1.ZIP › original WB photo/Ppara┴/Ppara-F_pub.jpg]

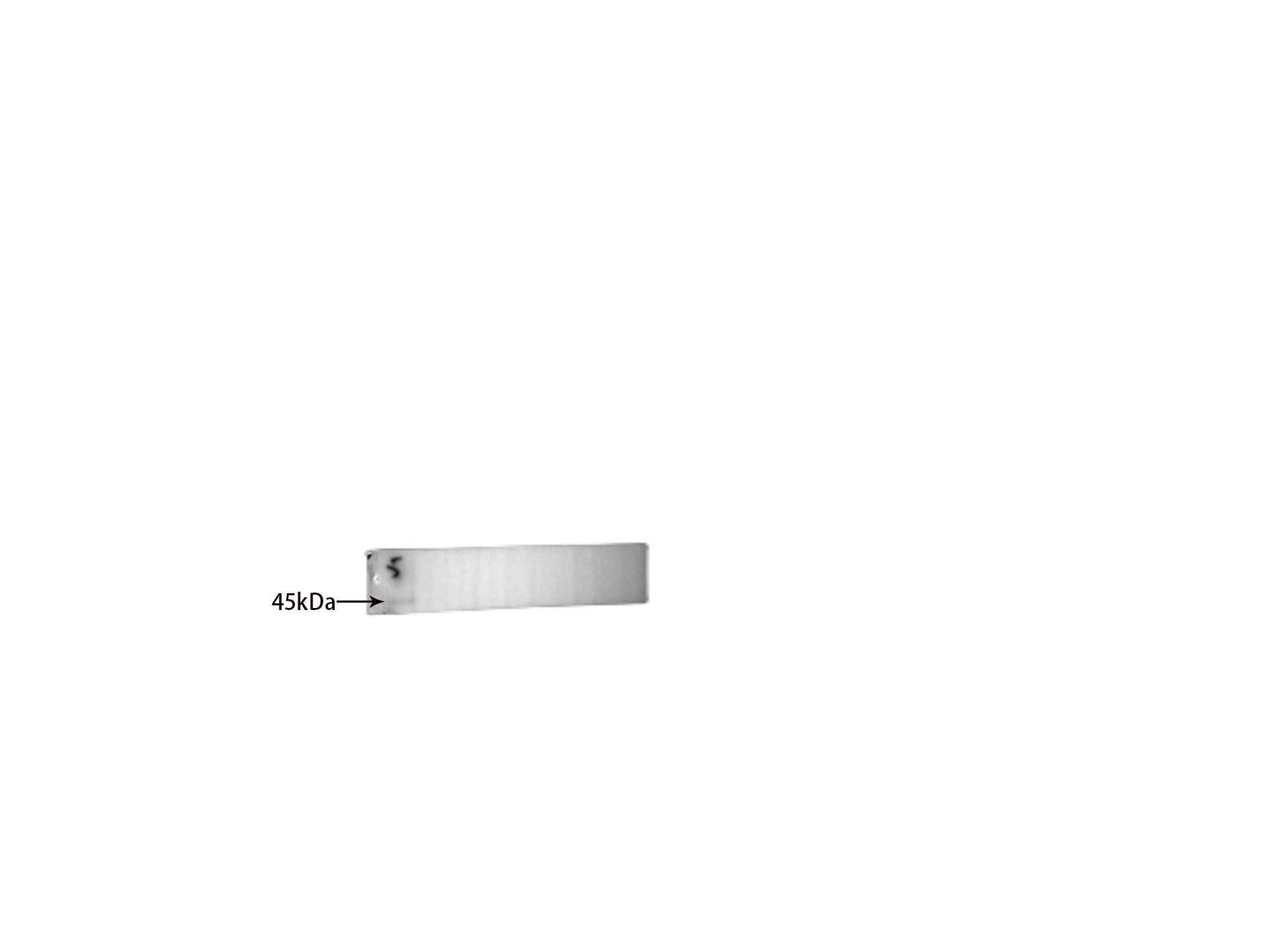

Supplement: Supplementary file 2 [file DataSheet1.ZIP › original WB photo/Ppara┴/Ppara-S-marker_pub.jpg]

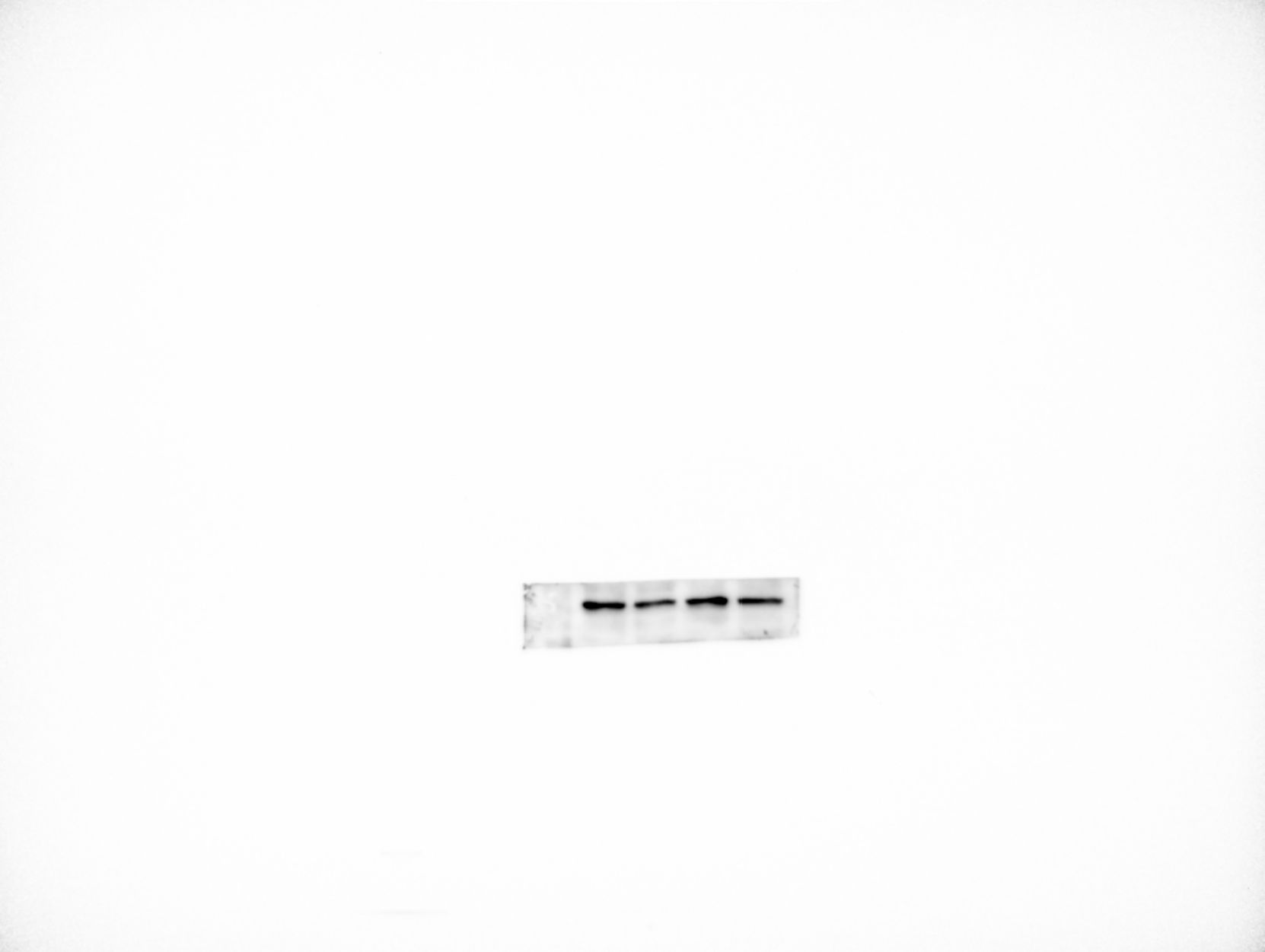

Supplement: Supplementary file 2 [file DataSheet1.ZIP › original WB photo/Ppara┴/Ppara-S_pub.jpg]

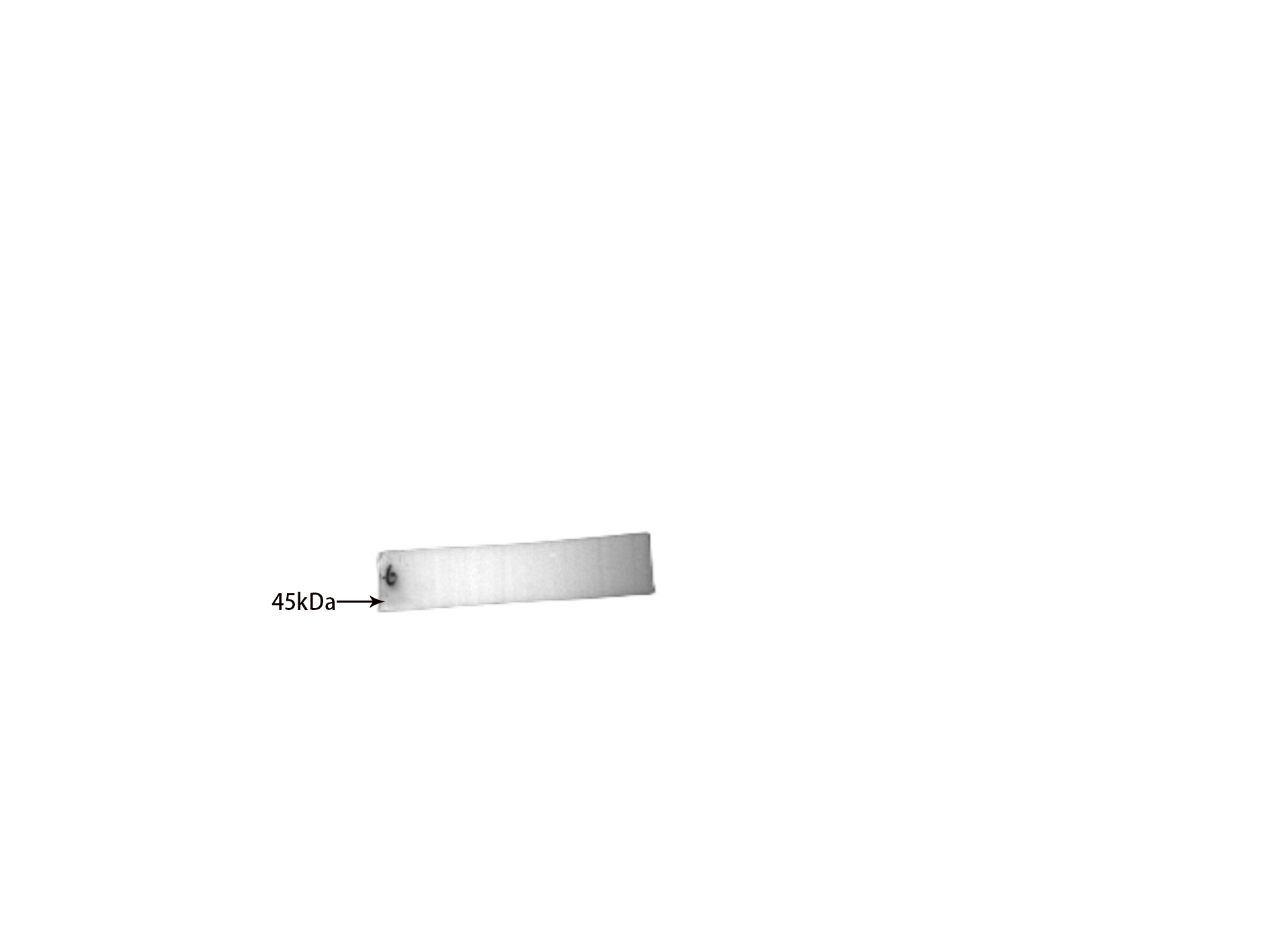

Supplement: Supplementary file 2 [file DataSheet1.ZIP › original WB photo/Ppara┴/Ppara-T-marker_pub.jpg]

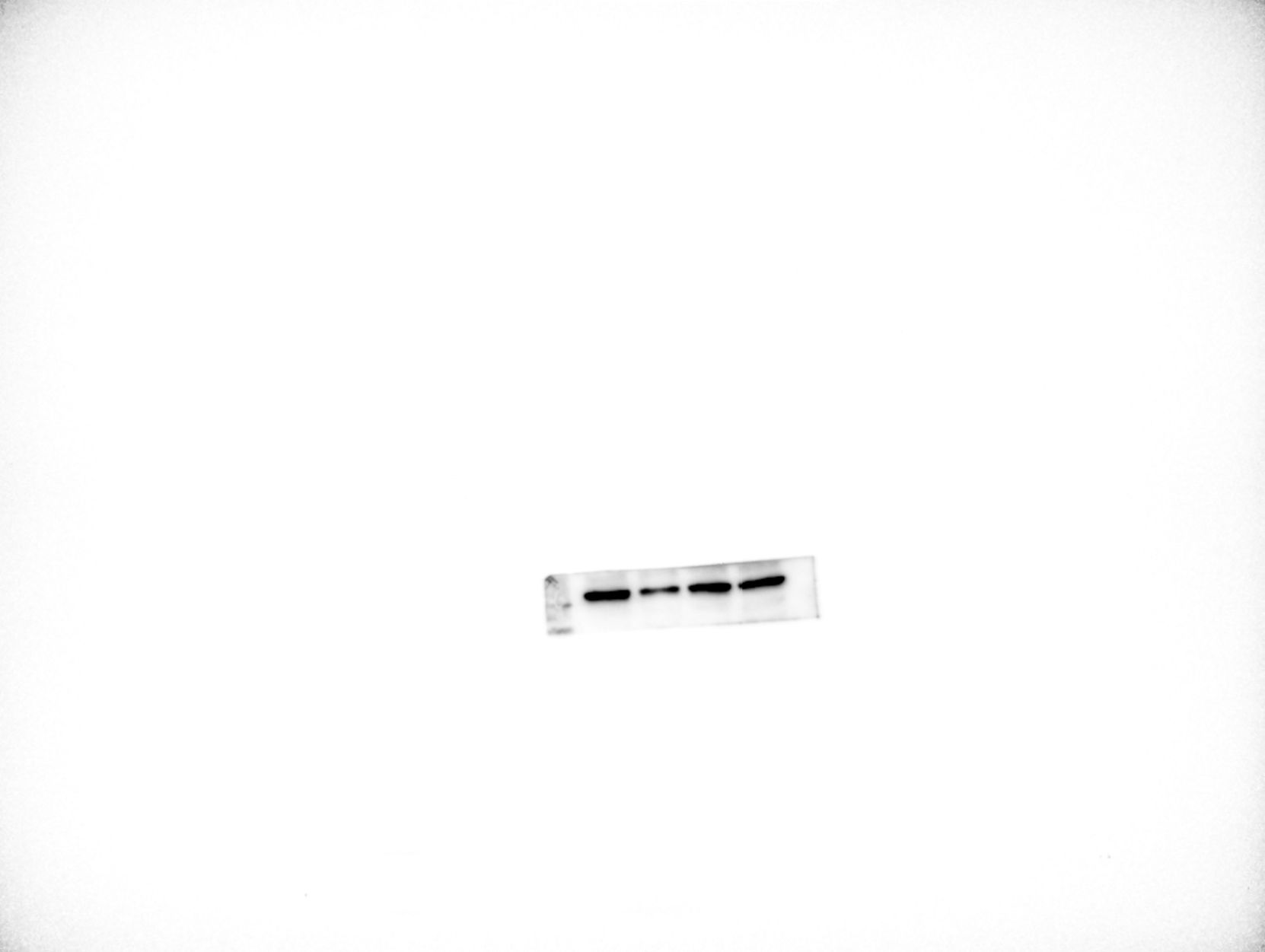

Supplement: Supplementary file 2 [file DataSheet1.ZIP › original WB photo/Ppara┴/Ppara-T_pub.jpg]

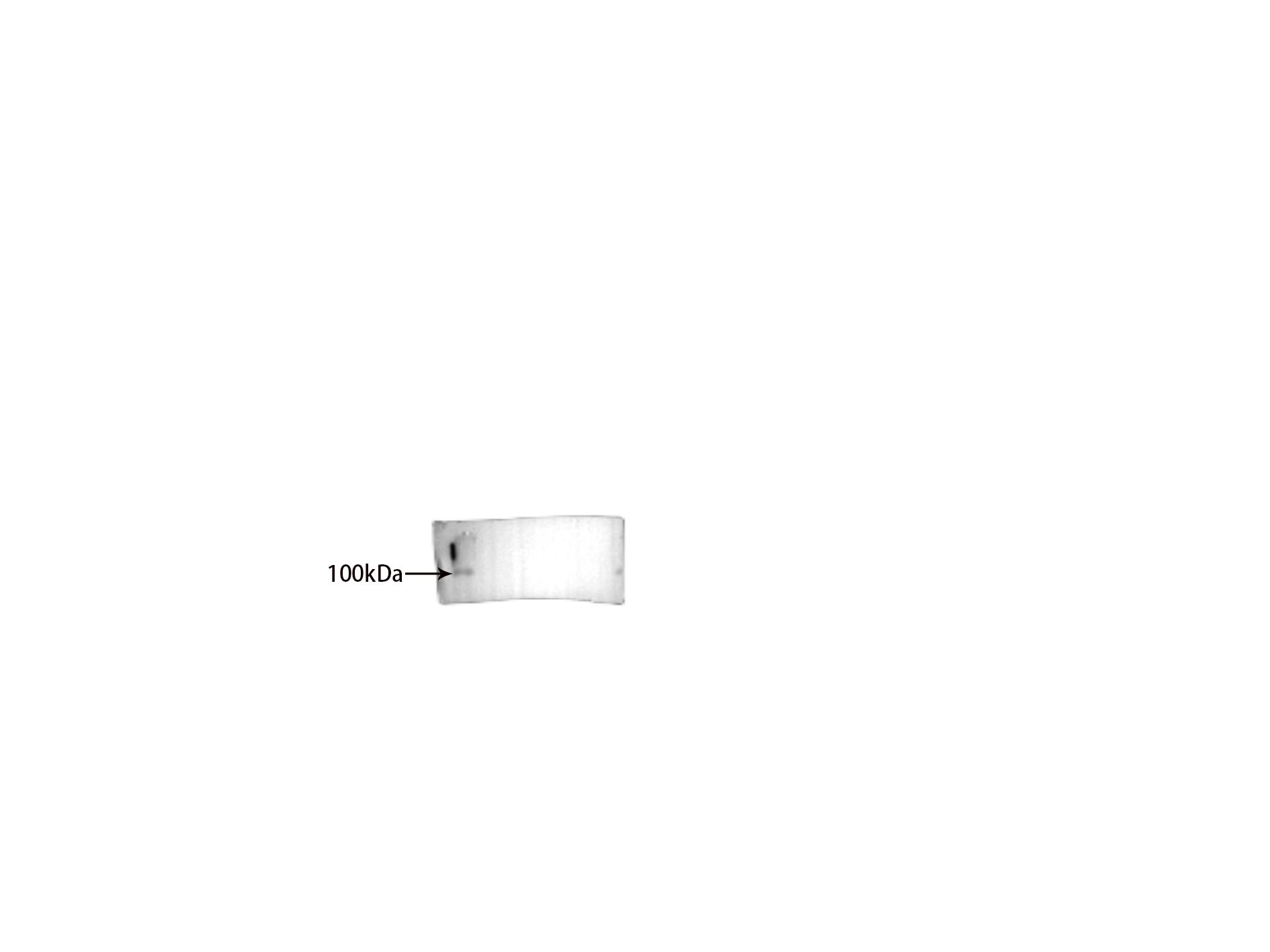

Supplement: Supplementary file 2 [file DataSheet1.ZIP › original WB photo/SIRT1/Sirt-F-marker_pub.jpg]

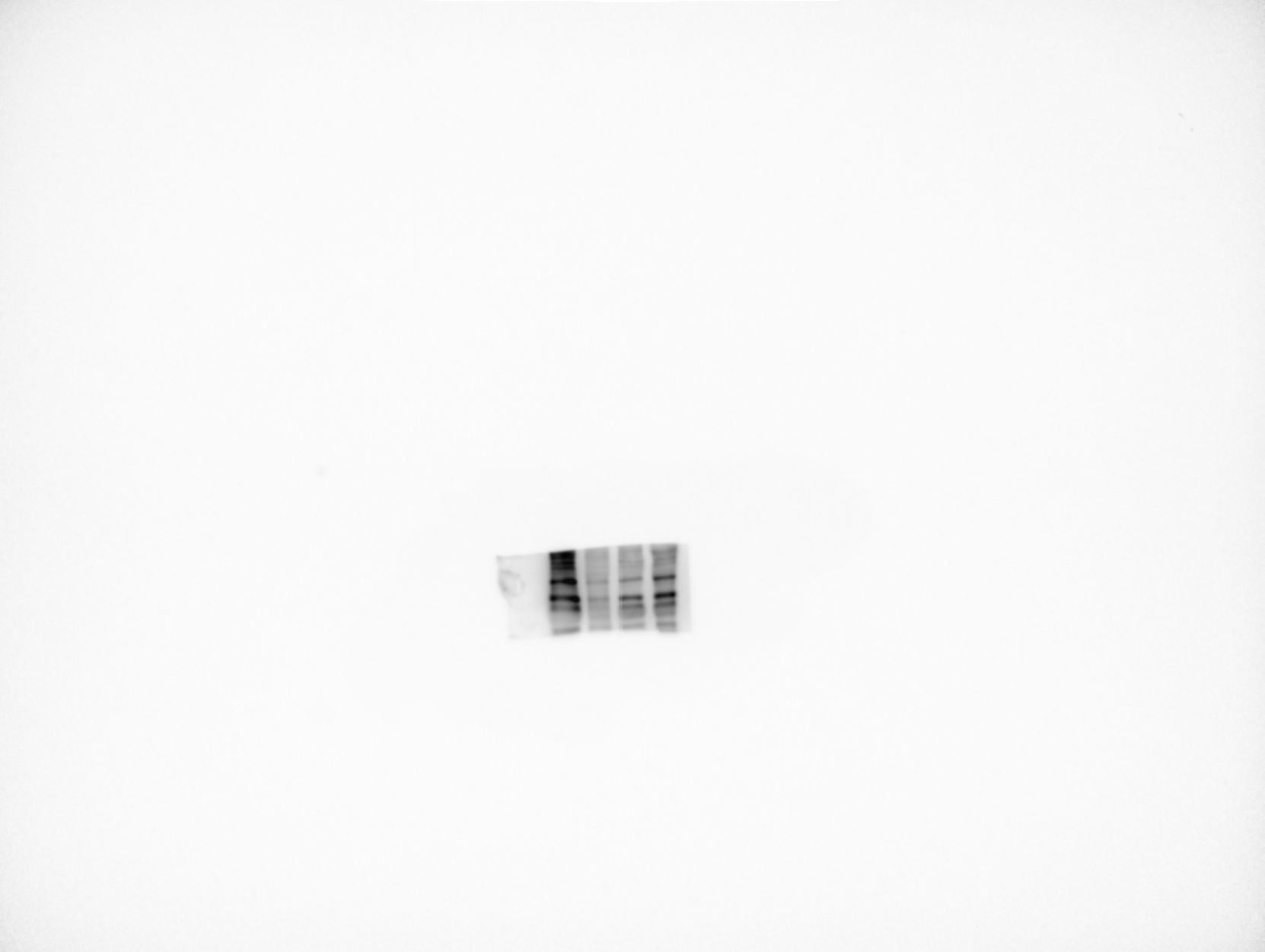

Supplement: Supplementary file 2 [file DataSheet1.ZIP › original WB photo/SIRT1/Sirt-F_pub.jpg]

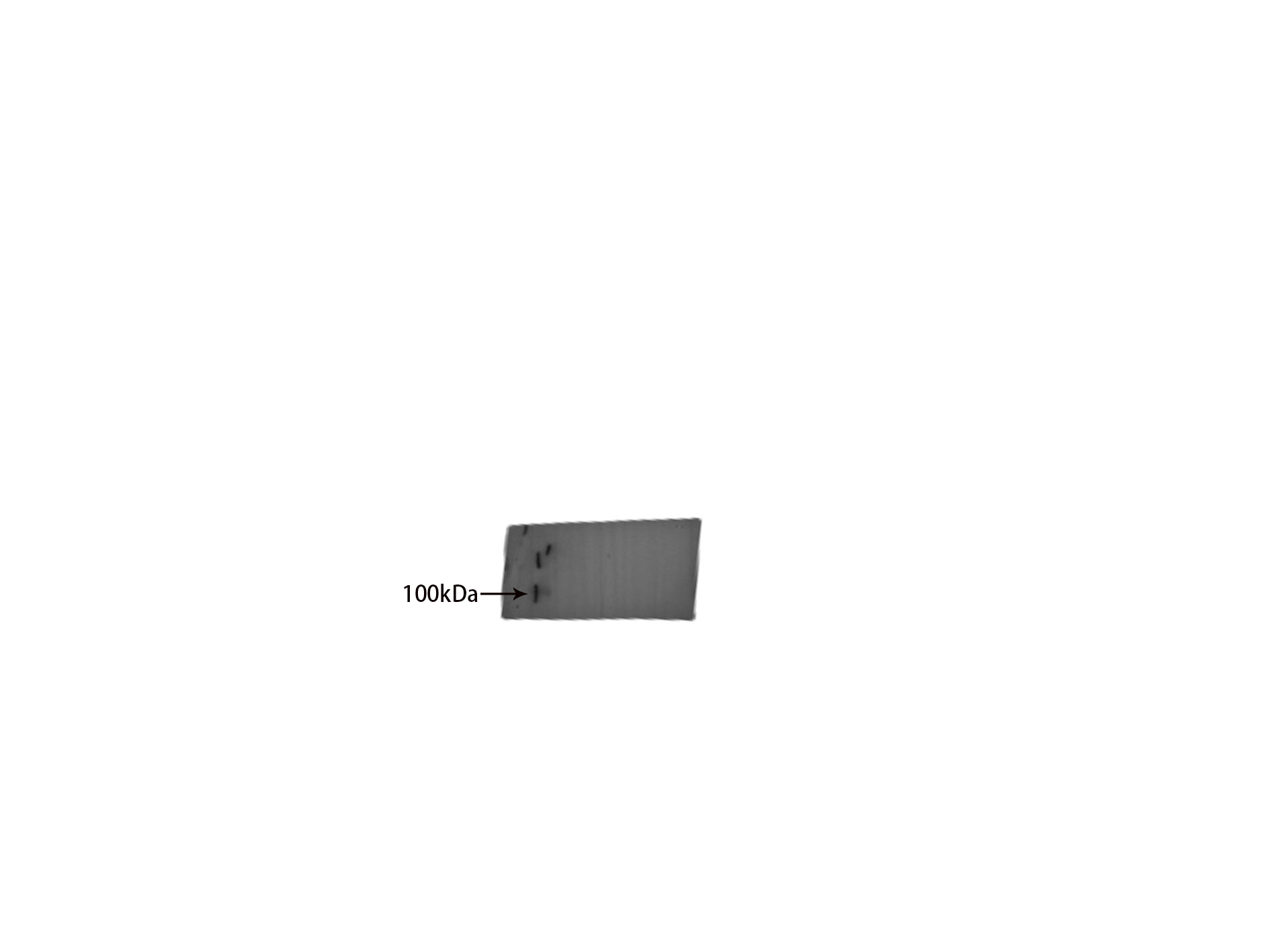

Supplement: Supplementary file 2 [file DataSheet1.ZIP › original WB photo/SIRT1/Sirt-S-marker_pub.jpg]

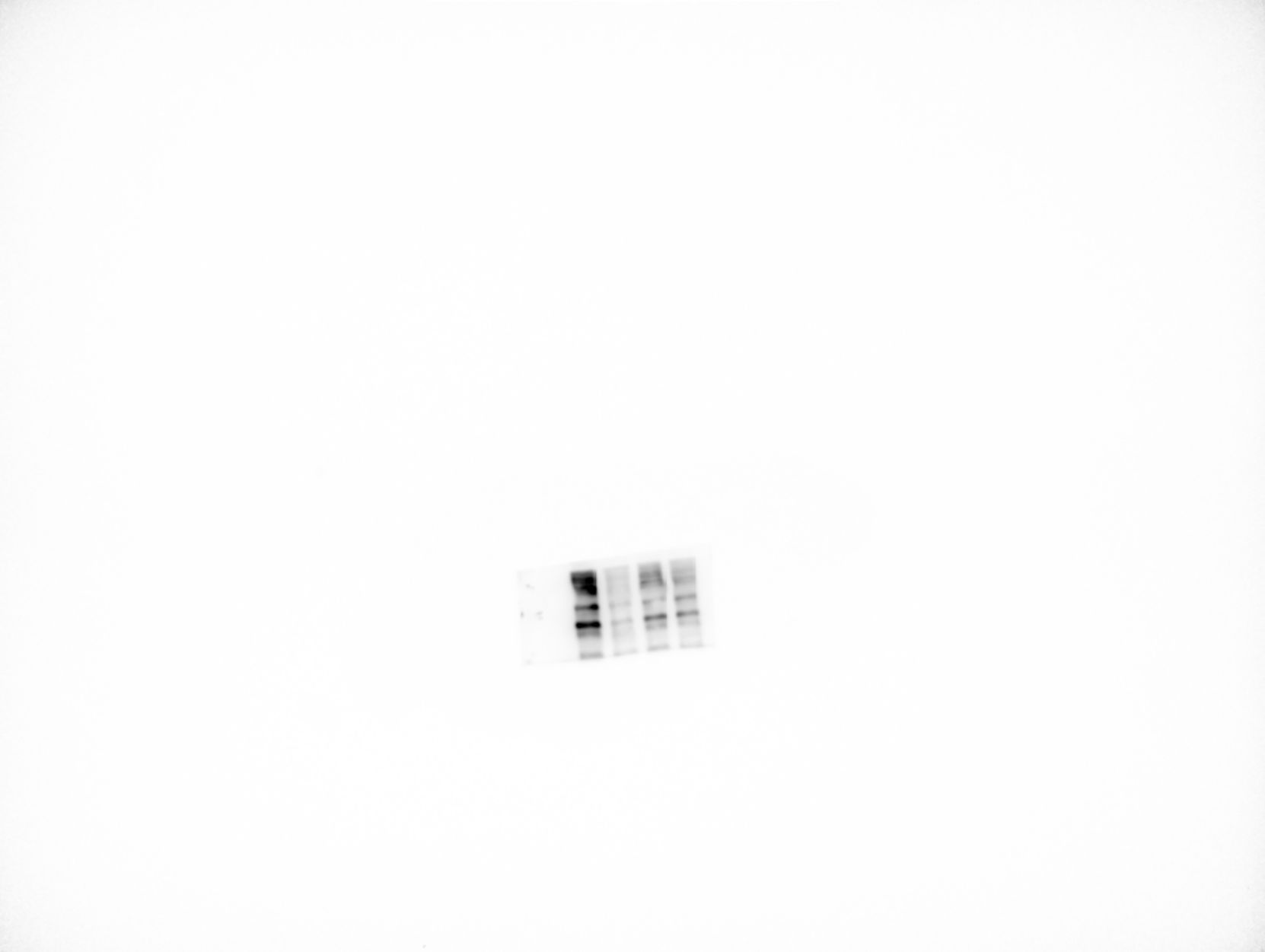

Supplement: Supplementary file 2 [file DataSheet1.ZIP › original WB photo/SIRT1/Sirt-S_pub.jpg]

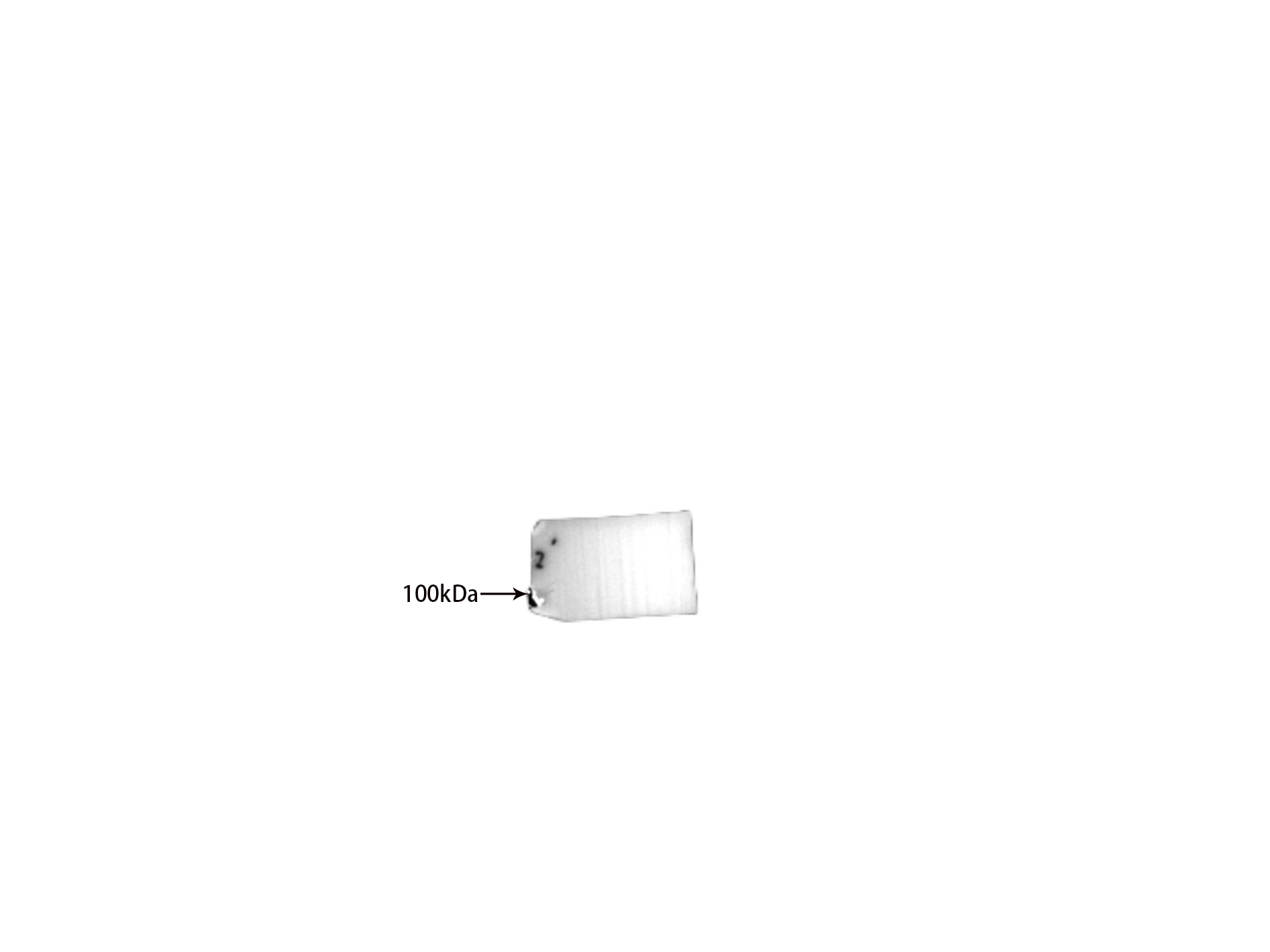

Supplement: Supplementary file 2 [file DataSheet1.ZIP › original WB photo/SIRT1/Sirt-T-marker_pub.jpg]

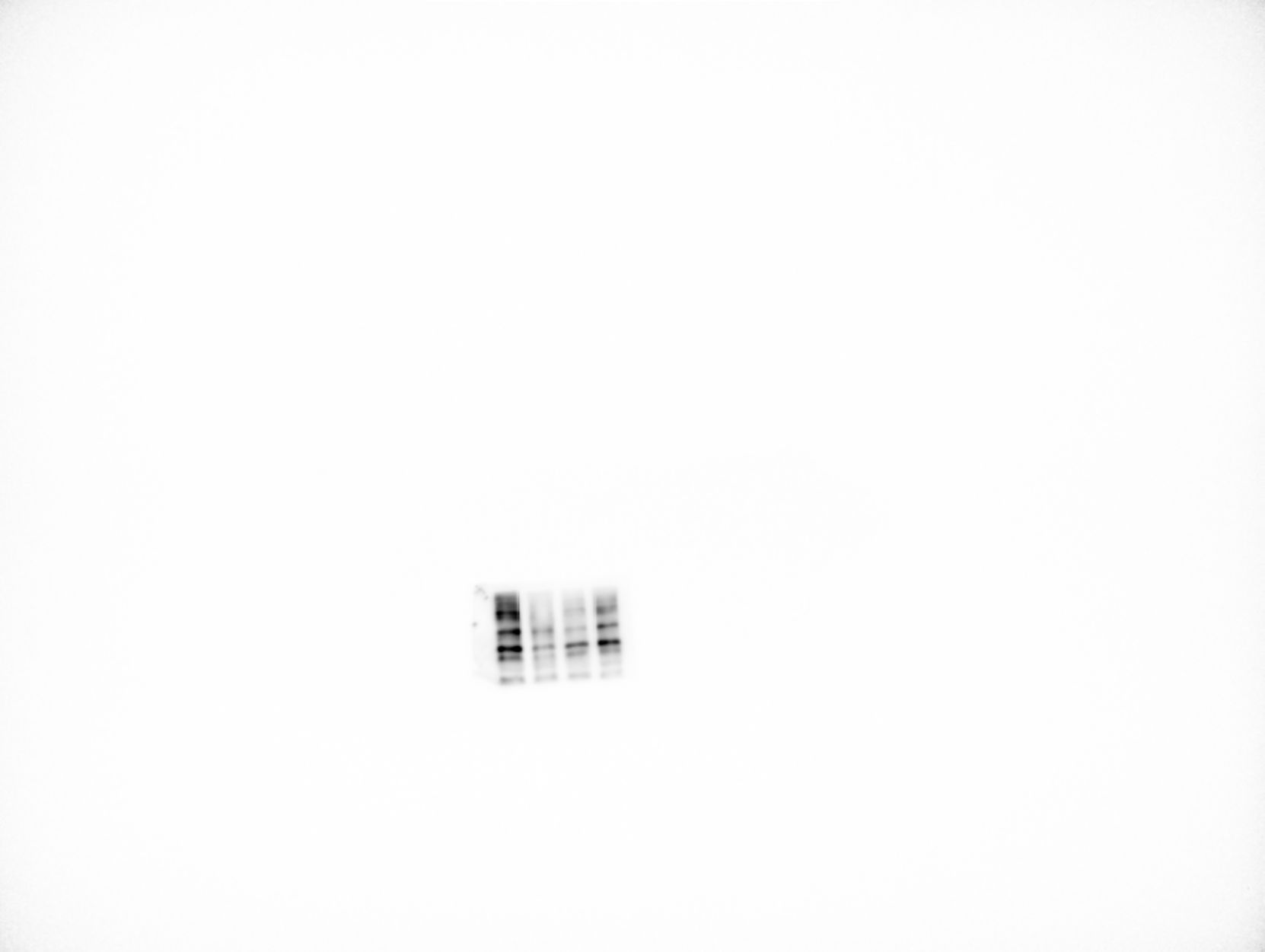

Supplement: Supplementary file 2 [file DataSheet1.ZIP › original WB photo/SIRT1/Sirt-T_pub.jpg]

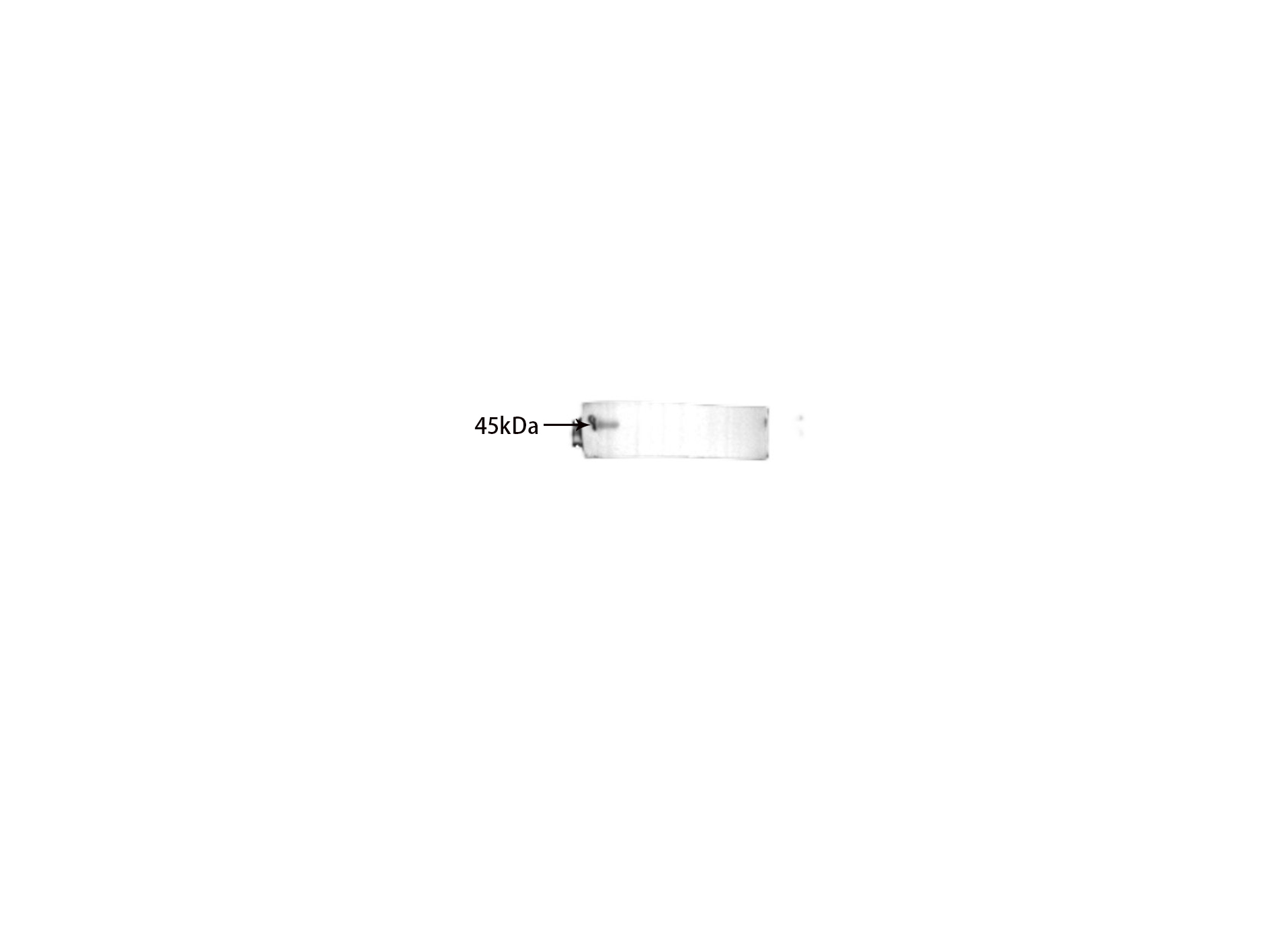

Supplement: Supplementary file 2 [file DataSheet1.ZIP › original WB photo/UCP3/UCP3-F-marker_pub.jpg]

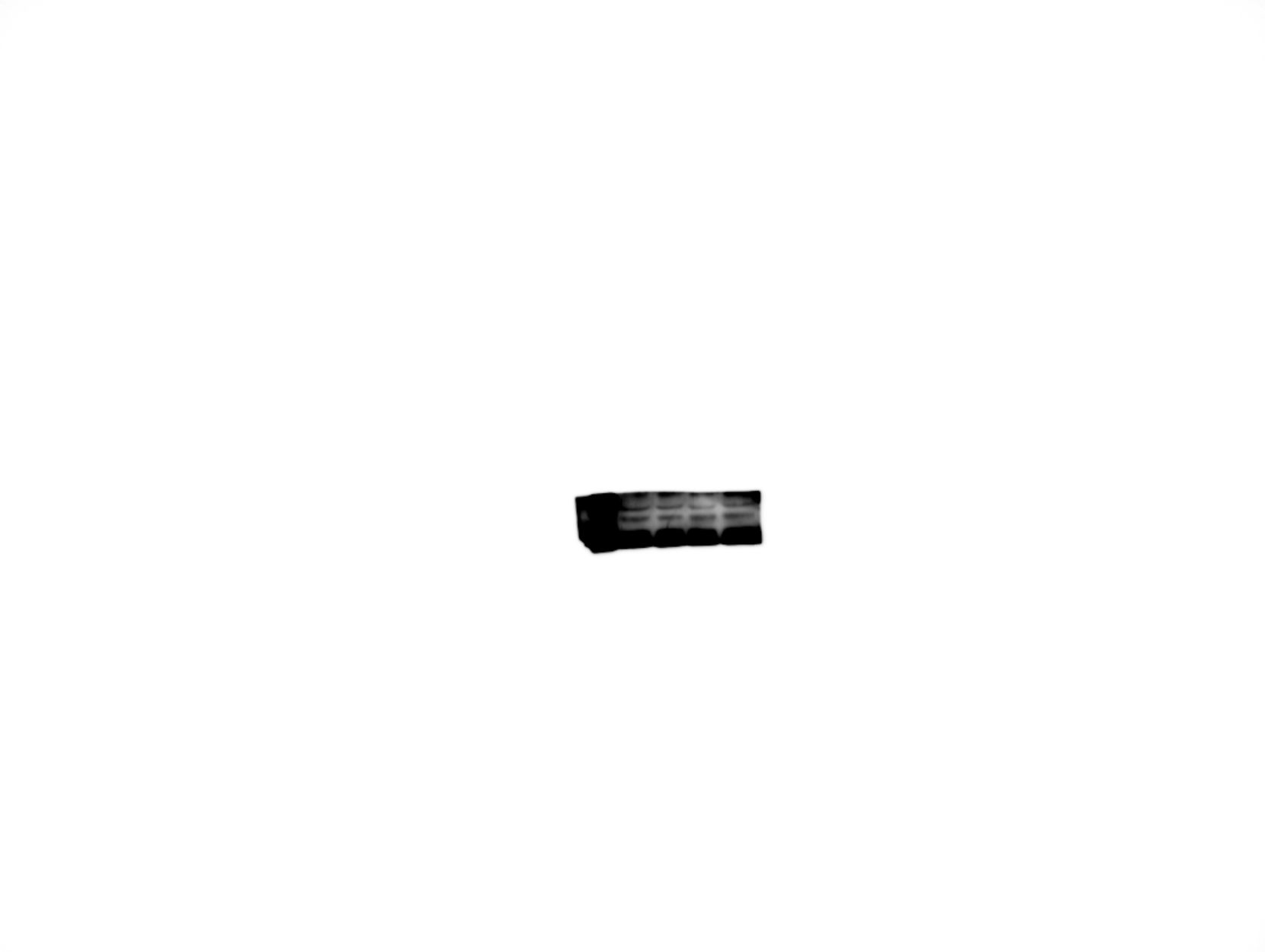

Supplement: Supplementary file 2 [file DataSheet1.ZIP › original WB photo/UCP3/UCP3-F_pub.jpg]

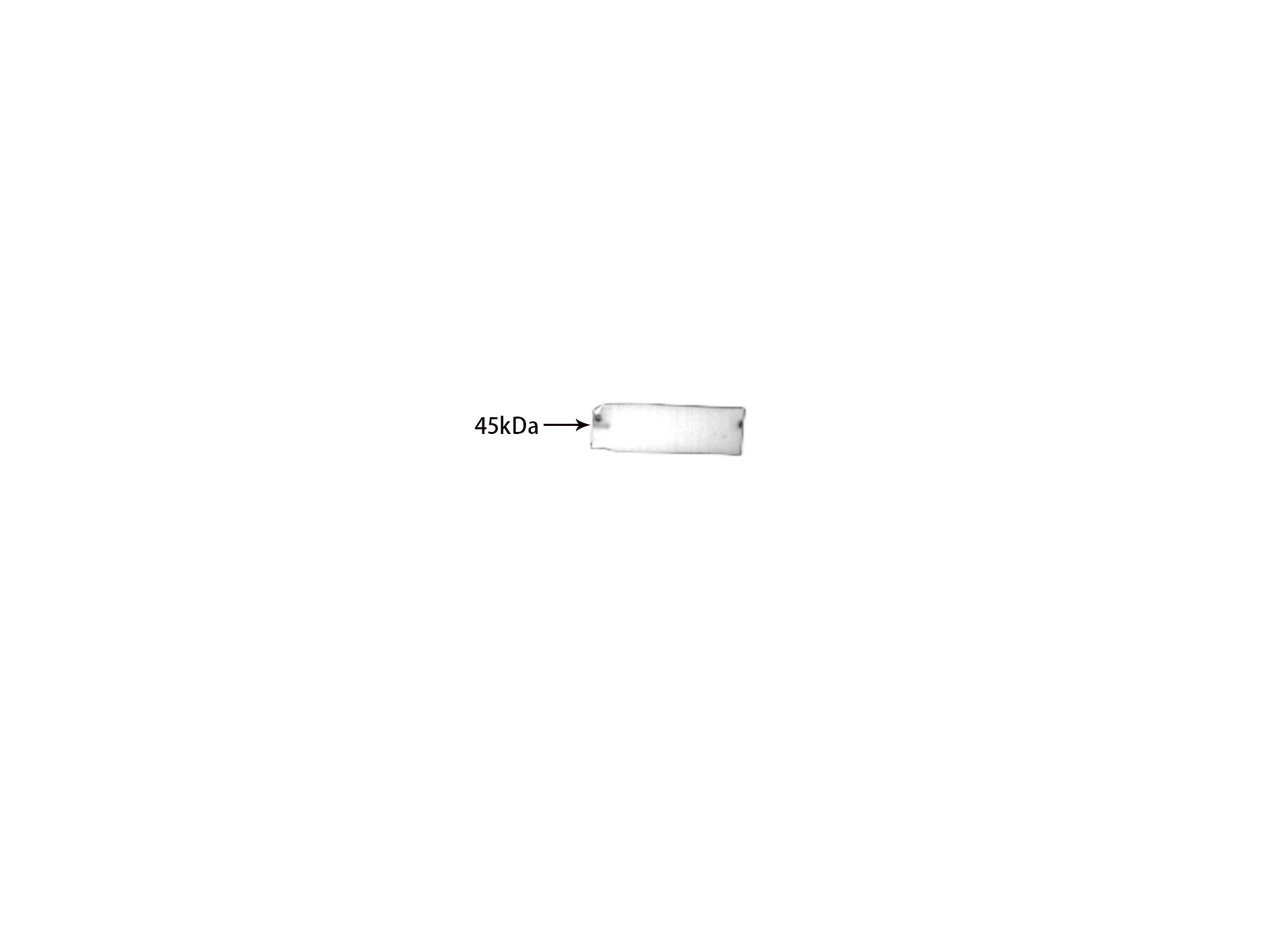

Supplement: Supplementary file 2 [file DataSheet1.ZIP › original WB photo/UCP3/UCP3-S-marker_pub.jpg]

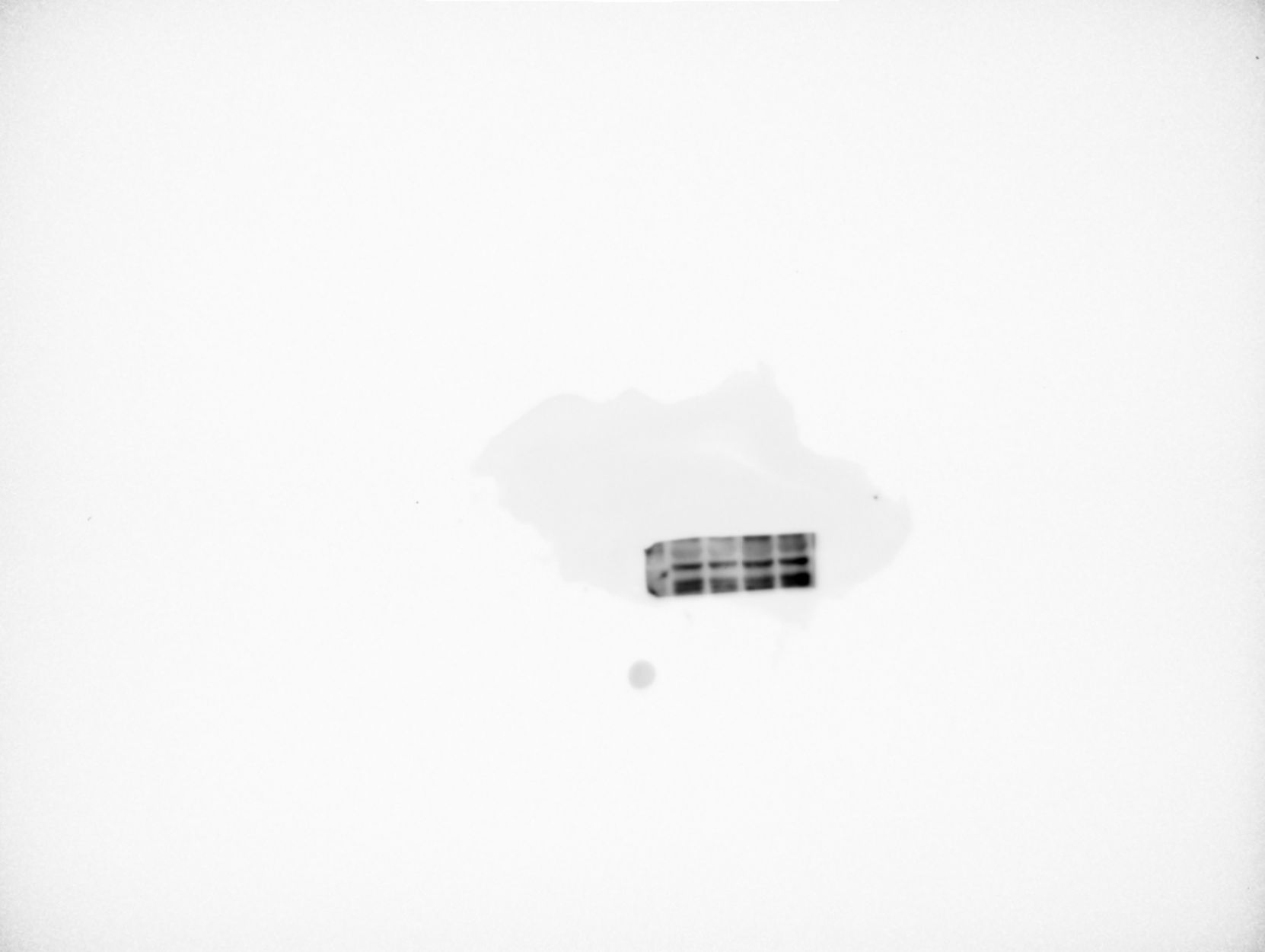

Supplement: Supplementary file 2 [file DataSheet1.ZIP › original WB photo/UCP3/UCP3-S_pub.jpg]

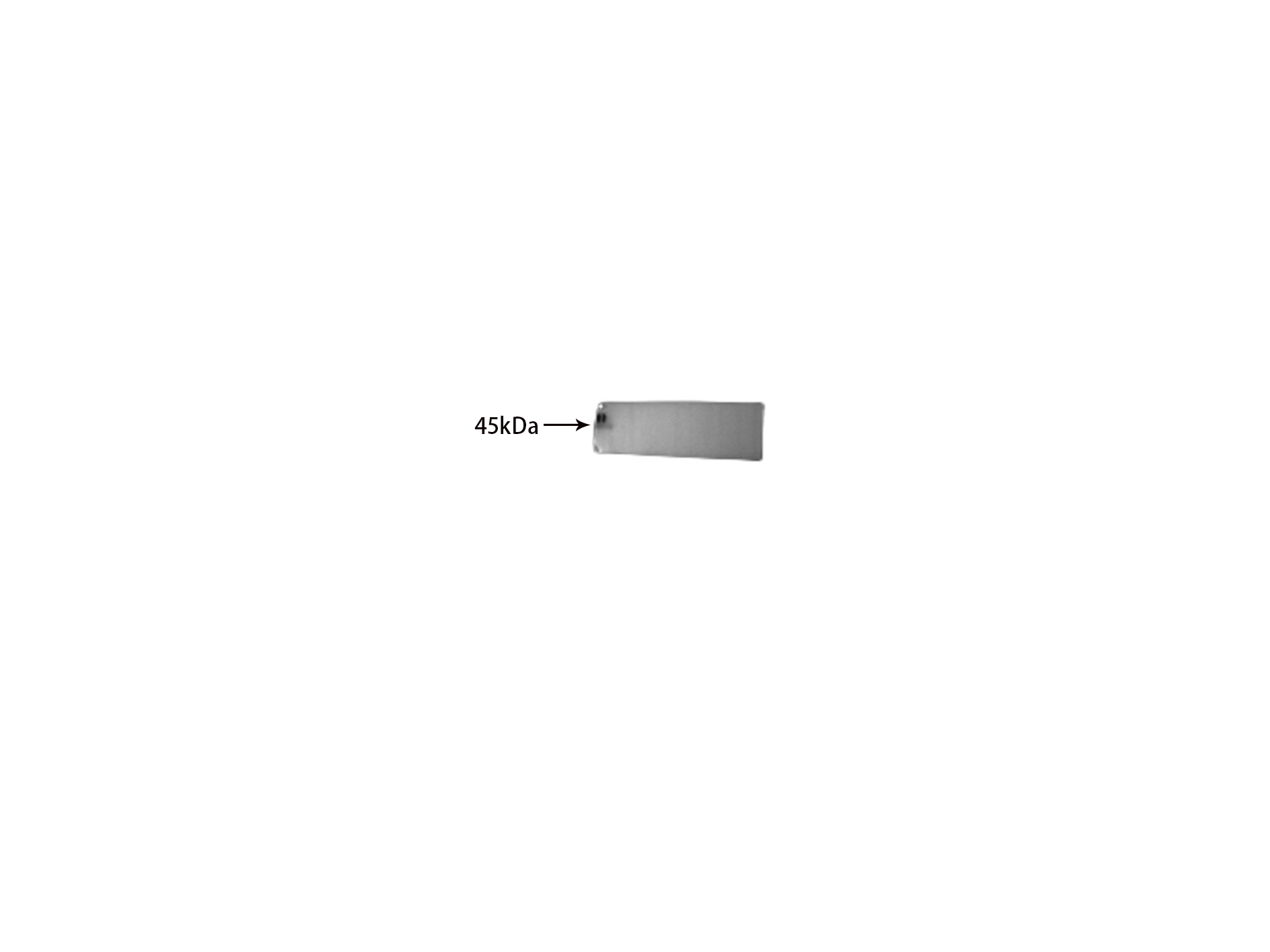

Supplement: Supplementary file 2 [file DataSheet1.ZIP › original WB photo/UCP3/UCP3-T-marker_pub.jpg]

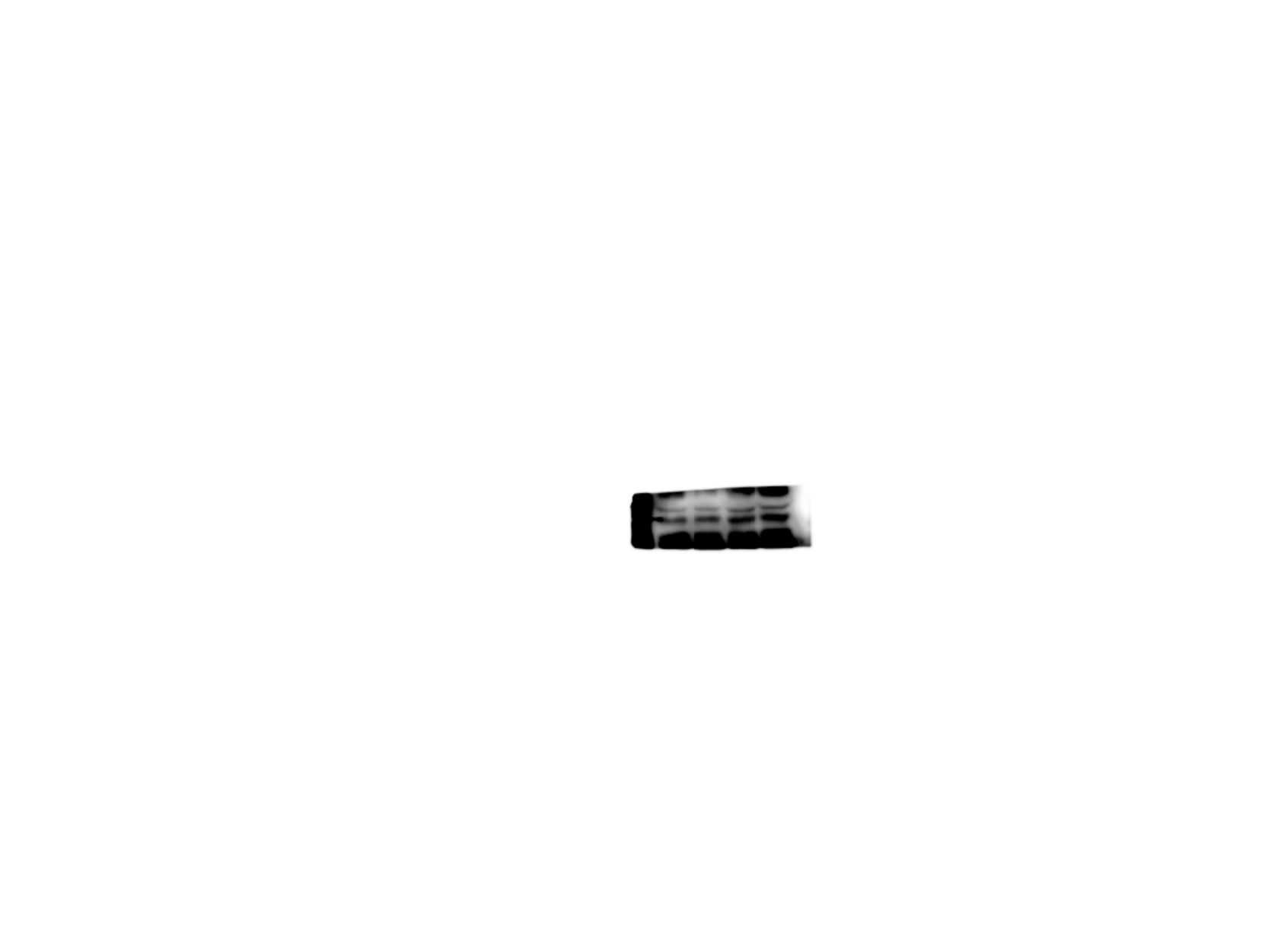

Supplement: Supplementary file 2 [file DataSheet1.ZIP › original WB photo/UCP3/UCP3-T_pub.jpg]

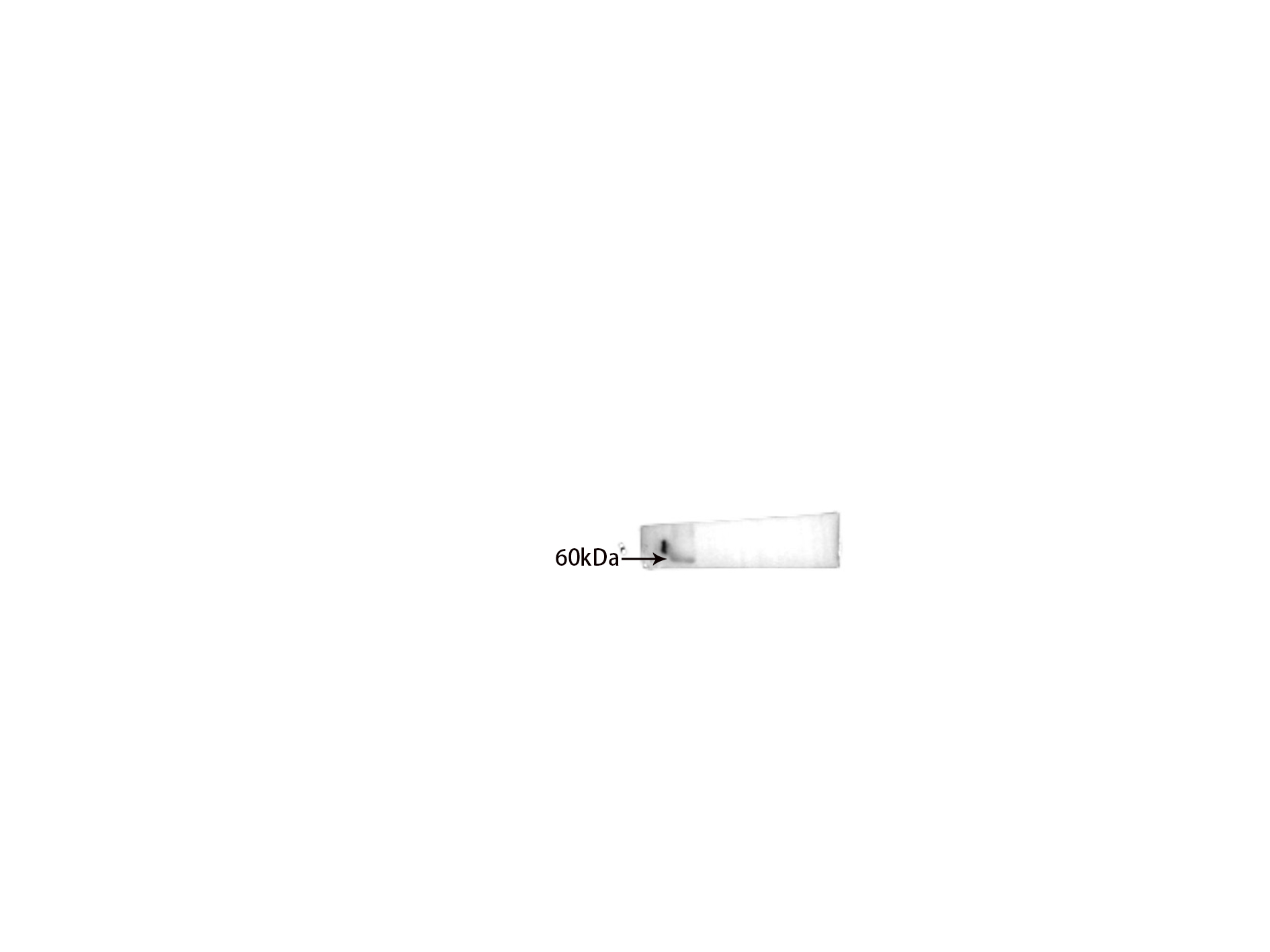

Supplement: Supplementary file 2 [file DataSheet1.ZIP › original WB photo/pAMPKa┴-AMPKa┴/AMPKa-F-marker_pub.jpg]

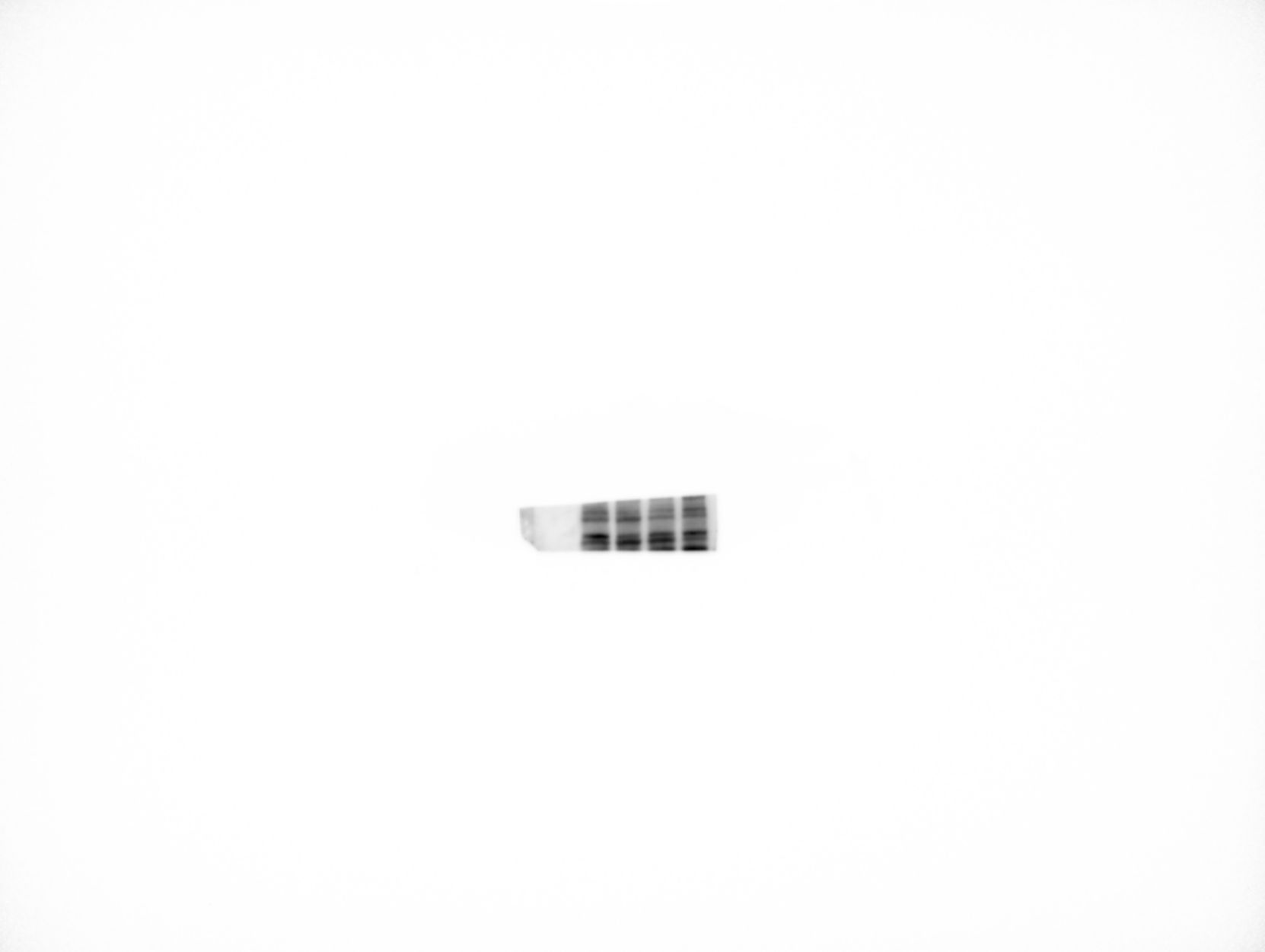

Supplement: Supplementary file 2 [file DataSheet1.ZIP › original WB photo/pAMPKa┴-AMPKa┴/AMPKa-F_pub.jpg]

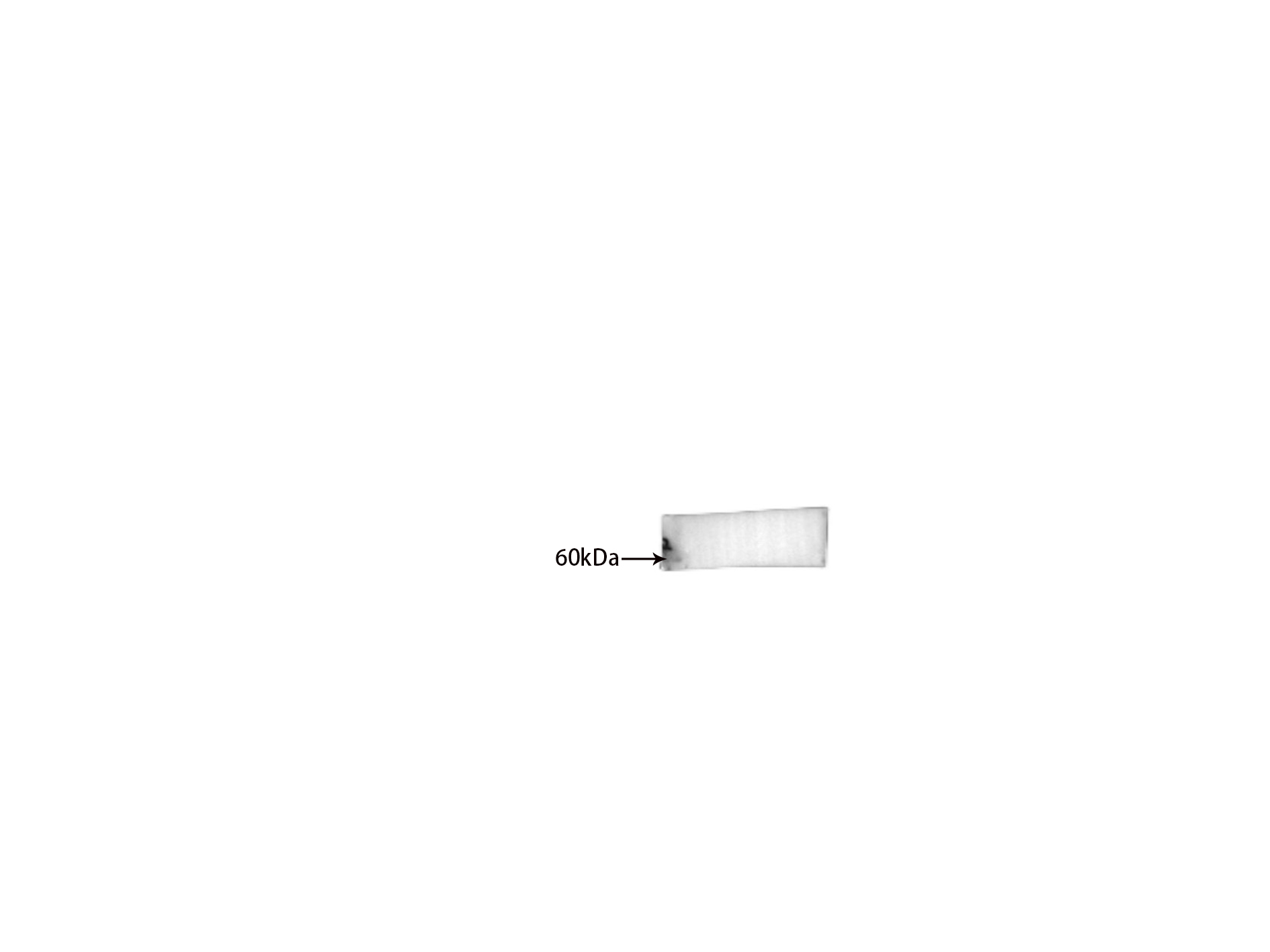

Supplement: Supplementary file 2 [file DataSheet1.ZIP › original WB photo/pAMPKa┴-AMPKa┴/AMPKa-S-marker_pub.jpg]

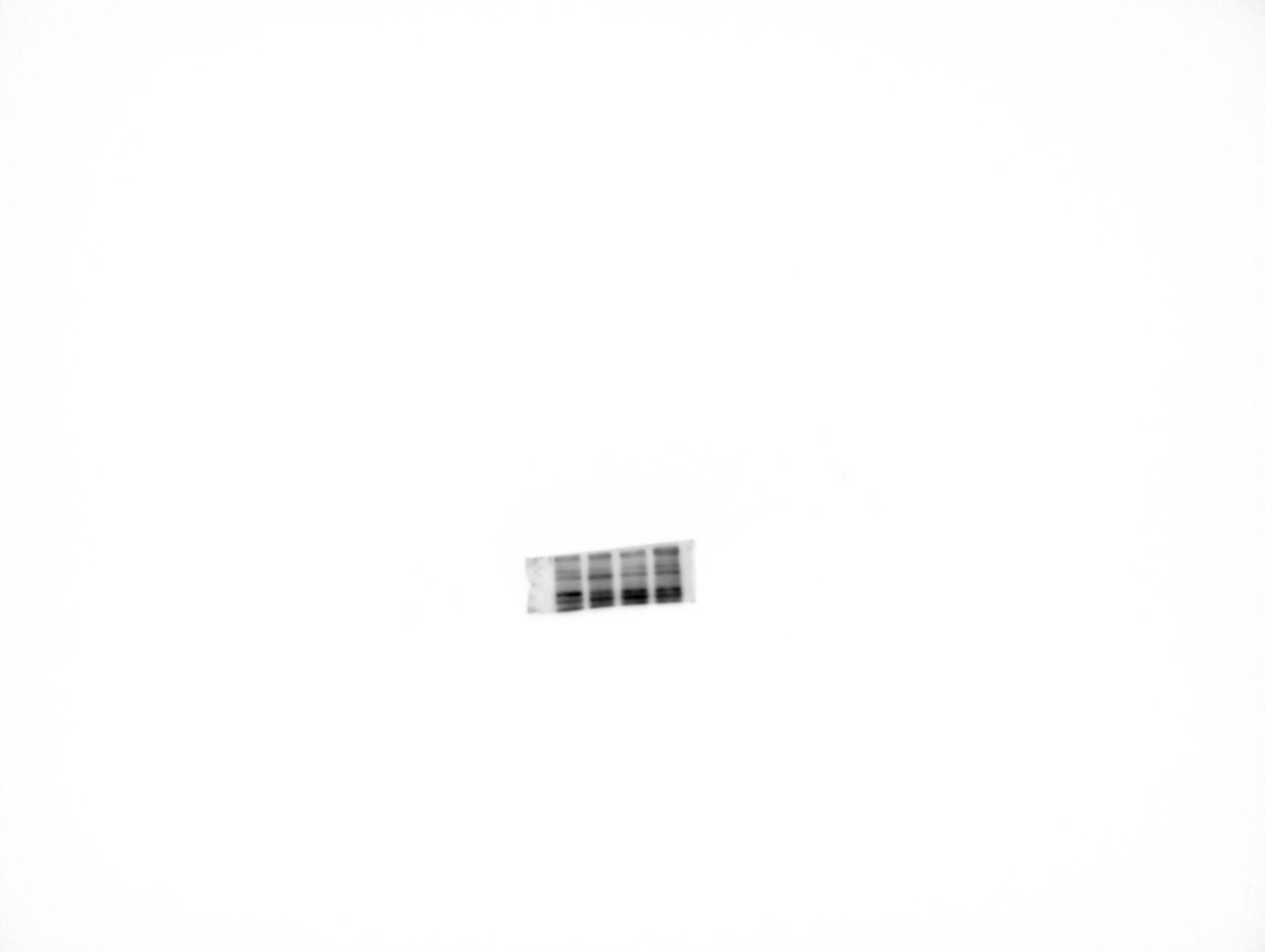

Supplement: Supplementary file 2 [file DataSheet1.ZIP › original WB photo/pAMPKa┴-AMPKa┴/AMPKa-S_pub.jpg]

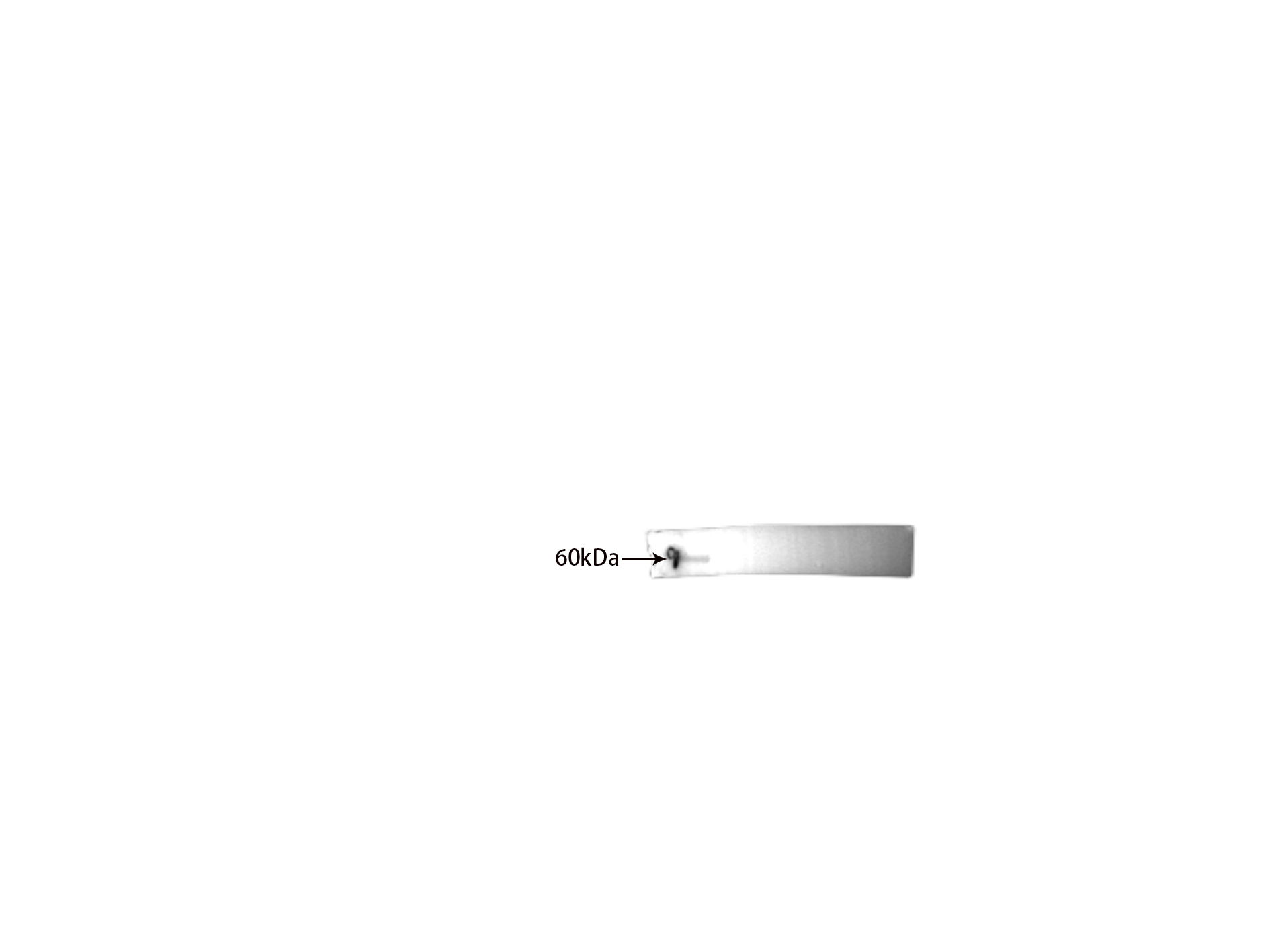

Supplement: Supplementary file 2 [file DataSheet1.ZIP › original WB photo/pAMPKa┴-AMPKa┴/AMPKa-T-marker_pub.jpg]

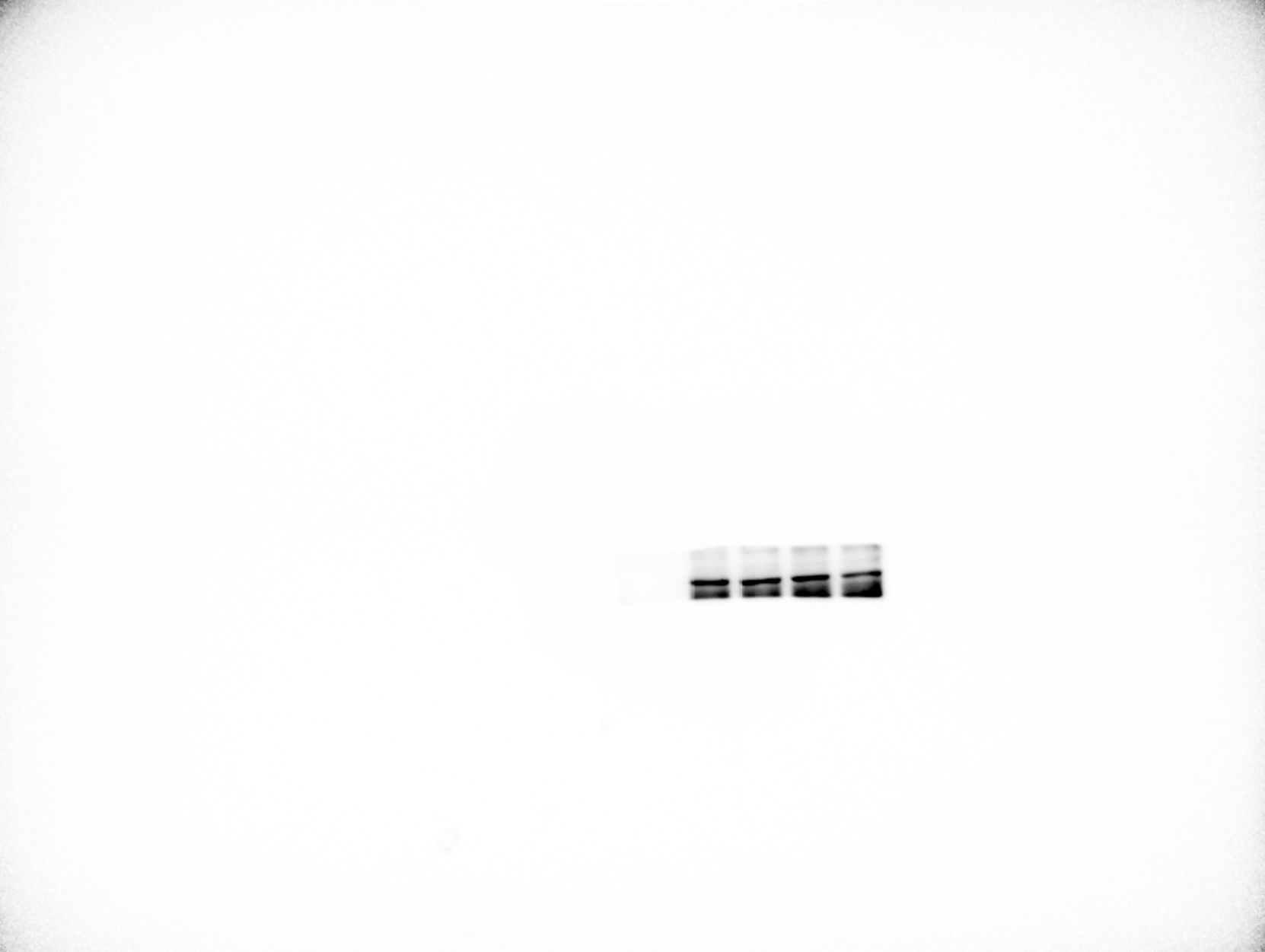

Supplement: Supplementary file 2 [file DataSheet1.ZIP › original WB photo/pAMPKa┴-AMPKa┴/AMPKa-T_pub.jpg]

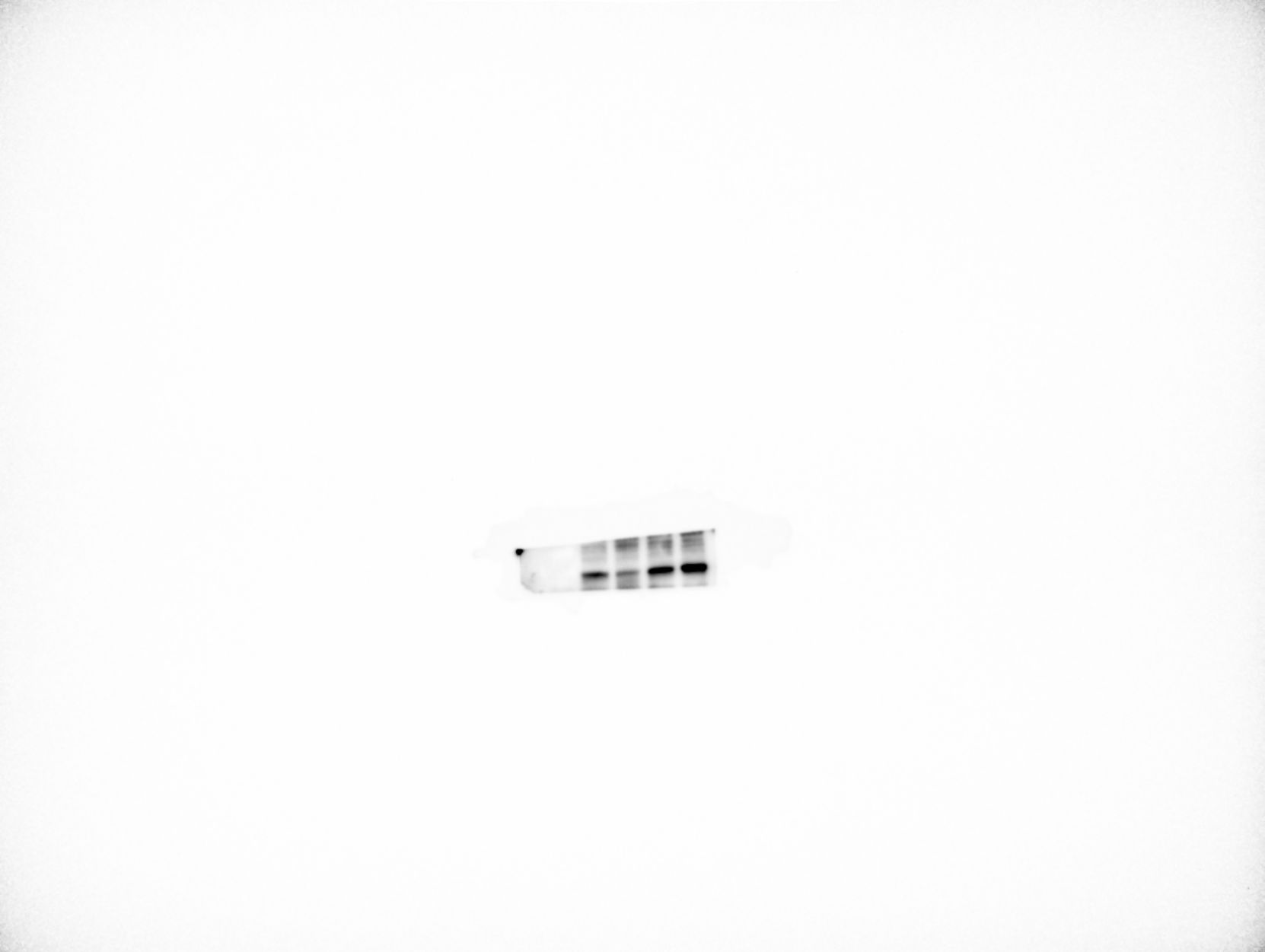

Supplement: Supplementary file 2 [file DataSheet1.ZIP › original WB photo/pAMPKa┴-AMPKa┴/pAMPK-F_pub.jpg]

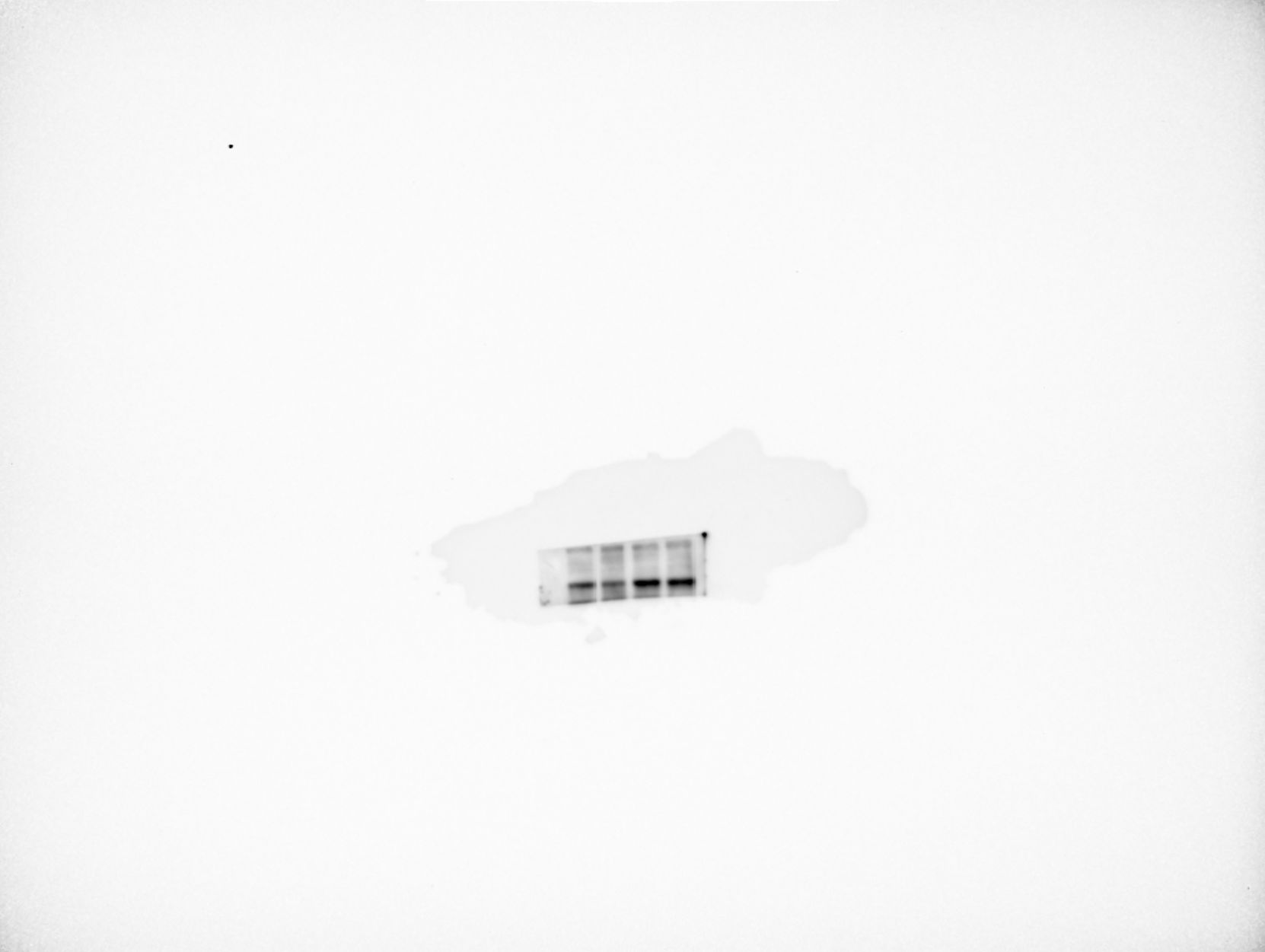

Supplement: Supplementary file 2 [file DataSheet1.ZIP › original WB photo/pAMPKa┴-AMPKa┴/pAMPK-S_pub.jpg]

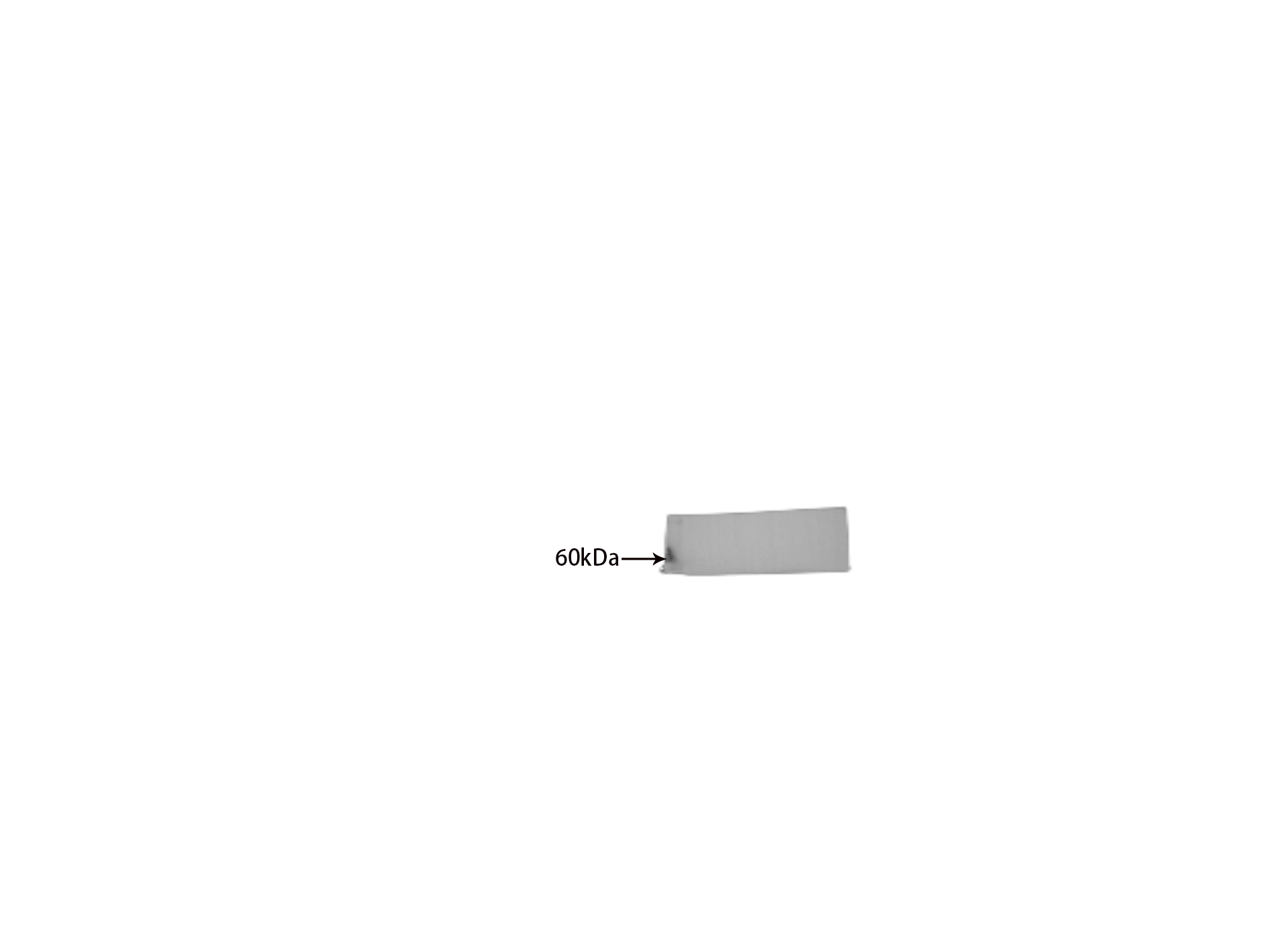

Supplement: Supplementary file 2 [file DataSheet1.ZIP › original WB photo/pAMPKa┴-AMPKa┴/pAMPK-T-marker_pub.jpg]

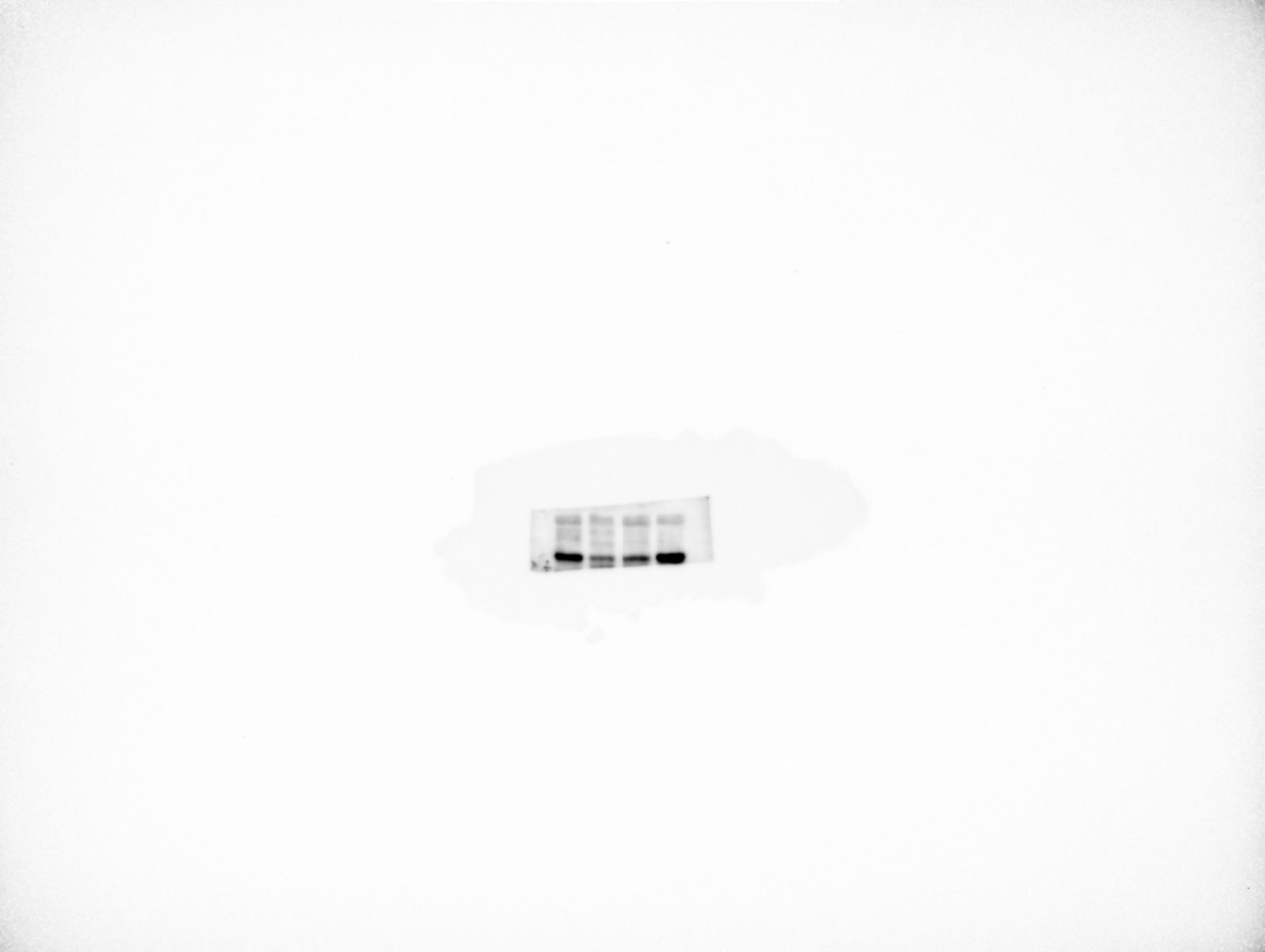

Supplement: Supplementary file 2 [file DataSheet1.ZIP › original WB photo/pAMPKa┴-AMPKa┴/pAMPK-T_pub.jpg]
